# SNCA Triplication Parkinson's Patient's iPSC-derived DA Neurons Accumulate α-Synuclein and Are Susceptible to Oxidative Stress

**DOI:** 10.1371/journal.pone.0026159

**Published:** 2011-11-16

**Authors:** Blake Byers, Branden Cord, Ha Nam Nguyen, Birgitt Schüle, Lief Fenno, Patrick C. Lee, Karl Deisseroth, J. William Langston, Renee Reijo Pera, Theo D. Palmer

**Affiliations:** 1 Department of Bioengineering, Stanford University, Stanford, California, United States of America; 2 Institute for Stem Cell Biology and Regenerative Medicine, Stanford University, Stanford, California, United States of America; 3 Department of Obstetrics and Gynecology, Stanford University, Stanford, California, United States of America; 4 Department of Neurosurgery, Stanford University, Stanford, California, United States of America; 5 Parkinson's Institute and Clinical Center, Sunnyvale, California, United States of America; 6 Department of Neuroscience, Stanford University, Stanford, California, United States of America; 7 Howard Hughes Medical Institute, Stanford University, Stanford, California, United States of America; 8 Department of Psychiatry and Behavioral Sciences, Stanford, California, United States of America; University of California, Merced, United States of America

## Abstract

Parkinson's disease (PD) is an incurable age-related neurodegenerative disorder affecting both the central and peripheral nervous systems. Although common, the etiology of PD remains poorly understood. Genetic studies infer that the disease results from a complex interaction between genetics and environment and there is growing evidence that PD may represent a constellation of diseases with overlapping yet distinct underlying mechanisms. Novel clinical approaches will require a better understanding of the mechanisms at work within an individual as well as methods to identify the specific array of mechanisms that have contributed to the disease. Induced pluripotent stem cell (iPSC) strategies provide an opportunity to directly study the affected neuronal subtypes in a given patient. Here we report the generation of iPSC-derived midbrain dopaminergic neurons from a patient with a triplication in the α-synuclein gene (*SNCA*). We observed that the iPSCs readily differentiated into functional neurons. Importantly, the PD-affected line exhibited disease-related phenotypes in culture: accumulation of α-synuclein, inherent overexpression of markers of oxidative stress, and sensitivity to peroxide induced oxidative stress. These findings show that the dominantly-acting PD mutation is intrinsically capable of perturbing normal cell function in culture and confirm that these features reflect, at least in part, a cell autonomous disease process that is independent of exposure to the entire complexity of the diseased brain.

## Introduction

Parkinson's Disease (PD) is a debilitating neurodegenerative disorder characterized by the loss of neurons in both the peripheral and central nervous system [1], [2], [3], [4], [5], [6]. The majority of cases result from unknown etiology causing widespread neuron loss in the midbrain, cerebral cortex, olfactory bulb, and peripheral nervous system. Neuron degeneration is pronounced in the dopaminergic neurons of the substantia nigra pars compacta leading to the diagnostic clinical features of bradykinesia, rigidity and tremor [1], [2], [3], [4], [5], [6], [7].

Genetic studies have implicated several potential mechanisms but the etiology of Parkinson's disease remains obscure. In a large cohort of patients, Lewy bodies and Lewy neurites form in CNS and autonomic peripheral nervous system (PNS) neurons. These large intracellular proteinaceous inclusions are rich in α-synuclein and ubiquitin, an observation that suggests a role for α-synuclein and the proteasome in the molecular development of sporadic and genetic PD [8]. However, not all patients with PD develop Lewy bodies. Although alterations in LRRK2 are commonly associated with PD, many of these patients do not exhibit a Lewy phenotype and it is thought that protein aggregration may be a disease modifier [9]. Methods to identify patients with an intrinsic aggregration phenotype may be useful in categorizing disease as well as in correctly targeting disease-modifying interventions to an appropriate class of patients. To explore the potential use of iPSC-derived neurons in detecting protein accumulation and aggregation phenotypes, we report the generation of iPSCs from a patient with a dominant autosomal form of PD caused by triplication of the *SNCA* locus. Dosage is known to influence disease progression with triplications causing earlier onset and more rapid progression than duplications of *SNCA* [10], [11], [12], [13]. Given their pronounced *in vivo* phenotype, *SNCA* triplications were selected to provide the highest probability of detecting disease-related phenotype that may be useful in categorizing disease characteristics in patient-derived neurons.

Familial autosomal dominant forms of PD have been documented in families with *SNCA* mutations or gene duplication/triplications as well as in families with mutations in proteins regulating the ubiquitin-proteasome pathway [11], [14], [15]. α-Synuclein protein aggregation and markers of cellular stress are also seen in other neurodegenerative disorders including dementia with Lewy bodies, pure autonomic failure, and multiple system atrophy [16]. Although overexpression of α-synuclein transgenes leads to protein aggregation in normal cells, the study of native processes leading to aggregation in affected individuals has been hindered by the inaccessibility of human neurons *in vivo*, the limitations inherent in studying postmortem samples from PD patients, and the inability to accurately recapitulate human disease in transgene models [12], [17]. Here we report that neurons derived from a SNCA triplication patient exhibit intrinsic protein accumulation and aggregation phenotypes.

## Results

### Patient Clinical Presentation

The subject of this study was a 48-year-old male with an *SNCA* triplication (Trpl-HDF). The patient presented with early onset, autosomal dominant, PD at age 38. The patient was left-handed and had been in excellent health most of his life. His initial symptoms in 2007 were fatigue, tremor and decreased dexterity. At that time, he was not experiencing any changes in speech, gait or cognition. He had undergone an MRI scan, which was unremarkable. He exhibited a resting and mild intention tremor, but his gait was normal. Thus, in 2007, the patient appeared to have mild Parkinson's disease and no medication was required.

At the time of the clinical examination in summer 2008, the patient noted mild problems with recent memory. He did not experience any psychiatric symptoms such as delusions or hallucinations but reported some anxiety and slight depression. He also reported urinary urgency and occasional constipation. He indicated that he had normal sleep patterns but in speaking with his wife he did exhibit nocturnal features suggesting REM behavioral disorder. He had undergone a sleep study and apparently obstructive sleep apnea was suspected, but did not tolerate continuous positive airway pressure. The patient stated that his sense of smell was poor for at least one year and the B-SIT smell test showed abnormal sense of smell with 6/12 points. From time to time he noted periodic blurring of vision and diplopia and had obtained new corrective lenses. He was also experiencing motor fluctuations on his current medication schedule. He stated that in the “on” state he is nearly normal, but continued to experience difficulty with his handwriting. When he is in the “off” state or when the medication wears out, his speech slowed, he drooled, his handwriting worsened, and he had more difficulty with dexterity such as dressing or hygienic activities. He had not noticed any difficulty with his balance, but reported that when his medication wore off he experienced hesitation on turning. Tremor also worsened when the medication wore off. In addition, the patient noted some upper back pain. He estimated about 80% of the day was in the “on” state and had not experienced any dyskinesia. There was a mild to moderate rest tremor of his left hand and a slight tremor of his right hand in addition to an action tremor of mild degree left greater than right. He was able to rise from the chair and walk with no difficulty. His stride was normal but there was axial rigidity and loss of arm swing bilaterally. A slight hesitation on turning when asked to multitask while he walked. There was no sign of ataxia on finger nose or heel shin testing and he did not exhibit dystonia or dyskinesia. Symptoms suggested typical progression of Parkinson's disease.

### Generation of iPSCs

**Figure 1:**
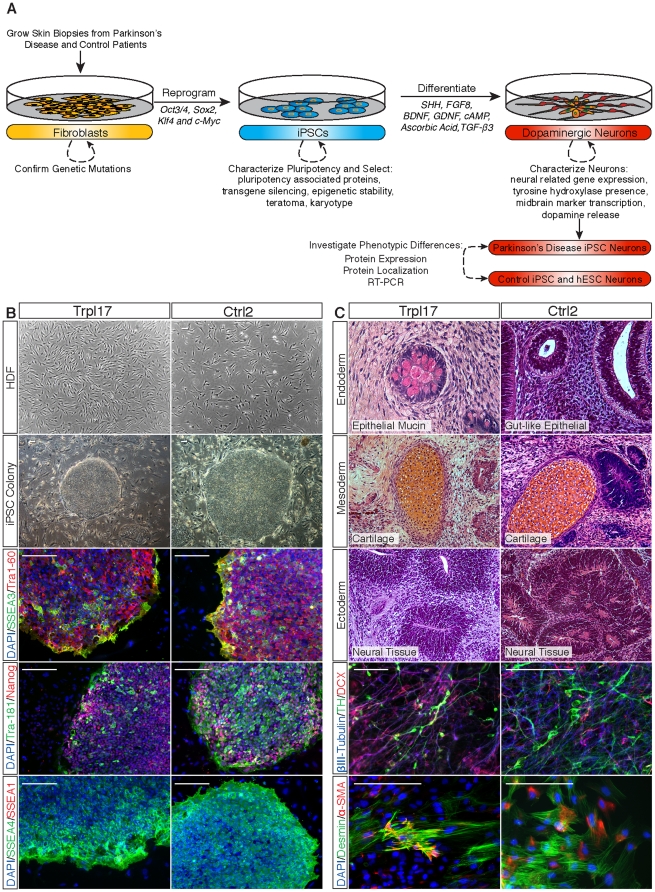
Analysis of pluripotency of iPSC lines. (**A**) Human dermal fibroblasts (HDF) from Trpl17 and Ctrl2 were reprogrammed into iPSCs capable of forming colonies on mouse embryonic fibroblasts (MEFs) that expressed pluripotency associated antigens, SSEA3, TRA1-60, TRA-181, NANOG, and SSEA4 and were negative for lineage marker SSEA1. (**B**) Pluripotency of iPSC lines Trpl17 and Ctrl2 was assayed through immunohistochemistry of *in vivo* teratoma formation, demonstrating formation of cell types from all three germ layers; epithelial tissue (endoderm), cartilage (mesoderm) and neuronal tissue (ectoderm). Pluripotency potential was further characterized through embryoid body differentiation *in vitro*. Immunofluorescent staining confirmed the presence of cell types indicative of all three germ layers, βIII-tubulin and TH (ectoderm), desmin and α-SMA (mesoderm) and a-fetoprotein (endoderm; **Figure S4A**).

A skin biopsy was taken and iPSCs were generated from primary human dermal fibroblasts (Trpl-HDF) and from HDFs isolated from his unaffected sister (Ctrl-HDF) with a normal *SNCA* copy number. Trpl-HDF and Ctrl-HDF were reprogrammed by induced expression of the exogenous transcription factors *OCT4*, *SOX2*, *KLF4*, and *c-MYC*, through retroviral gene insertion [18], [19], [20], [21], [22], and resulting iPSC clones were expanded and characterized for human embryonic stem cell (hESC)-like properties (Fig. 1A). Multiple iPSC clones from each donor line were similar to hESCs with respect to morphology, expansion, gene expression, epigenetic regulation, and teratoma formation. Representative hESC-like clones from each donor were selected for further analysis. All clones that were analyzed maintained hESC-like properties in culture for more than 30 passages on MEF feeders and in feeder free media and expressed pluripotency-associated antigens: alkaline phosphatase (AP), TRA1-60, TRA1-81, NANOG, SSEA-1, SSEA-3, and SSEA-4 (Fig. 1B and Fig. S1A). To ensure line stability, selected reprogrammed clones were further examined for karyotype and silencing of exogenous reprogramming factors. We observed that the karyotype was normal and that there was robust silencing of the exogenous retroviral-based reprogramming factors, as detected by RT-PCR, while expression of endogenous pluripotency transcription factors was comparable to levels in native H9 hESCs (Fig. S1B and C).

Other characteristics of the iPSCs also matched those of hESCs. The *NANOG* and *OCT4* promoters were hypermethylated in the untransduced donor fibroblasts and hypomethylated in the reprogrammed iPSCs and H9 hESCs (Fig. S2A). Imprinted genes maintained their differentially-methylated state in reprogrammed colonies, confirming iPSC epigenetic stability and suggesting reduced tumorigenic risk and complete reprogramming (Fig. S2B) [23], [24], [25]. Likewise, iPSC telomerase activity was comparable to hESCs and was significantly elevated compared to untransduced fibroblasts (Fig. S3) [26], [27]. Finally, when we tested the ability of iPSC lines to form cell types indicative of all three germ layers (mesoderm, ectoderm, and endoderm), we observed that the lines spontaneously differentiated into characteristic cells from each germ layer both *in vitro* and *in vivo* in teratoma assays (Figs. 1C and Fig. S4A and B).

### Neuronal differentiation

**Figure 2:**
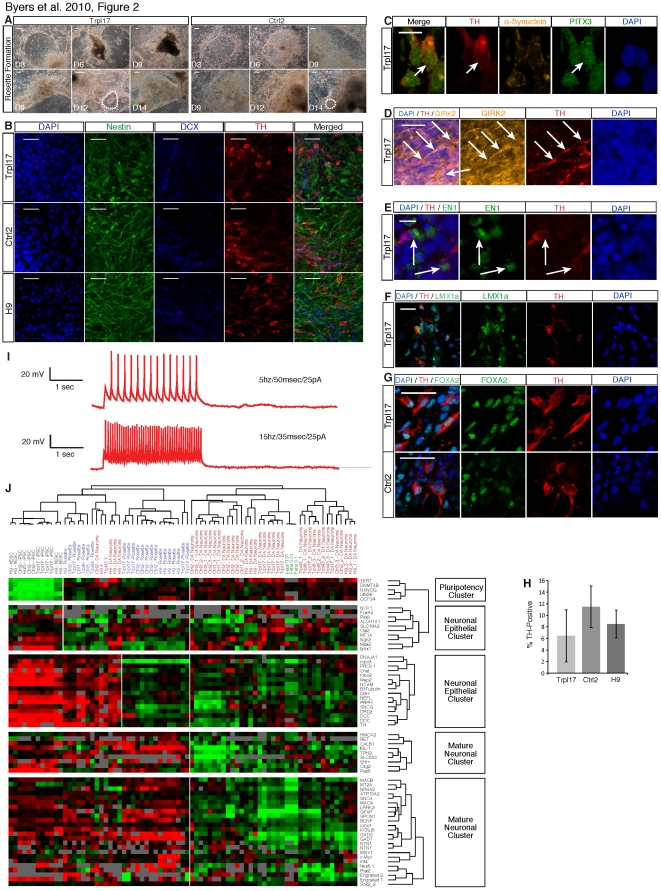
Characterization of directed dopaminergic neuron differentiation. (**A**) Trpl17 and Ctrl2 neural induction time course 5× phase contrast images at iso-location (except where noted): Days 3, 6, 9 in the upper row are images of the same location in each culture. However, rosettes formed on the expanding colony periphery and not at the initial site of colony adhesion. Thus, the lower row shows days 9, 12, and 14 at a different location in each culture. Formations of neural rosettes (example in white circle) were visible begging on day 9 and continued to expand and proliferate through day 14 and beyond. (**B**) IF analysis of pluripotent lines H9, Trpl17, and Ctrl2 for DAPI, Nestin, DCX and TH. The presence of significant numbers of TH positive neurons demonstrates the ability to differentiate and enrich for dopaminergic neurons. IF confocal micrograph demonstrating (**C**) midbrain specific transcription factor pitx3 colocalization with TH and *SNCA* in Trpl17 dopaminergic neurons; (**D**) midbrain associated ion channel GIRK2, (**E**) midbrain specific transcription factor EN1, and (**F**) midbrain associated transcription factor LMX1a were expressed in Trpl17 and Ctrl2 dopaminergic neurons. (**G**) FOXA2, a marker expressed by floor plate progenitors and their midbrain dopaminergic neuron derivatives, was expressed in Trpl17 and Ctrl2 dopaminergic neurons. (**H**) Single cell patch-clamp recordings demonstrate that neurons derived from Trpl17 iPSCs are electrophysiologically functional with multiple spike trains and a normal −70 mV resting potential. (**I**) Clustering of samples and genes in a 96×96 microfluidic qPCR array. Samples clustered by differentiation stage (undifferentiated, rosette and DA neurons) rather than by line (H9, Trpl8, Trpl17, Trpl43, Ctrl1, Ctrl2, or Ctrl3.). Fetal brain samples clustered with DA neurons rather than undifferentiated or rosettes cells. Five self-clustered gene sections, of the full 96 genes (**Figure S7**), were examined closely, demonstrating self-assembly of pluripotency, general neural, and mature neural related genes with expression patterns that correlate to sample developmental stage and human fetal brain samples. (**J**) The number of TH positive cells per well was not significantly different, although Trpl17 had a greater variance. Scale bars = 100 µm (a–b), 10 µm (c, e), 25 µm (d, f–g) and error bars = S.E.M.

To generate neurons, independently derived iPSC clones from the *SNCA* triplication patient (Trpl8, Trpl17 and Trpl43), the unaffected sibling (Ctrl1, Ctrl2 and Ctrl3) and hESCs (H9) were differentiated according to the method of Perrier and colleagues [28]. hESC-like colonies were mechanically isolated and sequentially exposed to recombinant proteins and growth factors known to be involved in the patterning of dopaminergic neurons *in vivo* (Fig. S5). Independent of cell line, differentiation *in vitro* followed a developmental sequence with cells first forming neural rosettes, or rings of neuroectodermal progenitors, which subsequently generated neurons. Neural rosettes were present from approximately day 9 of neural induction until passaging at day 28 (Fig. 2A). The neural progenitors were then passaged onto laminin-coated coverslips and patterned with SHH (Sonic Hedgehog) and FGF8 (Fibroblast Growth Factor 8) prior to terminal differentiation in the presence of TGF-β3 (transforming growth factor b-3) (Fig. S5).

Immunofluorescent (IF) staining was used to evaluate differentiated cultures for the presence of nestin, a type VI intermediate filament protein indicative of neural progenitor cells; doublecortin (DCX), a microtubule-associated protein found in immature neurons; and tyrosine hydroxylase (TH), the rate-limiting enzyme in dopamine biosynthesis, indicative of dopaminergic neurons. After 50 days of differentiation, the cultures contained nestin-positive neural progenitors, DCX-positive immature neurons and TH-positive mature DA neurons (Fig. 2B)

Neurons were often localized in islands of differentiating nestin-positive progenitor cells. These dense aggregates produced extensive axonal outgrowth (Fig. S6A) and TH-positive neurons were found within and around these differentiating clusters (Fig. S6A, B). Cells within the differentiating clusters were PAX6 positive, confirming their neuroectodermal origins (Fig. S6C). Cells were also positive for LMX1α (Fig. 2F) and FOXA2, markers that are expressed by floor plate progenitors and floor plate-derived midbrain dopaminergic neurons (Fig. 2G) [29], [30]. TH-immunoreactive neurons also expressed engrailed-1 (EN1), pituitary homeobox 3 (pitx3) and the midbrain associated ion channel G protein-activated inward rectifier potassium channel 2 (GIRK2) (Fig. 2C–E and Fig. S7).

Scoring of the relative abundance of TH-positive neurons indicated that there were no significant differences between iPSC or hESC lines (Fig. 2H). Combined, the markers confirm midbrain dopaminergic neuron development and indicate that the underlying genetic anomaly in the PD iPSC did not significantly impact the formation of these neurons in culture.

To further characterize the progression of events that accompanies differentiation, gene expression profiles were evaluated at different stages during the differentiation process using microfluidic, high-density quantitative PCR (qPCR). Samples of 500–1000 cells were manually collected from replicate cultures of undifferentiated pluripotent cells, cultures highly enriched for neural rosettes, and cultures enriched for mature neurons (days 0, 28 and 50, respectively). Cells were lysed and processed for expression analysis of a 96-gene panel diagnostic of pluripotent stem cells, endoderm, mesoderm, neural crest and CNS cells, including neurons and glia (Material S1). Relative CT values for expression were calculated as the difference between the gene of interest and the geometric mean of four housekeeping genes: *GAPDH*, *CTNNB1*, *EEF1a1*, and *CENTB3* per sample.

We used cluster analysis to examine gene expression and time points across the sample groups (Fig. 2J). We observed that samples self-clustered primarily by stage of differentiation and secondarily by iPSC line confirming that *SNCA* triplication and control lines were highly similar in both gene expression and differentiation patterns. Moreover, genes clustered into functional groups that correlated with developmental and cell-type specific groups, providing evidence of native differentiation cascades expected for normal neural development. The clustered group of undifferentiated samples from Trpl8, Trpl17, Trpl43 and Ctrl1, Ctrl2 and Ctrl3 iPSCs, as well as, H9 hESCs showed higher expression of the self-clustered group of pluripotency related genes, *FOXD3*, *NANOG*, *LIN28*, *OCT4*, *TERT* and *DNMT3B* relative to differentiated cultures (Fig. S8 and S9). A second cluster, containing general neuroectodermal markers, was upregulated in expression in samples that were enriched in rosettes and differentiated DA neurons relative to undifferentiated samples (Fig. S8). Finally, genes associated with fully developed DA neurons, including *SHH*, *BDNF*, *MAO-A* and *MAO-B*, were enriched in expression in samples of mature neurons, as compared to rosettes and undifferentiated cells (Fig. S8). As a control, human fetal brain mRNA samples were run in parallel during the qPCR assay and clustered most significantly with the neural progenitors and mature neurons present after 50 days of differentiation.

### Elevated SNCA expression

**Figure 3:**
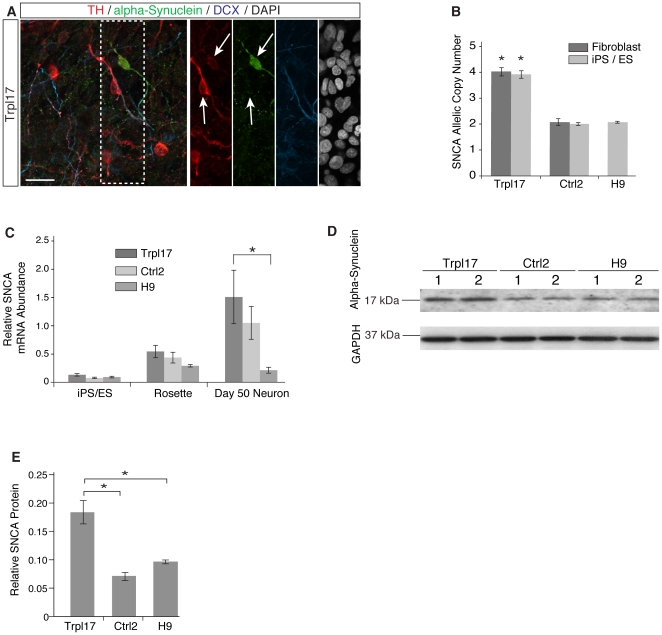
Characterization of midbrain DA neurons and phenotypic differences between pluripotent lines. (**A**) IF confocal micrographs of DAPI, α-synuclein, TH, and DCX, demonstrate variable α-synuclein accumulation in Trpl17 derived DA neurons. (**B**) *SNCA* gene copy number computation through qPCR of *SNCA* 3rd intron. Absolute copy number (2×2−ΔCT) is reported: Ctrl-HDF, Ctrl2, and H9 ES cells all had a normal diploid copy number. Trpl-HDF and Trpl17 possessed 4 allelic copies of *SNCA*, consistent with a single allele triplication. (**C**) *SNCA* mRNA expression analysis shows an ANOVA significant difference between all three lines, and a post-hoc significant *SNCA* expression difference between Trpl17 and H9 neurons. (**D**) Western blots show greater abundance of 17 kDa monomeric α-synuclein in Trpl17 DA cultures than control Ctrl2 and H9. Cells from two independently differentiated wells are shown per sample. (**E**) The ratio of monomeric α-synuclein/GAPDH was calculated for each sample and results are expressed as the mean of two samples. Scale bar = 50 µm. Error bars = SEM in **c** and **d** and sample range in **g**. *p<0.05 and **p<0.001.

*SNCA* triplication is associated with elevated expression and accumulation of α-synuclein. We noted that some SNCA triplication neurons displayed abnormally bright IF staining for α-synuclein protein, however, these neurons did not co-express TH (Fig. 3A and Fig. S9). Copy number analysis confirmed that Trpl17 HDFs and iPSCs possessed four copies of *SNCA*, compared to two copies of *SNCA* in H9 and Ctrl2 derived cells (Fig. 3B) and qRT-PCR showed mRNA abundance in neuronal cultures was 1.44 times higher in Trpl17 compared to Ctrl2 cultures and 7.17 times higher than hESC H9 cultures (the latter being statistically significant, p = 0.013) (Fig. 3C). α-synuclein accumulation was further quantified by Western analysis of protein lysates from Trpl17, Ctrl2 and H9 60-day differentiated neuron cultures. Although the western blot was unable to take into account expression heterogeneity between the cells of each culture, 17 kD monomeric α-synuclein was elevated approximately 2-fold in Trpl17 cultures compared to sibling Ctrl2 or hESC H9 derived neuronal cultures (Fig. 3D and E). These findings are consistent with blood and post-mortem tissue samples from *SNCA* triplication individuals, in which α-synuclein levels are higher relative to controls [31].

### Oxidative stress, protein aggregation-related genes and cell death

**Figure 4:**
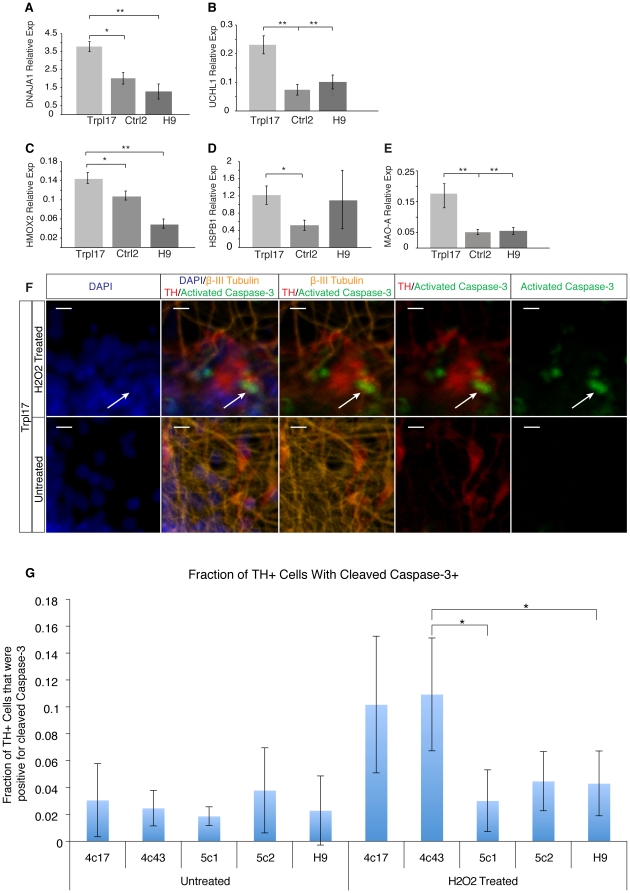
Analysis of oxidative stress levels and response to peroxide induced cell stress. (**A–E**) ANOVA significance analysis across Ctrl2, Trpl17 and H9 cultures revealed five differentially expressed stress related genes. Trpl17 neural cultures had the highest expression of each ANOVA identified differentially expressed stress related gene; DNAJA1, HMOX2, UCHL1, HSPB1 and MAO-A. (**F**) Confocal micrographs showing activated Caspase-3 in Trpl17 DA cultures with and without H2O2 treatment. (**G**) A plot of the fraction of TH+ cells that were activated Caspase-3 positive for each line with and without 200 µM H2O2 treatment. Error bars = S.E.M. and scale bars = 100 µm (j) and 10 µm (k). *p<0.05, **p<0.001.

To determine whether protein aggregation was accompanied by an increase in cell stress, the transcription level of 14 oxidative stress and protein aggregation-related genes (Material S1) was analyzed in DA neural cultures. Within the 14-gene panel, expression of DNAJA1, HMOX2, UCHL1, HSPB1 and MAO-A was found to be significantly different across Trpl17, Ctrl2 and H9 cultures in a three way ANOVA. For each of these genes, a post-hoc test showed 1.5- to 4-fold elevated expression in Trpl17 DA neural cultures compared to controls (Fig. 4A–E). HMOX2 is a known oxidative stress related protein, while DNAJA1 and HSPB1, a heat shock related protein, have been recognized as aggregate interacting proteins [32]. Mutations in UCHL1, a ubiquitin chaperone that participates in the normal removal of unwanted protein through the proteasomal pathway, can lead to familial forms of PD [7], while MAO has been implicated in hypothesized PD progression models by increasing the quantity of reactive oxygen species [33]. Related to the UCHL1 expression difference, a small number of ubiquitin positive cells were observed in the SNCA triplication neuronal cultures (Fig. S10A). The ability to isolate single midbrain dopaminergic neurons from these cultures would enable more detailed and sensitive analysis of PD associated phenotypes. However, the alterations in cell stress-related gene expression analyzed at the aggregated culture level and the presence of α-synuclein accumulation suggest that an *SNCA* triplication induces or increase the susceptibility of cell-intrinsic aberrant protein aggregation and stress in PD neurons in culture.

To determine whether cell death resulted from this increase in cell stress, we stained differentiated neurons for cleaved caspase-3 protein, a key regulator in cell apoptosis. DA neurons showed no significant differences in the abundance of caspase-3 positive cells under baseline conditions. However, when the antioxidant ascorbic acid was withdrawn from the culture media and cells were challenged with 200 µM hydrogen peroxide for 24-hours, significantly higher numbers of Caspase-3 positive TH neurons were observed in Trpl17 and Trpl43 compared to controls (Fig. 4F,G and Fig. S10B). Although this hydrogen peroxide screen does not take into account single cell variability of alpha-synuclein, it does demonstrate that cultures that accumulate alpha-synuclein are more vulnerable to oxidative stress induced cell death.

## Discussion

Parkinson's disease results from a complex interplay of genetics and environment. Not all patients exhibit identical features and future clinical approaches may benefit from diagnostic methods that predict disease mechanism and classify patients by virtue of neuron-intrinsic disease features. Here, we present data that indicates that differentiation of pluripotent stem cells may provide a cell-based model to detect cell autonomous disease processes based on protein deregulation/accumulation. The cell lines that we generated were derived from a PD patient with a naturally-occurring genomic triplication of the *SNCA* gene and his unaffected sibling. Importantly, the PD-affected line exhibited several disease-related phenotypes in culture: accumulation of α-synuclein, inherent overexpression of markers of oxidative stress, and sensitivity to peroxide induced oxidative stress. These findings show that the dominantly-acting PD mutation is intrinsically capable of perturbing normal cell function in culture and confirm that these features reflect, at least in part, a cell autonomous disease process that is independent of exposure to the entire complexity of the diseased brain. Importantly, this cell line contains wild type, non-mutant, α-synuclein, and thus is likely to reflect key characteristics relevant to the pathogenesis of the greater idiopathic PD patient population that display a similar protein aggregations. In idiopathic disease α-synuclein levels may be deregulated through epigenetics, environmental or a multifactorial process that result in similarly elevated α-synuclein and thereby initiate and propagates PD in a similar manner to the SNCA triplication neurons presented here.

Although the data presented here indicates that elevated wild-type α-synuclein is sufficient for cell-intrinsic vulnerability, the mechanism of pathogenic action is still unknown. The elevation of oxidative stress and aggregation of interacting proteins in the diseased line are consistent with free radical accumulation, perhaps as a result of protein aggregation-induced alterations in ubiquitin-proteasome function. Such perturbations in protein dynamics may influence the efficient removal or neutralization of oxidative molecules from the mitochondria's electron transport chain or tyrosine hydrolysis in dopaminergic neurons leading to increased neuron vulnerability. Although loss of midbrain dopaminergic neurons produces the prominent clinical features of PD, the degenerative process extends well beyond the midbrain dopaminergic neurons to affect autonomic function, cognition and mood. We confirm here that both TH-positive and TH-negative neurons display ubiquitinated intracellular inclusions. This is consistent with the dominant disease inheritance of the wild-type SNCA triplication with expression of SNCA in all neurons, and indicates that patient-derived iPSC can be used to study disease mechanisms and selective vulnerably across the many neuron subtypes involved in PD.

SNCA is not the only gene linked to hereditary PD. Mutations in LRRK2, UCHL1, PARKIN and others can lead to autosomal dominant inherited forms of PD, suggesting that the proteins encoded by these genes play an important role in neural cell homeostasis. In parallel work, we have shown that vulnerability and PD related phenotypes can be detected in iPSC-derived DA neurons, however, the apoptotic vulnerability was lower in the SNCA triplication lines than observed in DA neurons derived from a LRRK2-G2019S homozygous iPSC line [34]. Although both LRRK2 and SNCA mutations induce autosomal dominant forms of PD, the SNCA triplication line expresses higher levels of a normal protein while the LRRK2-G2019S missense mutation encodes a mutant protein, which in the homozygous state produces a more pronounced apoptotic vulnerability. In contrast, intracellular inclusions appeared to be more pronounced in differentiated neurons carrying the SNCA triplication.

Protein accumulation, oxidative stress marker increases and caspase activity in SNCA triplication iPSC-derived DA neurons demonstrates that PD linked mutations can produce detectable features of disease in young neurons, and we anticipate that the derivation of iPSC from both genetic and idiopathic patients will ultimately define whether a given mutation or more complex polygenic background confers a cell-autonomous vulnerability or whether the disease process in a given class of patients requires additional environmental or epigenetic contribution to confer selective vulnerability in dopaminergic neurons. The ability to observe disease-related phenotypes in PD iPSC-derived neurons across patient groups provides a useful platform for studying patient-specific molecular mechanisms of disease, environmental impacts and potential therapeutic applications.

## Materials and Methods

### Primary cell derivation and culture

Potential patients with specific genetic disease and healthy volunteers were informed of the project through poster advertising and referrals through patient advocacy and disease focus organizations (Parkinson's Institute). Written informed consent was obtained form all patients involved in the study. A Stanford institutional review board approved the primary cell line derivation (approval IRB-15028) and Stanford's stem cell research oversight committee (approval SCRO-212) approved all subsequent tissue use and experimentation. Potential donors were screened for men and women between the ages of 18 and 75 that had the genetically linked Parkinson's disease. After informed consent was obtained, primary human dermal fibroblasts (HDF) from the medial arm dermis of a 42-year-old male with SNCA triplication and his 46-year-old unaffected sister were obtained by first cleaning the region was cleaned with an alcohol swab and injecting 2–3 ml of 1% lidocaine with 1∶100,000 diluted epinephrine. Then, a 4 mm dermal specimen was removed with a core punch biopsy instrument and placed in sterile PBS, while the skin defect was closed with a 4-0 nylon suture and covered with a double antibiotic ointment bandage. Sutures were removed after 2 weeks. The skin tissue biopsy was washed in Ca^2+^ and Mg^2+^ free Dulbecco PBS (Invitrogen, Carlsbad, CA) and minced into small pieces before being seeded onto gelatin coated 6-well cell culture flasks (Corning, Acton, MA) containing DMEM/F12 supplemented with 100 IU/ml penicillin, 100 µg/ml streptomycin (Invitrogen), 10% FBS (DMEM/FBS culture media) and cultured at 37°C37C in 5% CO2. A minimal amount of culture media was used to promote tissue attachment to the gelatin-coated surface (1 ml of culture media per well). The media was brought up to 4 ml per well once the skin fragments attached and the media was changed every 2 days. Once fibroblasts began to migrate out, the attached biopsy fragments and any connected epithelial cells were removed and the fibroblasts were cultured to 80–90% confluence. This primary culture was passaged by brief exposure to 0.15% trypsin-EDTA (Invitrogen, Grand Island, NY) and seeded into four gelatin-coated 175 cm cell culture flasks with fresh DMEM-F12/FBS culture media. These somatic cells were cultured until they reached 90% confluence and subsequently frozen in DMEM/FBS culture media supplemented with 10% dimethyyl sulphoxide (DMSO, Sigma, St. Louis, MO) in aliquots of one million cells per cryovial. These somatic cells were thawed as required for reprogramming studies.

### Mouse embryonic fibroblast preparation

Mouse embryonic fibroblasts (MEFs) were prepared by sacrificing pregnant CF-1 mice (Charles River Laboratories), transferring fetuses to fresh PBS and rinsing until blood is absent. Animal work was approved under Stanford guidelines for animal safety and received approval from Stanford's administrative panel on laboratory animal care (approval APLAC-16146) and SCRO (approval SCRO-212) boards. Fetal heads and visceral organs were mechanically removed and the remaining carcass was rinsed in PBS, finishing in a rinse containing 5 ml Trypsin. Carcass tissue was cut into small pieces using scalpels, transferred from individual fetuses to a 15 ml centrifuge tube and incubated in 5% CO_2_ at 37°C37C for 20 min. 10 ml feeder medium was added to neutralize the Trypsin and the solution was pipetted up and down with a 25 ml stripper pipette. The sample was centrifuged at 1000 RPM for 5 min, the supernatant was aspirated and the sample was resuspended in 10 ml fresh feeder medium – repeated until solution was devoid of blood. Cells were plated at one fetus per T175 gelatin coated flasks with 30 ml feeder medium and incubated in 5% CO_2_ at 37°C.37C. Cells were subsequently passaged every 2–6 days (when flasks neared 90% confluency) for 5–7 passages, irradiated with 3000 rad gamma waves and then frozen down in DMSO.

### Retroviral production and infection

293FT cells were cultured in T175 flasks to ∼90% confluence on the day of transfection. For each 293FT T175 flask, two premixes were prepared; (1) 10 ug VSVG, 15 ug delta8.9, 10 ug retroviral vectors carrying Oct3/4, Sox2, Klf4 and c-Myc in 10 ml Opti-MEM; and (2) 120 ul Lipofectamine in 5 ml Opti-MEM. The premix solutions were incubated for 5 min at room temperature and then mixed gently by hand inversion. The resulting mix was then allowed to sit for 20 min at room temperature. The 293FT cells were treated with the resulting 15 ml mix and incubated in 5% CO_2_ at 37°C37C for 6 hours, after which the transfection mixture as replaced with 18 ml of 10% FBS in DMEM+Glutamax and incubated in 5% CO_2_ at 37°C37C for 72 hours. Harvest the supernatant in 50 ml conical tubes and spin down at 2000 rpm for 5 minutes. Filter the supernatant through Millex-HV 0.45 filter unit and store briefly for concentrating. To concentrate the virus, 30 ml fresh viral supernatant was concentrated 100× by centrifugation at 17,100 rpm for 2:20 hours at 20°C20C in a Beckman Coulter Optima L-80XP Ultracentrifuge and re-suspended in 300 ul of 10% FBS/DMEM. 100× viral stock stored at −80°C80C. Target fibroblasts were prepared at 1×10^5^ cells per well of a 6-well plate. The four prepared viral supernatants were mixed to the appropriate concentrations with fresh MEF medium and supplemented with 8 ng/mL polyprene and cultured with the cells overnight. The next day, cells were washed once with medium and incubated overnight with MEF medium to allow recovery from the infection. The retroviral infection was repeated for a total of two rounds.

### Reverse transcription, pre-amplification and RT-PCR

RNA was purified using QIAGEN Quick Prep-Mini kit or cell sorting directly into the pre-amplificaiton reaction mix. Samples were then reverse transcribed and pre-amplified with 5 ul CellsDirect 2× Reaction Mix, 10 ul Superscript III TR/Platinum Taq Mix (Invitrogen, CellsDirect One-Step qRT-PCR kit), 2.5 ul of 0.5× pooled primers and probes, 1.5 ul TE Buffer (QIAGEN) and 0.1 ul SUPERaseIn (Applied Biosystems). Rt-PCR was performed in 20 ul volumes with 10 ul ABI 2× Reaction Mix (Applied Biosystems), 1 ul FAM probe, 1 ul VIC probe, 0.5–2 ul of pre-amplified sample, fill remaining volume with water. Pre-amp thermocycle: 95°C95C for 10 min, 18 cycles of 95C for 15 seconds, 60C for 4 min, and then hold at 4°C.4C. Expression was normalized to the geometric mean of four housekeeping genes: GAPDH, CTNNB1, EEF1A1, CENTB3 (Centrin). Genes were then grouped into catagories representing developmental stages and cell types, with a fold change induction, normalized to the undifferentiated state, computed over the sample time points.

### Immunofluorescence and alkaline phosphatase staining

Alkaline phosphatase (AP) staining was performed with Vector® Red Alkaline Phosphatase Substrate Kit I (Vector Laboratories, CA), according to the manufacturer's protocol. For immunocytochemistry, cells were fixed in 4% paraformaldehyde/PBS for 10 minutes, washed twice with PBS, and blocked with 1–3% donkey, goat or chicken serum in PBS for 1 hour—all procedures were done at room temperature. For nuclear or intracellular staining, after fixation, the cells were permeabilized with 0.3% Triton-X100 for 30 minutes at room temperature. Subsequently, the primary antibodies were added to PBS and incubated for 1 hour at room temperature or overnight at 4°C. The cells were then washed with PBS before fluorescent-conjugated secondary antibodies were added and incubated for an hour at room temperature. Finally, the cells were rinsed with PBS three times and counterstained with DAPI. Stained samples were imaged directly on a LEICA inverted microscope or, if the samples were on coverslips, were mounted in PVA-DAVCO overnight and then imaged on an inverted Zeiss Confocal. Primary antibodies and their dilutions were as follows: Oct4 (diluted at 1∶100, Santa Cruz), Sox2 (1∶200, Millipore), SSEA1 (1∶200, Millipore), SSEA4 (1∶200, Millipore), TRA1-60 (1∶200, Millipore), TRA1-81 (1∶200, Millipore), Nanog (1∶100, Abcam), α-Fetoprotein (1∶200, Abcam), βIII-Tubulin (1∶200, Abcam), α-Smooth muscle actin (1∶200, Abcam), Vasa (1∶200, Abcam) Nestin (1∶200, Santa Cruz), Doublecortin (1∶200, Santa Cruz), Tyrosine hydroxylase (1∶500, Pel-Freez Biologicals), SNCA (1∶500 or 1∶1600, NeoMarkers), SNCA (1∶200, Thermo Scientific), SNCA (1∶200, Labvision), Ubiquitin (1∶250, Millipore), FoxA2 (1∶50, Santa Cruz). Secondary antibodies were raised in either donkey, goat or rabbit with conjugates: Alexa 488-conjugated anti-rabbit IgG (1∶500, Invitrogen), Alexa 594-conjugated anti-rabbit (1∶500, Invitrogen), Alexa 647-conjugated anti-rabbit (1∶500, Invitrogen), Alexa 488-conjugated anti-mouse IgM (1∶500, Invitrogen) and Alexa 488 anti-mouse IgG (1∶500, Invitrogen), FITC anti-Mouse conjugated, Cy3 anti-Rabbit, and Cy5 anti-Goat (1∶500 Jackson Immuno). In micrographs DAPI, FITC, Cy3, Cy5, are visualized as Blue, Green, Red, and Yellow (or Blue, when DAPI is removed).

### Bisulfite sequencing

To determine methylation regions, bisulfite sequencing was performed on genomic DNA isolated from hESCs and iPSCs, grown on feeder-free media, with Methyl Easy Xceed Rapid DNA Bisulfite Modification Kit (Human Genetic Signatures, Sydney, New South Wales, Australia) per manufacturers directions. The promoter regions of Oct3/4 and Nanog were amplified by PCR, as previously described [33]. PCR products were subcloned into pCR2.1 TOPO (Invitrogen), and twelve clones from each sample were analyzed by sequencing with M13 universal primer.

### Spectral karyotyping

To prepare metaphase spreads, 10 µl/mL colcemid was added to the cell culture and incubated for up to 2 hours. The growth medium was removed and collected and the cells were rinsed with HBSS. Cells were treated with 2 mL trypsin and incubated at 37°C for 5–7 min. The collected colcemid medium from the earlier step was re-applied to the cells to stop neutralize the trypsin and resuspend the cells. Resulting solution was centrifuged at 1000 RPM for 6 min and the supernatant was partially aspirated and resuspended in the native solution by flicking the tube. 5 drops of pre-warmed hypotonic solution were slowly added against the side, while flicking with a finger, until 1 ml had been added. Volume was then brought to 2 mL with hypotonic solution. The sample was incubated at 37°C for 7 min and then centrifuged at 1000 RPM for 6 min. Medium was added to resuspend the cells. To fix the cells, 5 drops of fixative were slowly added against the side of the tube and the volume was brought to 2 mL with fixative. Cells were gently mixed and fixed for 30 min at room temperature. After fixing, the sample was centrifuged, aspirated, and resuspended as above. Any clumps were removed by vacuum from the side of tube, and 2 ml of fixative were added to the tube. Sample was then mixed, incubated for 20 min at room temperature, centrifuged, aspirated, and resuspended in 2 ml of fixative. Samples were transferred onto pre-cleaned slides in ∼100 ul drops, left to dry overnight, and then analyzed on a SKY microscope (Spectral-Imaging, Vista, CA).

### SNCA Triplication Confirmation

SNCA gene copy number analysis: Genomic DNA was isolated from frozen cell pellets using a DNeasy Blood & Tissue Kit (Qiagen 69504). A FAM-MGB Taqman Copy Number Assay against the 3^rd^ intron of SNCA (Hs04791950_cn) and a VIC-TAMRA Taqman Copy Reference Assay against RNase P (#4403326) were used with Taqman Genotyping MasterMix (#4371353; Applied Biosystems) to co-amplify 20 ng of genomic DNA in quintuplicate from each sample on a Stratagene Mx3000P RT-PCR System. The absolute copy number of SNCA for each sample is reported by calculating the relative copy number (SNCA/RNase P) for each reaction (2^−ΔCT^) and then adjusting by a factor of 2 which reflects the normal allelic copy number of RNase P in the genome. 2 alleles of SNCA are normally present, a duplication of SNCA yields 3 alleles, and a triplication of SNCA yields 4 alleles.

### Exogenous and Endogenous Expression Analysis

Primer sequences used for determining exogenous and endogenous expression of the OCT4, SOX2, KLF4 and c-MYC. Exogenous: pMXs-AS3200: ttatcgtcgaccactgtgctgctg (Used as Reverse primer for all) OCT4- Forward: ccccagggccccattttggtacc. SOX2- Forward: ggcacccctggcatggctcttggctc. KLF4- Forward: acgatcgtggccccggaaaaggacc. cMYC-Forward: caacaaccgaaaatgcaccagccccag. Endogenous: OCT4-Reverse: cctagctcctcccctccccctgtc. (Use the above primers as Forward) SOX2-Reverse: cctcttttgcacccctcccatttccc. KLF4-Reverse: tgattgtagtgctttctggctgggctcc. cMYC-Foward: ttgaggggcatcgtcgcgggaggctg. cMYC-Reverse: cgagaggaccccgtggatgcagag.

### In-vitro differentiation

To investigate *in vitro* differentiation, cell culture medium was replaced with ∼80% KO DMEM, 20% fetal bovine serum (FBS), 1% 100× L-glutamine, 1% 100× BME, 1% 100× non-essential amino acids (NEAA), and 1% 100× penicillin-streptomycin. After culturing for the desired time period, cells were either stained for Immunocytochemistry or harvested for protein or RNA analysis.

### In-vivo teratoma formation and immunohistochemistry

As sated previously, animal work was approved under Stanford guidelines for animal safety and received approval from Stanford's administrative panel on laboratory animal care (approval APLAC-16146) and SCRO (approval SCRO-212). To determine iPSC potential to form all three germ layers *in vivo*, hESCs and iPSCs cells were harvested from 6-well or 10 cm plates through brief Collagenase IV treatment and transferred to 200 ul of hESC medium. The cells were either grafted subcutaneously behind the neck or in the hind limp muscles of female SCID mice (Charles River). After 8–10 weeks post-transplantation, grafts were dissected and fixed with 4% paraformaldehyde/PBS overnight. The tissues were then paraffin embedded, sectioned and stained with Masson's Trichrome, Mayer's Mucicarmine, Saffron O and Hematoxylin and Eosin.

### Directed midbrain dopaminergic differentiation

To direct differentiation of pluripotent cell colonies towards a midbrain dopaminergic (DA) neuron cell fate, a modified version of the protocol outlined by Perrier et al. was utilized^25^. Two or three hESC or iPSC colonies were mechanically harvested and gently dissected into 4–6 pieces per colony. Cells were then plated onto a 6 cm co-culture dish with irradiated MS-5 (xMS-5) stromal cells (MS-5 cells were expanded in MS-5 stromal cell culture medium: 455 ml a-MEM, 2 mM L-glutamine, 50 ml heat inactivated FBS, Pen-strep) and cultured for 16 days in Serum Replacement Media (15% KOSR in KO-DMEM, GIBCO), with media changes every two days. After 16 days, media was changed to N2+/+ (progesterone 20 nM, putrescine 100 uM, sodium selenite 30 nM, insulin 5 ug/ml, transferrin 0.1 mg/ml in DMEM-F12, GIBCO). At day 28, neural rosettes in the colonies were passaged through mechanical micro-dissection and transferred onto Poly/Laminin coated (15 ug/ml polyornithine, 1 ug/ml laminin, SIGMA) 6-well plates and cultured for one week in 200 ng/ml Sonic Hedgehog (SHH-C24II (a more active form than the unmodified protein), R&D Systems), 100 ng/ml Fibroblast Growth Factor 8 (FGF-8, R&D Systems), 20 ng/ml Brain Derived Neurotrophic Factor (BDNF, R&D Systems), and 0.2 mM Ascorbic Acid (AA, SIGMA) in N2+/+ media. On day 35, when cultures were approximately 80% confluent, colonies were passaged through digestion in Ca^2+^/Mg^2+^ free HBSS (GIBCO) at room temperature for 1 hour and subsequent mechanical dissociation. Removed cell aggregates were centrifuged at 200 g for 5 minutes, resuspended and plated on Poly/Laminin coated dishes at 50–100×10^3^ cells/cm^2^ in N2+/+ media with the previously noted, day 28, growth factors. On day 42, differentiation was induced for 8 days through growth factor withdrawal by changing media to N2+/+ with 20 ng/ml BDNF, 20 ng/ml Glial Derived Neurotrophic Factor (GDNF, R&D Systems), 1 ng/ml Transforming Growth Factor B3 (TGF-B3, R&D Systems), 1 mM dibutyryl Cyclic Adenosine MonoPhosphate (cAMP, SIGMA) and 0.2 mM AA in N2+/+ media. On day 50, cell cultures were harvested for analysis.

### HPLC Dopamine Release:

After 60 days of iPSC patterning and differentiation into dopamine neurons, neurons were depolarized to evoke dopamine release. Media was removed and 1 ml of N2 media supplemented with 56 mM KCl was added per well (6 well dish) and incubated at 37°C for 15 minutes. The medium was collected and immediately frozen in liquid nitrogen and stored at −80°C until assay. Protein lysates were used to estimate relative cell number in each well. Lysates were made in ice cold 25 mM Tris supplemented with a Complete Mini protease inhibitor cocktail tablet (Roche). Lysates were sonicated and cleared by maximum speed centrifugation in a tabletop microcentrifuge. Soluble protein concentrations were measured by a standard Bradford assay. To measure dopamine, media samples were thawed, stabilized at a final concentration of 0.4 N perchloric acid, and centrifuged at 15,000 rpm at 4°C for 12 minutes to clear debries. The supernatant was then collected and dopamine was assayed by HPLC with electrochemical detection (Coularray detector, ESA, Chelmsford, MA) using a reverse phase C18 column (Perkin Elmer Instruments, Shelton, CT). The mobile phase consisted of a mixture of 90 mM sodium acetate, 35 mM citric acid, 130 uM ethylenediaminetetraaceticacid (EDTA), 230 uM 1-octanesulfonic acid and 10% (v/v) methanol, with a flow rate of 1 mL/min. DA concentration was quantified by comparison of AUC to known standard dilutions. Dopamine release was normalized to protein determinations of cleared protein lysates.

### Electrophysiology

IPSC-derived cells cultured on glass coverslips were accessed via whole-cell patch clamp and assayed for their ability to generate action potentials in current clamp (CC). Electrophysiological recordings were obtained in Tyrode media ([mM] 150 NaCl, 4 KCl, 2 MgCl2, 2 MgCl2, 10 D-glucose, 10 HEPES, pH 7.35 with NaOH) using a standard internal solution ([mM] 130 KGluconate, 10 KCl, 10 HEPES, 10 EGTA, 2 MgCl2, pH 7.3 with KOH) in 3–5 MΩ glass pulled pipettes. Square pulse current steps of varying sizes and frequencies were injected to patched cells held at resting potential. Cells were chosen for patching based on either 1) their proximity or apparent extension of neuronal processes or 2) morphological similarity to typical neurons.

### α-Synuclein Western Blotting

Western blotting was performed to determine α-synuclein in iPSC-derived DA neuron cultures possessing an SNCA triplication. 20 ug of protein lysates harvested from Trpl17, Ctrl2 and H9 cell derived neuron cultures (60 DIV, 2 independent wells per sample) were subjected to SDS-PAGE and stained for α-synuclein (Thermo Scientific), stripped and re-stained for GAPDH (SIGMA). Monomeric α-synuclein was observed at approximately 17 kDa. Band intensities were measured using Image J, and ratio of monomeric α-synuclein/GAPDH was calculated for each sample. Results were reported as the means of two samples with error bars representing SEM. A one-way ANOVA with Duncan's post-hoc analysis found that Trpl17 neuronal cultures (possessing 4 genomic copies of *SNCA*) expressed roughly twice as much monomeric α-synuclein as Ctrl2 and H9 neuronal cultures (possessing the normal 2 genomic copies of *SNCA*).

### Statistical analysis

Student's t-test was used for comparing two samples with adequate sample numbers. For determining statistical significance between more than two groups, a one-way ANOVA was used with either Duncan's or Bonferroni's post-hoc analysis. Data was analyzed either in InStat Version 3.0a, Microsoft Excel v12.20 or MATLAB 2008.

## Supporting Information

Figure S1 **Characteristics of iPSC lines.** (A) Expression of pluripotency related markers alkaline phosphatase (AP), TRA-160, SSEA3, Nanog, TRA-181, SSEA4 and the absence of SSEA1 are shown for SNCA triplication iPSC clones Trpl8 and Trpl43. Stability of reprogrammed lines was assessed through (B) Spectral SKY karyotype analysis of iPSC lines. Shown here are representative karyotypes of Trpl17 and Ctrl2 iPSC line metaphase spreads. 20 replicates for each line showed no translocations, triplications, or deletions. (Replicates not shown). (C) Relative copy count from quantitative PCR analysis of endogenous and exogenous OCT3/4, SOX2, Klf4, and cMYC transcription factor expression in untransduced fibroblasts (Trpl-HDF and Ctrl-HDF), different pluripotent iPSC lines derived from Trpl-HDF (Trpl17 and Nrml46) and Ctrl-HDF (Ctrl2 and Ctrl3), pluripotent hESC line H9, and control HeLa RNA.
(PDF)

Figure S2 **Epigenetic status of pluripotency and imprinted genes.** (A) Methylation status of NANOG and OCT4 promoters as determined by bisulfide sequencing in untransduced fibroblasts (Trpl-HDF and Nrml-HDF) and pluripotent iPSC lines (Trpl17 and Nrml2). (B) Differential methylated regions on paternal, KCNQ1OT1, and maternal, H19, imprinting sites display balanced allelic methylation without hypo or hypermethlyation, suggesting the maintenance of a stable epigenetic state through reprogramming.
(TIF)

Figure S3 **Pluripotency related telomerase activity.** Relative telomerase activity in: untransduced HDF lines (Trpl2-HDF and Ctrl2-HDF); pluripotent iPSC lines Trpl17, Trpl46, Ctrl2 and Nrml3; hESC line HSF8; differentiated iPSC lines 4c17 and Ctrl2; negative buffer only control; and a TSR8 positive control. Heat inactivation used as an internal control. *p<0.01.
(TIF)

Figure S4 **Differentiation of iPSC lines.** (A) Confocal micrographs of α-Fetoprotein protein is detected by antibody recognition in EB spontaneous differentiation in iPSC lines Trpl17 and Ctrl2, indicative of endoderm lineage. Scale bars = 100 µm. (B) Further characterization of iPSC pluripotency. iPSC lines Trpl17 and Ctrl2 are capable of forming embryoid bodies in ultra-low adhesion plates and, when allowed to adhere, these embryoid bodies are able to spontaneously form beating cardiomyocytes. Scale bars = 200 µm.
(TIF)

Figure S5 **Schematic of DA neuron differentiation from pluripotent stem cells.** Protocol requires 50 days of differentiation and two passaging steps. Working concentrations for growth factors are: Sonic Hedgehog (SHH)-(Cys-24 modified) = 200 ng/ml, FGF8 = 100 ng/ml, Brain-Derived Neurtrophic Factor (BDNF) = 20 ng/ml, Glial Derived Neurtrophic Factor (GDNF) = 10–20 ng/ml, Transforming Growth Factor-B3 (TGF β3) = 1 ng/ml, Dibutyl Cyclic Adenosine Monophosphate (Dibutyl cAMP) = 0.5–1.0 mM, Ascorbic Acid = 0.2 mM.
(TIF)

Figure S6 **Neuronal differentiation.** (A) Day 50 DA neuron differentiation culture phase contrast micrographs of Trpl17, Nrml2 and H9. (B) Day 50 DA neuron differentiation IF confocal 20× micrograph showing a 423 µm TH+ axon extending from a DA neuron. (C) Trpl17 derived neurons express PAX6 in culture with TH and Nestin. Scale bars represent 200 µm (a) and 100 µm (b,c).
(TIF)

Figure S7 **Full heatmap display of Fluidigm qPCR results.** Gene list is filtered for those with ≥30% samples containing valid CT counts. Genes and samples were complete linkage clustered with uncentered correlation similarity metric using Cluster. Results were visualized in TreeView. The set was analyzed for correllation groups with known biochemical similarities. Gene categories labeled are discussed in Fig. 3.
(TIF)

Figure S8 **Transcript analysis of midbrain dopaminergic neuron differentiation over 50 days for pluripotent lines Trpl17, Ctrl2 and H9.** Pluripotency markers Oct4, c-Myc and TERT are turn off at the neural stem cell stage (NSC) and remain silenced in DA neurons. Notably, DNMT3B (a denovo DNA methyltransferase) shows moderate silencing at the NSC stage, while expression returns upon further differentiation to DA neurons. Neural associated markers NCAM, TH, MAOA, MAOB and SNCA show higher expression as the differentiation proceeds towards DA neurons. The relative ratio of Robo2, Robo1 and DDC (axon guidance associated proteins) is shown as a fraction of their total expression between the three stages of differentiation - Trpl17 derived neurons appear to express a higher fraction of Robo1 against Robo2 and DDC than the control lines.
(TIF)

Figure S9 **IF confocal micrographs for Trpl17 derived neurons.** Colocalization of SNCA and TH immunoreacivity, suggesting that DA neurons can express alpha-synuclein.
(TIF)

Figure S10 **(A) IF shows immunoreactivity colocalization of Ubiquitin and TH when Ubiquitin was expressed at a low level.** However, cells demonstrating strong Ubiquitin accumulation immunoreactivity were not positive for TH. (B) Confocal micrographs showing activated Caspase-3 in Trpl17 DA cultures with and without H2O2 treatment. Scale bars = 10 µm (a,b) and 100 µm (d). Error bars = s.e.m.
(TIF)

Material S1 **Patient clinical report and additional methods are described.**
(DOCX)
