# Supplementary material for: SNCA Triplication Parkinson's Patient's iPSC-derived DA Neurons Accumulate α-Synuclein and Are Susceptible to Oxidative Stress
Source: PLoS One. 2011 Nov 16;6(11):e26159. doi: 10.1371/journal.pone.0026159 (PMC3217921; doi:10.1371/journal.pone.0026159)
Supplement: Figure S1 — Characteristics of iPSC lines. (A) Expression of pluripotency related markers alkaline phosphatase (AP), TRA-160, SSEA3, Nanog, TRA-181, SSEA4 and the absence of SSEA1 are shown for SNCA triplication iPSC clones Trpl8 and Trpl43. Stability of reprogrammed lines was assessed through (B) Spectral SKY karyotype analysis of iPSC lines. Shown here are representative karyotypes of Trpl17 and Ctrl2 iPSC line metaphase spreads. 20 replicates for each line showed no translocations, triplications, or deletions. (Replicates not shown). (C) Relative copy count from quantitative PCR analysis of endogenous and exogenous OCT3/4, SOX2, Klf4, and cMYC transcription factor expression in untransduced fibroblasts (Trpl-HDF and Ctrl-HDF), different pluripotent iPSC lines derived from Trpl-HDF (Trpl17 and Nrml46) and Ctrl-HDF (Ctrl2 and Ctrl3), pluripotent hESC line H9, and control HeLa RNA. (PDF) [file pone.0026159.s001.pdf]

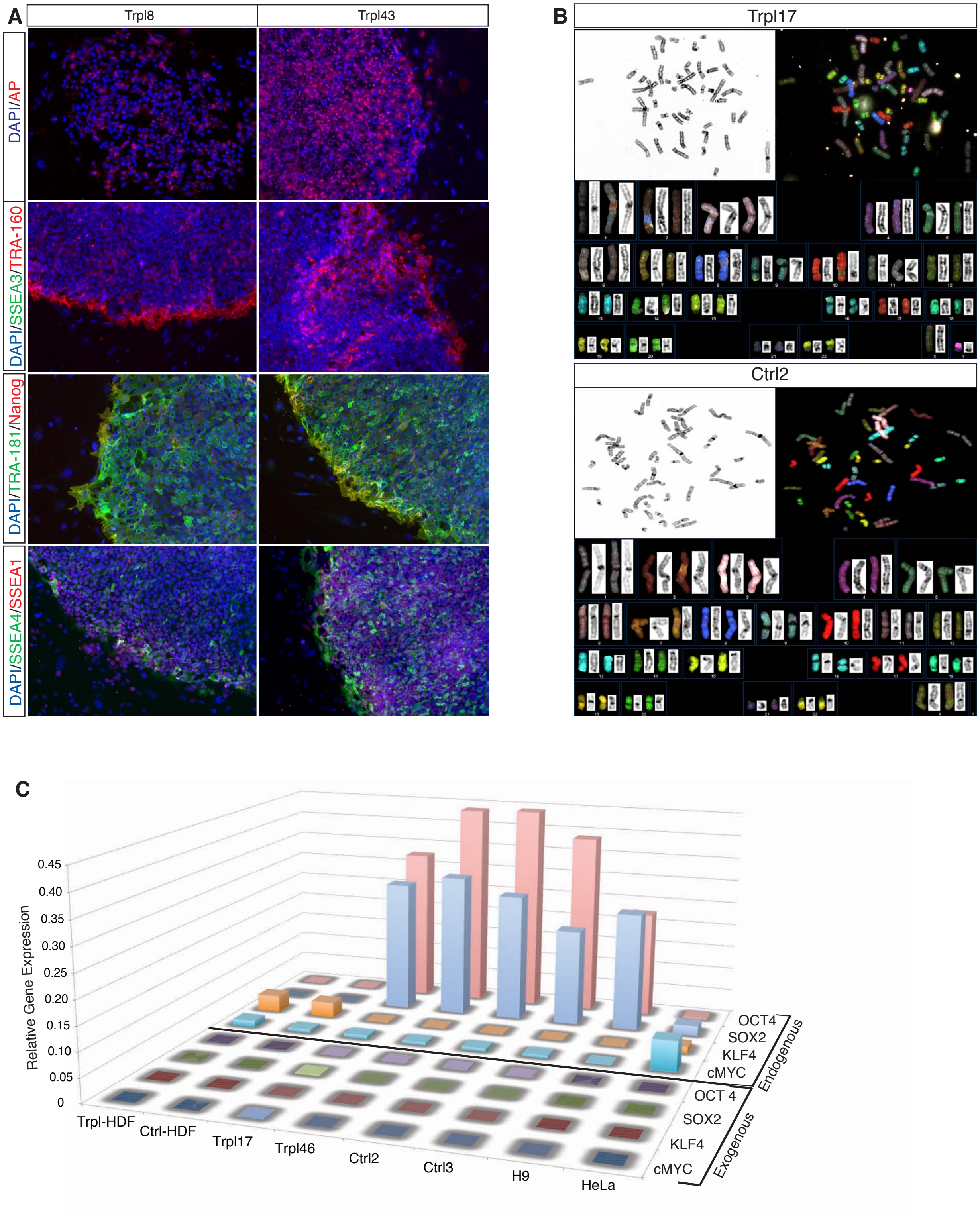

Supplementary Figure 1 Characteristics of iPSC lines. (**A**) Expression of pluripotency related markers alkaline phosphatase (AP), TRA-160, SSEA3, Nanog, TRA-181, SSEA4 and the absence of SSEA1 are shown for SNCA triplication iPSC clones Trpl8 and Trpl43. Stability of reprogrammed lines was assessed through (**B**) Spectral SKY karyotype analysis of iPSC lines. Shown here are representative karyotypes of Trpl17 and Ctrl2 iPSC line metaphase spreads. 20 replicates for each line showed no translocations, triplications, or deletions. (Replicates not shown). (**C**) Relative copy count from quantitative PCR analysis of endogenous and exogenous OCT3/4, SOX2, Klf4, and cMYC transcription factor expression in untransduced fibroblasts (Trpl-HDF and Ctrl-HDF), different pluripotent iPSC lines derived from Trpl-HDF (Trpl17 and Nrml46) and Ctrl-HDF (Ctrl2 and Ctrl3), pluripotent hESC line H9, and control HeLa RNA.
