# Supplementary figures and images for: SNCA Triplication Parkinson's Patient's iPSC-derived DA Neurons Accumulate α-Synuclein and Are Susceptible to Oxidative Stress
Source: PLoS One. 2011 Nov 16;6(11):e26159. doi: 10.1371/journal.pone.0026159 (PMC3217921; doi:10.1371/journal.pone.0026159)

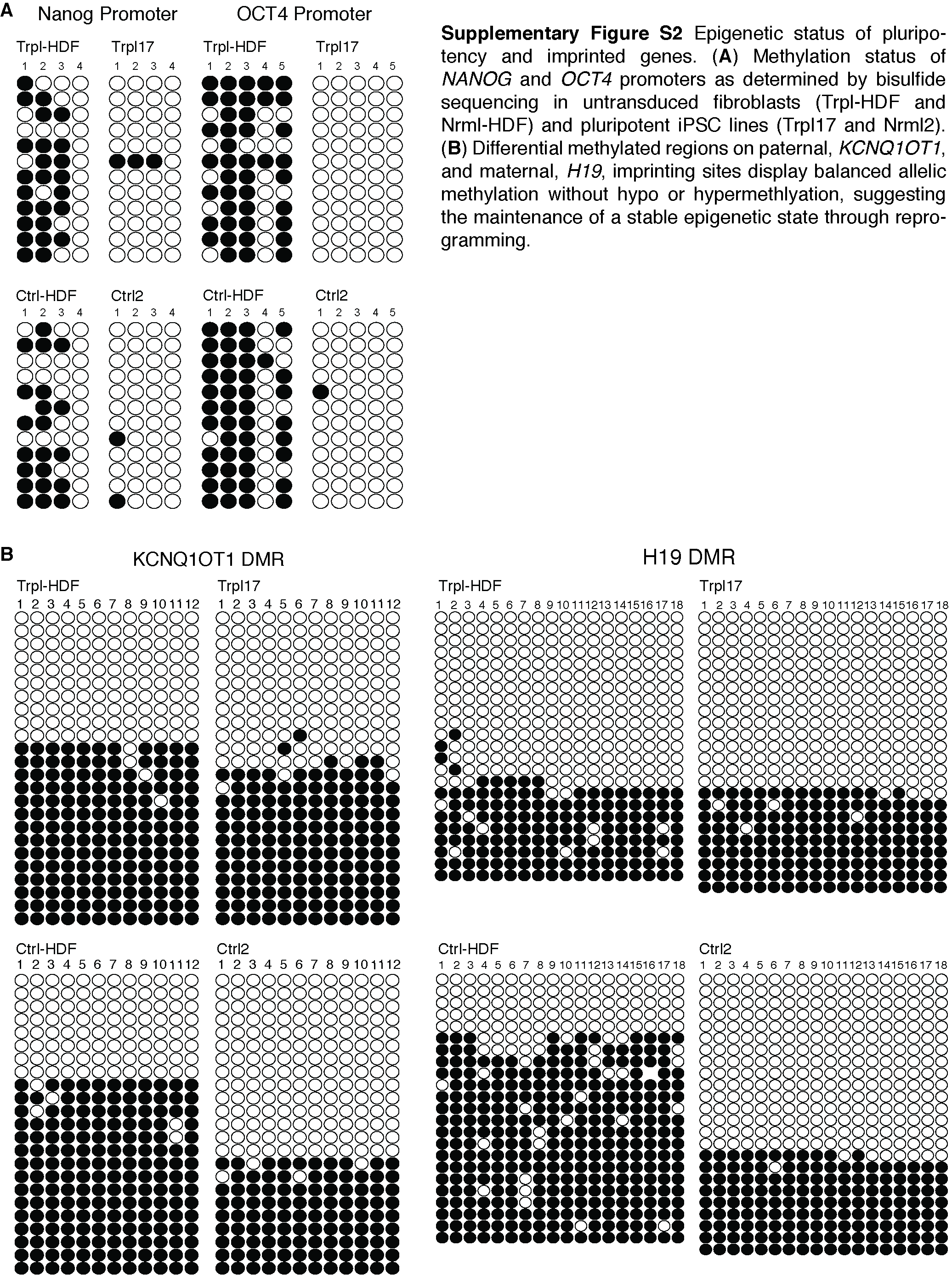

Supplement: Figure S2 — Epigenetic status of pluripotency and imprinted genes. (A) Methylation status of NANOG and OCT4 promoters as determined by bisulfide sequencing in untransduced fibroblasts (Trpl-HDF and Nrml-HDF) and pluripotent iPSC lines (Trpl17 and Nrml2). (B) Differential methylated regions on paternal, KCNQ1OT1, and maternal, H19, imprinting sites display balanced allelic methylation without hypo or hypermethlyation, suggesting the maintenance of a stable epigenetic state through reprogramming. (TIF) [file pone.0026159.s002.tif]

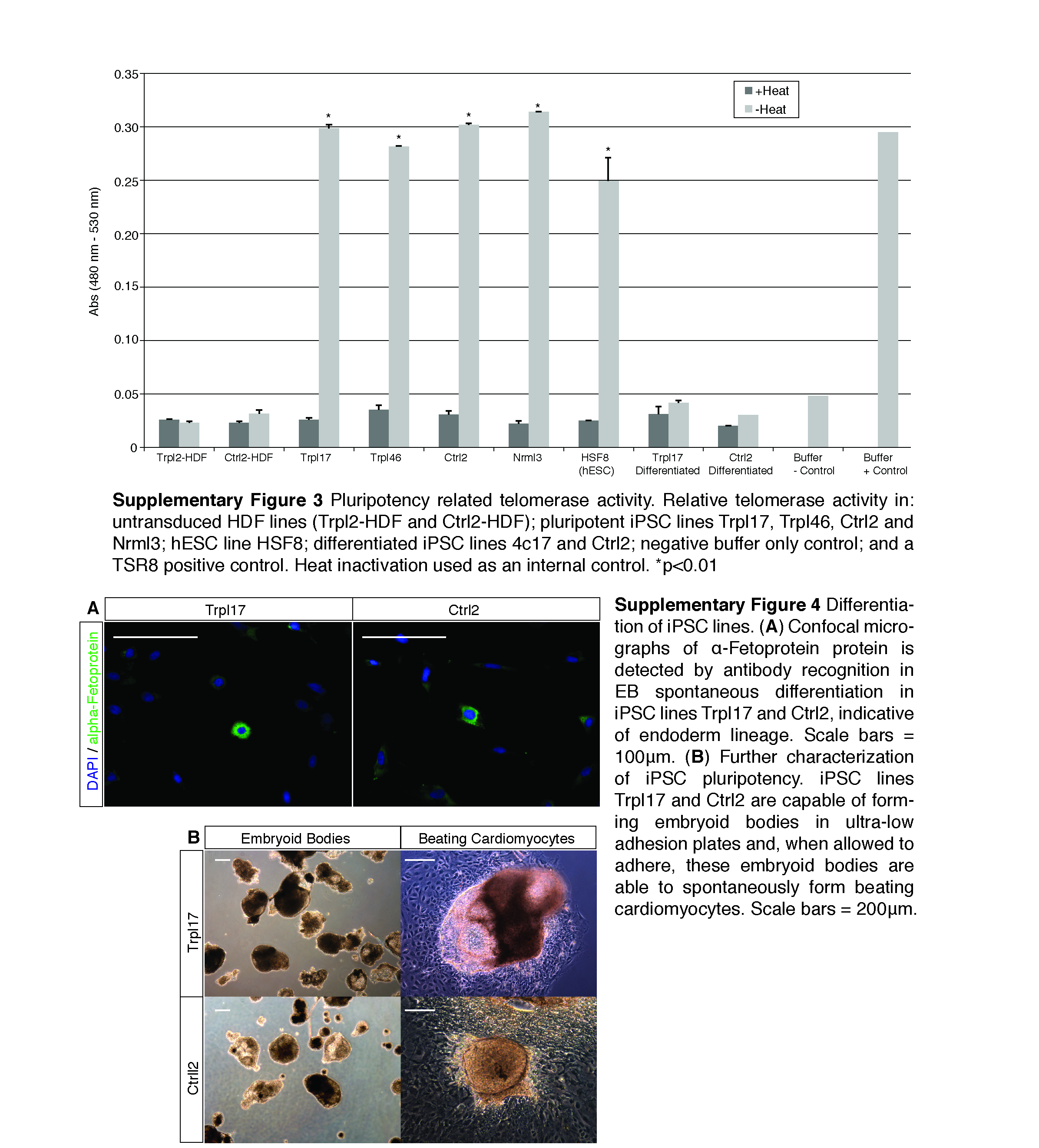

Supplement: Figure S3 — Pluripotency related telomerase activity. Relative telomerase activity in: untransduced HDF lines (Trpl2-HDF and Ctrl2-HDF); pluripotent iPSC lines Trpl17, Trpl46, Ctrl2 and Nrml3; hESC line HSF8; differentiated iPSC lines 4c17 and Ctrl2; negative buffer only control; and a TSR8 positive control. Heat inactivation used as an internal control. *p<0.01. (TIF) [file pone.0026159.s003.tif]

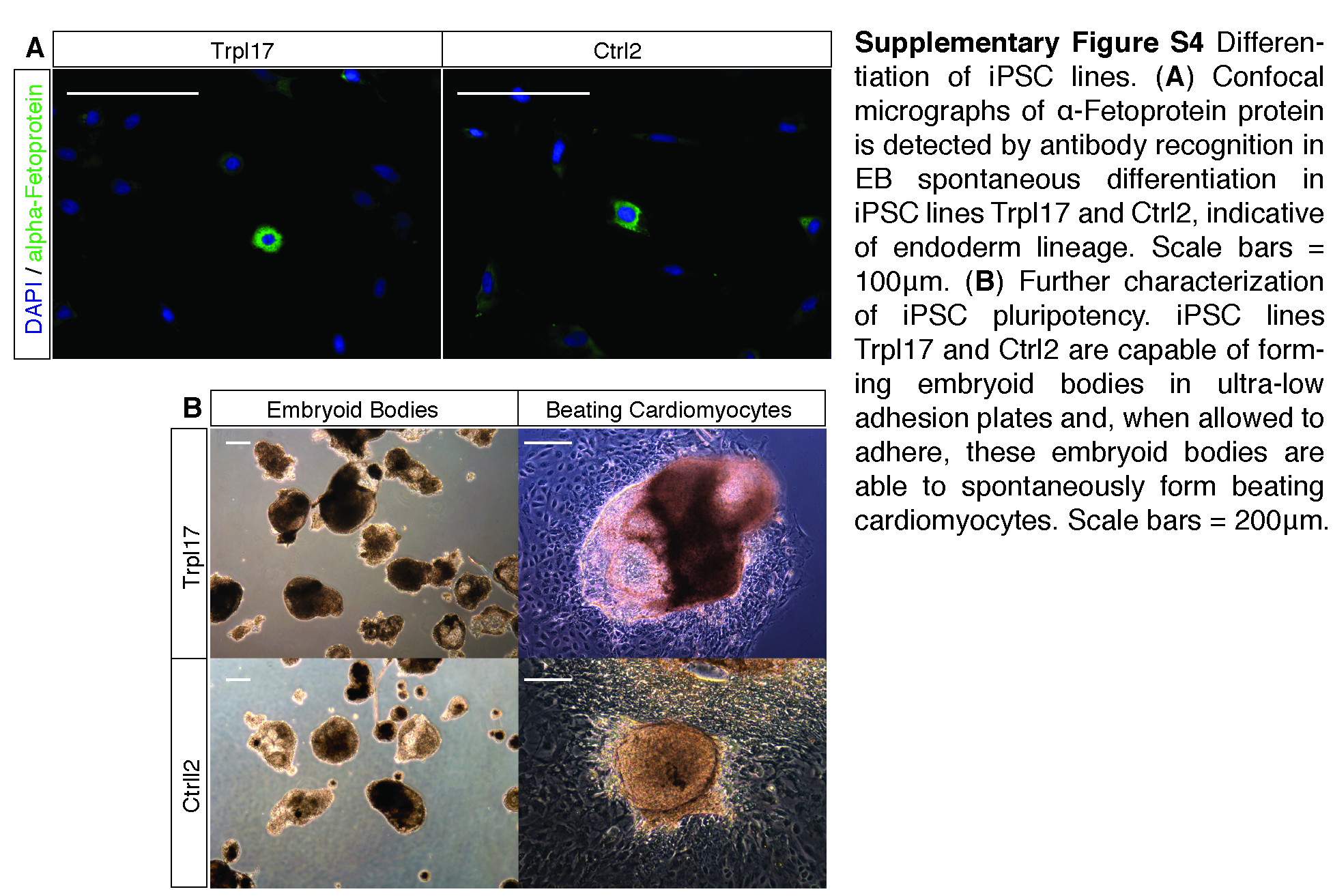

Supplement: Figure S4 — Differentiation of iPSC lines. (A) Confocal micrographs of α-Fetoprotein protein is detected by antibody recognition in EB spontaneous differentiation in iPSC lines Trpl17 and Ctrl2, indicative of endoderm lineage. Scale bars = 100 µm. (B) Further characterization of iPSC pluripotency. iPSC lines Trpl17 and Ctrl2 are capable of forming embryoid bodies in ultra-low adhesion plates and, when allowed to adhere, these embryoid bodies are able to spontaneously form beating cardiomyocytes. Scale bars = 200 µm. (TIF) [file pone.0026159.s004.tif]

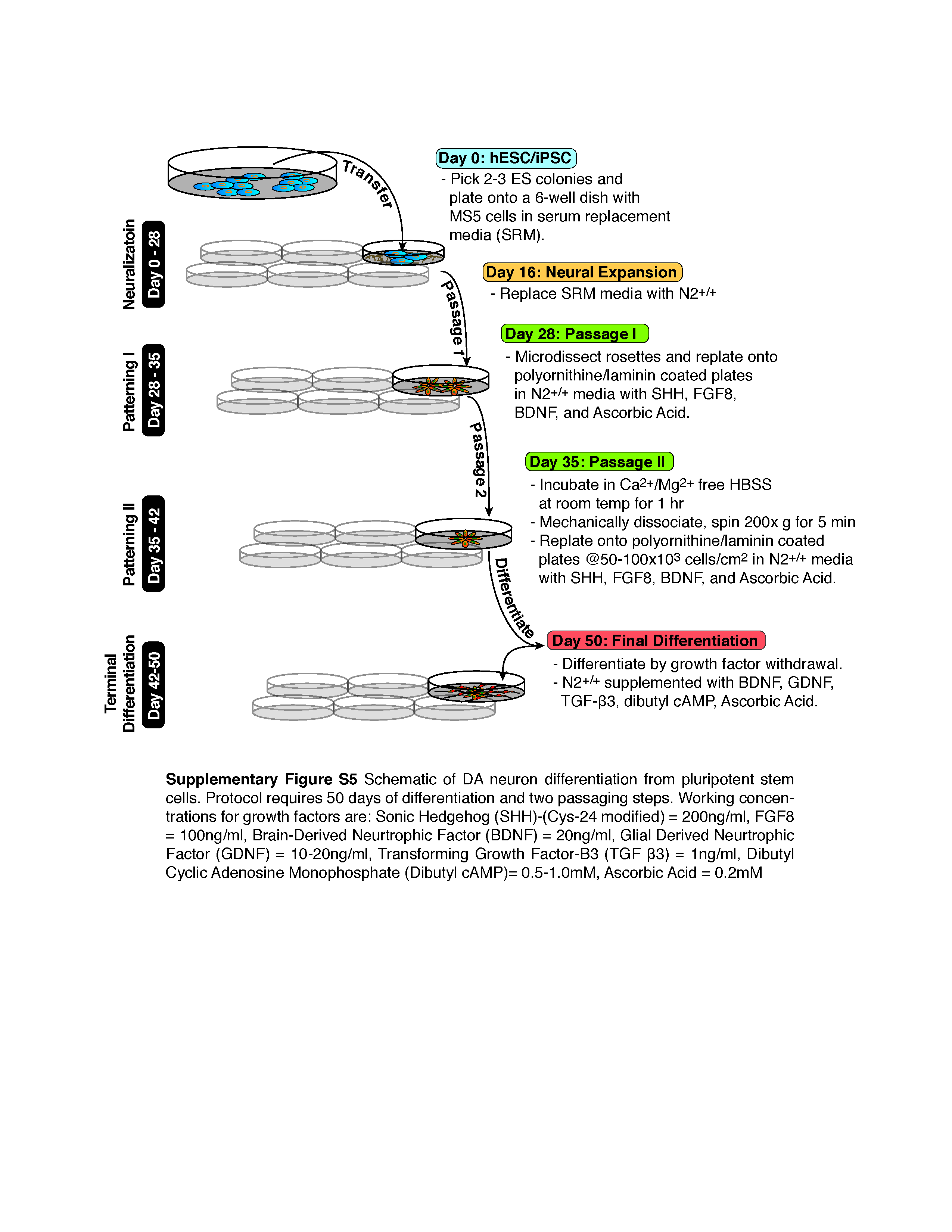

Supplement: Figure S5 — Schematic of DA neuron differentiation from pluripotent stem cells. Protocol requires 50 days of differentiation and two passaging steps. Working concentrations for growth factors are: Sonic Hedgehog (SHH)-(Cys-24 modified) = 200 ng/ml, FGF8 = 100 ng/ml, Brain-Derived Neurtrophic Factor (BDNF) = 20 ng/ml, Glial Derived Neurtrophic Factor (GDNF) = 10–20 ng/ml, Transforming Growth Factor-B3 (TGF β3) = 1 ng/ml, Dibutyl Cyclic Adenosine Monophosphate (Dibutyl cAMP) = 0.5–1.0 mM, Ascorbic Acid = 0.2 mM. (TIF) [file pone.0026159.s005.tif]

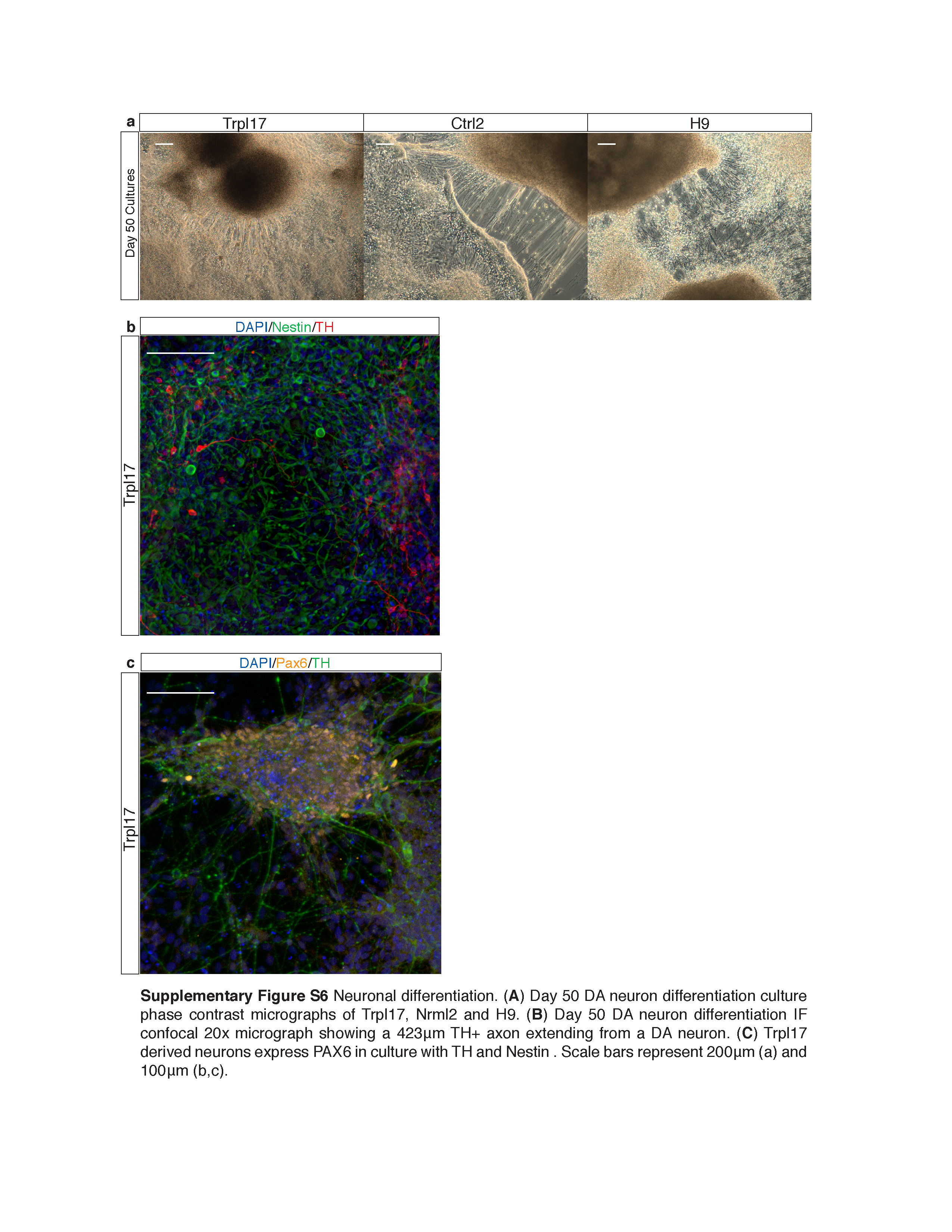

Supplement: Figure S6 — Neuronal differentiation. (A) Day 50 DA neuron differentiation culture phase contrast micrographs of Trpl17, Nrml2 and H9. (B) Day 50 DA neuron differentiation IF confocal 20× micrograph showing a 423 µm TH+ axon extending from a DA neuron. (C) Trpl17 derived neurons express PAX6 in culture with TH and Nestin. Scale bars represent 200 µm (a) and 100 µm (b,c). (TIF) [file pone.0026159.s006.tif]

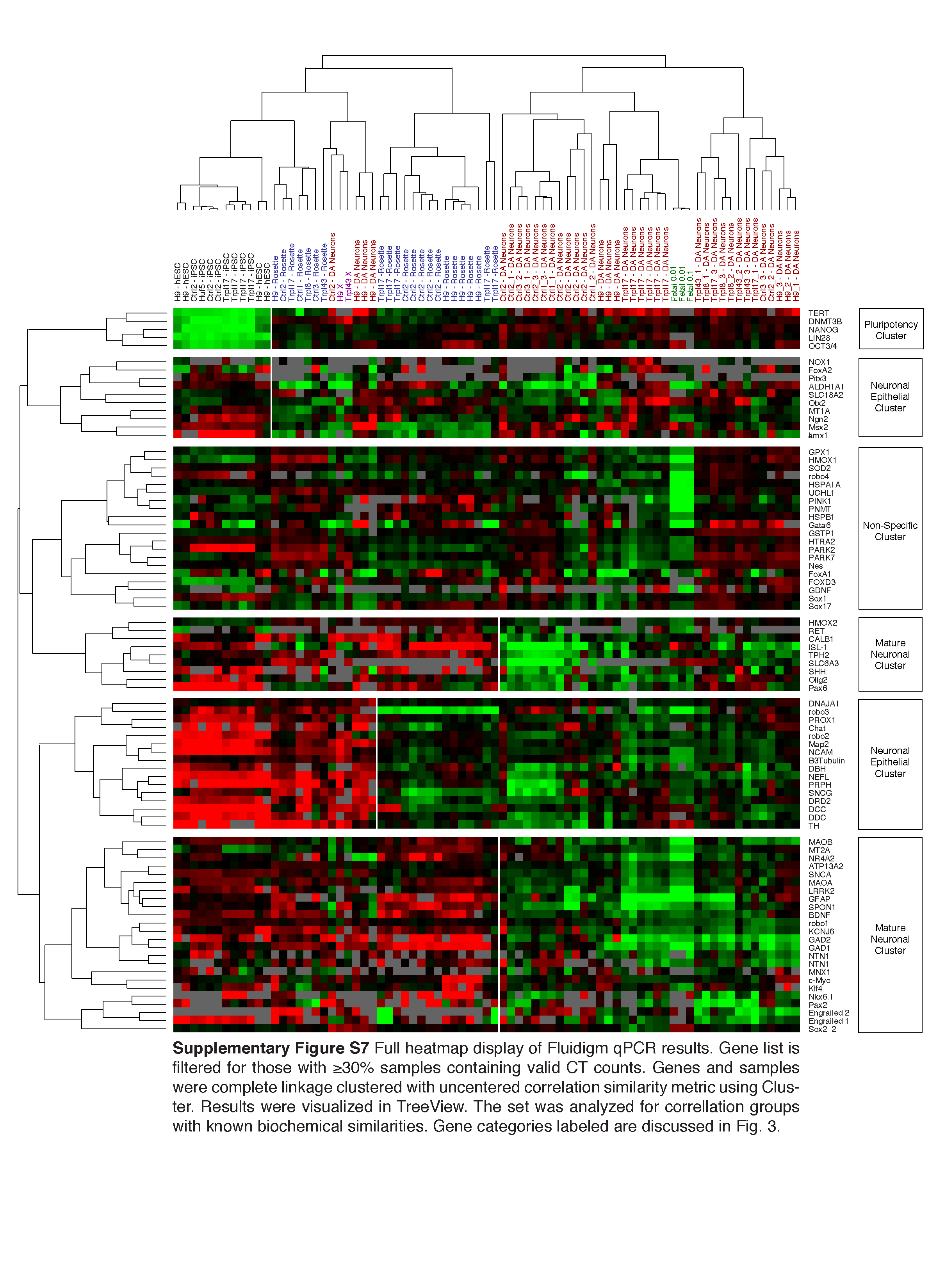

Supplement: Figure S7 — Full heatmap display of Fluidigm qPCR results. Gene list is filtered for those with ≥30% samples containing valid CT counts. Genes and samples were complete linkage clustered with uncentered correlation similarity metric using Cluster. Results were visualized in TreeView. The set was analyzed for correllation groups with known biochemical similarities. Gene categories labeled are discussed in Fig. 3. (TIF) [file pone.0026159.s007.tif]

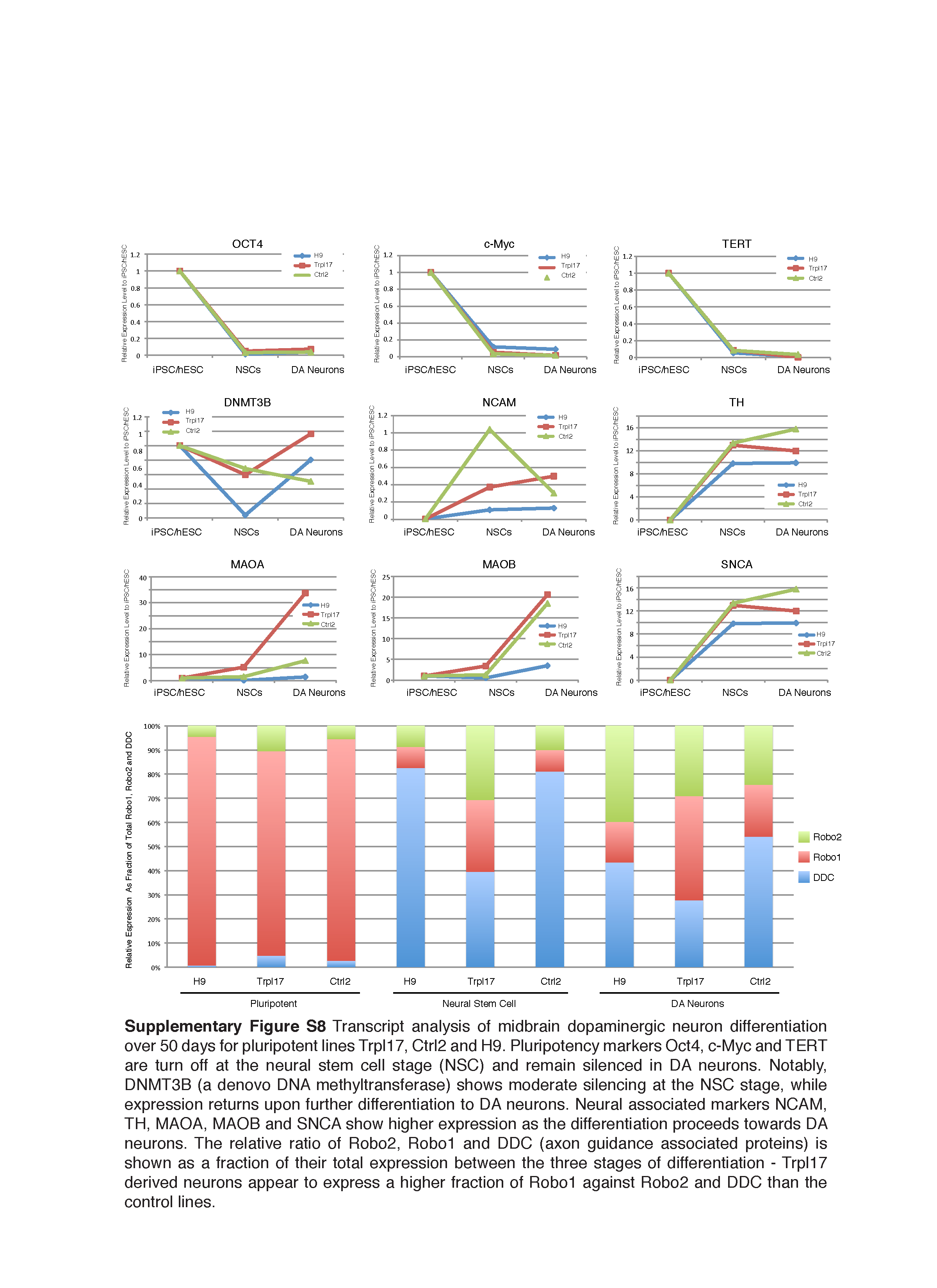

Supplement: Figure S8 — Transcript analysis of midbrain dopaminergic neuron differentiation over 50 days for pluripotent lines Trpl17, Ctrl2 and H9. Pluripotency markers Oct4, c-Myc and TERT are turn off at the neural stem cell stage (NSC) and remain silenced in DA neurons. Notably, DNMT3B (a denovo DNA methyltransferase) shows moderate silencing at the NSC stage, while expression returns upon further differentiation to DA neurons. Neural associated markers NCAM, TH, MAOA, MAOB and SNCA show higher expression as the differentiation proceeds towards DA neurons. The relative ratio of Robo2, Robo1 and DDC (axon guidance associated proteins) is shown as a fraction of their total expression between the three stages of differentiation - Trpl17 derived neurons appear to express a higher fraction of Robo1 against Robo2 and DDC than the control lines. (TIF) [file pone.0026159.s008.tif]

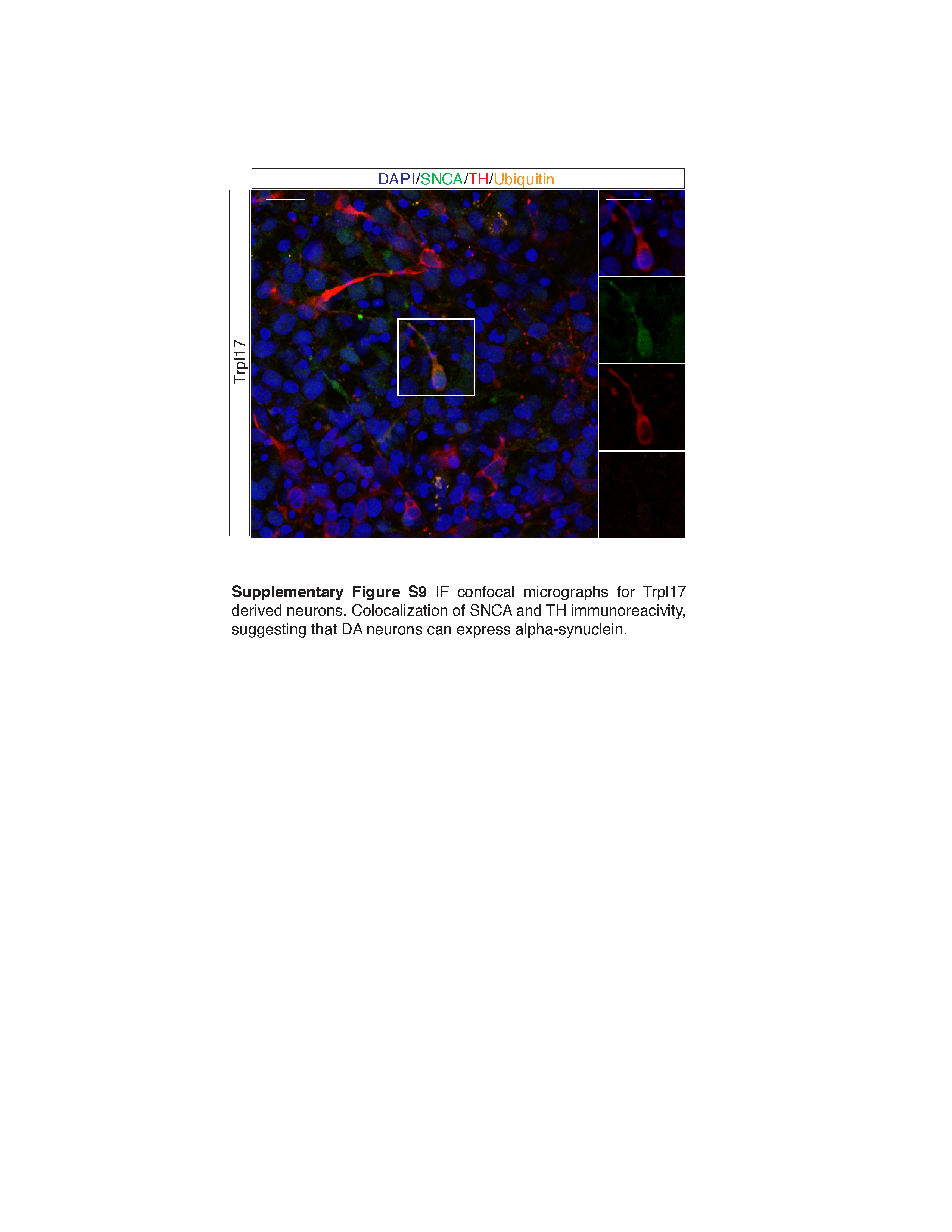

Supplement: Figure S9 — IF confocal micrographs for Trpl17 derived neurons. Colocalization of SNCA and TH immunoreacivity, suggesting that DA neurons can express alpha-synuclein. (TIF) [file pone.0026159.s009.tif]

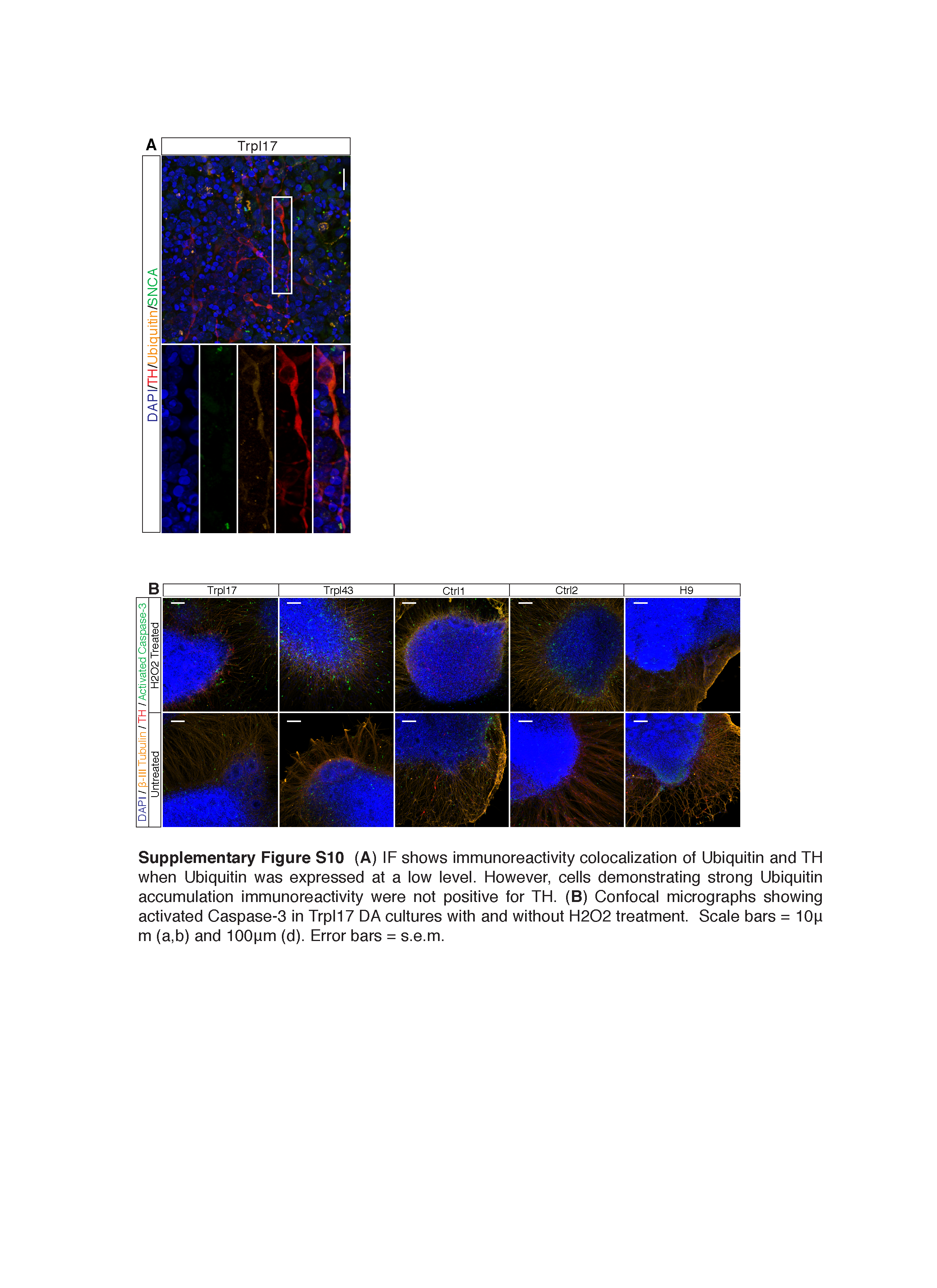

Supplement: Figure S10 — (A) IF shows immunoreactivity colocalization of Ubiquitin and TH when Ubiquitin was expressed at a low level. However, cells demonstrating strong Ubiquitin accumulation immunoreactivity were not positive for TH. (B) Confocal micrographs showing activated Caspase-3 in Trpl17 DA cultures with and without H2O2 treatment. Scale bars = 10 µm (a,b) and 100 µm (d). Error bars = s.e.m. (TIF) [file pone.0026159.s010.tif]
