# Supplementary material for: SNCA Triplication Parkinson's Patient's iPSC-derived DA Neurons Accumulate α-Synuclein and Are Susceptible to Oxidative Stress
Source: PLoS One. 2011 Nov 16;6(11):e26159. doi: 10.1371/journal.pone.0026159 (PMC3217921; doi:10.1371/journal.pone.0026159)
Supplement: Material S1 — Patient clinical report and additional methods are described. (DOCX) [file pone.0026159.s011.docx]

**PATIENT CLINICAL REPORT:**

The patient was 42-year-old at the time of the skin biopsy (Trpl-HDF). He was left-handed and had been in excellent health most of his life. His initial symptoms in 2007 were fatigue, tremor and decreased dexterity. At that time, he was not experiencing any changes in speech, gait or mentation. He had undergone an MRI scan, which was unremarkable. He exhibited a resting and mild intention tremor, but his gait was normal. Thus, he appeared to have mild Parkinson's disease, but his symptoms were mild and no medication was required.

At the time of the clinical examination in summer 2008, he noted mild problems with recent memory. He did not experience any psychiatric symptoms such as delusions or hallucinations but feels somewhat anxious and slightly depressed. He had urinary urgency and occasional constipation. He indicated that he had a normal sleep but in speaking with his wife he did exhibit nocturnal features suggesting REM behavioral disorder. He had undergone a sleep study and apparently obstructive sleep apnea was suspected, but did not tolerate CPAP. He stated that his sense of smell has been poor for at least one year and the B-SIT smell test showed abnormal sense of smell with 6/12 points. From time to time he had noted periodic blurring of vision and diplopia and had obtained new corrective lenses which apparently have improved his vision. He was also experiencing motor fluctuations on his current medication schedule. He stated that in the "on" state he is nearly normal, but continues to experience difficulty with his handwriting. When he is in the "off" state or when the medication wears out, his speech slows, he drools, his handwriting worsens, and he has more difficulty with dexterity such as dressing or hygienic activities. He had not noticed any difficulty with his balance, but he feels that when his medication wears out he experiences hesitation on turning. Tremor also worsens when the medication wears out. In addition, he noted some upper back pain. He estimated about 80% of the day is in the "on" state. He had not experienced any dyskinesia.

Mini Mental State examination scored 30/30. Vital signs sitting blood pressure 133/87, pulse rate 90, standing 110/73, pulse rate 90. Visual fields were full. Extraocular movements were abnormal with limited up and downgaze. Horizontal and vertical saccades were hypometric. There was a mild loss of facial expression. His speech was mildly soft and hoarse. His tongue moved somewhat slowly and there was a mild tremor when he extended his tongue.His reflexes were 2+ and symmetrical throughout. No pathological reflexes were noted. There was moderate rigidity in all four limbs and neck with some cogwheeling bilaterally. There was a mild to moderate rest tremor of his left hand and a slight tremor of his right hand in addition to an action tremor of mild degree left greater than right. He was able to rise from the chair and walk with no difficulty. His stride was normal but there was axial rigidity and loss of arm swing bilaterally. A slight hesitation on turning when asked to multitask while he walked. There was no sign of ataxia on finger nose or heel shin testing. His gait did not appear to be ataxic. No dyskinesia was noted. No dystonia was noted.

**SUPPLEMENTARY METHODS**

**Primary Cell Derivation and Culture**

Potential patients with specific genetic disease and healthy volunteers were informed of the project through poster advertising and referrals through patient advocacy and disease focus organizations (Parkinson’s Institute). Potential donors were screened for men and women between the ages of 18 and 75 that had the genetically linked Parkinson’s disease. After informed consent was obtained, primary Human dermal fibroblasts (HDF) from the medial arm dermus of a 42-year-old male and a 46-year-old female were obtained by first cleaning the region was cleaned with an alcohol swab and injecting 2-3 ml of 1% lidocaine with 1:100,000 diluted epinephrine. Then, a 4 mm dermal specimin was removed with a core punch biopsy instrument and placed in sterile PBS, while the skin defect was closed with a 4-0 nylon suture and covered with a double antibiotic ointment bandage. Sutures were removed after 2 weeks. The skin tissue biopsy was washed in Ca^2+^ and Mg^2+^ free Dulbecco PBS (Invitrogen, Carlsbad, CA) and minced into small pieces before being seeded onto gelatin coated 6-well cell culture flasks (Corning, Acton, MA) containing DMEM/F12 supplemented with 100 IU/ml penicillin, 100 μg/ml streptomycin (Invitrogen), 10% FBS (DMEM/FBS culture media) and cultured at 37C in 5% CO2. A minimal amount of culture media was used to promote tissue attachment to the gelatin-coated surface (1ml of culture media per well). The media was brought up to 4ml per well once the skin fragments attached and the media was changed every 2 days. Once fibroblasts began to migrate out, the attached biopsy fragments and any connected epithelial cells were removed and the fibroblasts were cultured to 80-90% confluence. This primary culture was passaged through brief exposure to 0.15% trypsin-EDTA (Invitrogen, Grand Island, NY) and seeded into four gelatin-coated 175cm cell culture flasks with fresh DMEM-F12/FBS culture media. These somatic cells were cultured until they reached 90% confluence and subsequently frozen in DMEM/FBS culture media supplemented with 10% dimethyyl sulphoxide (DMSO, Sigma, St. Louis, MO) in aliquots of one million cells per cryovial. These somatic cells were thawed as required for reprogramming studies.

**Mouse Embryonic Fibroblast Preparation**

Mouse Embryonic Fibroblasts (MEFs) were prepared by sacrificing pregnant CF-1 mice (Charles River Laboratories), transferring fetuses to fresh PBS and repeating until blood is absent. The fetal heads and visceral organs were mechanically removed and the remaining carcass was transferred between PBS dishes until blood was removed, finishing in a dish containing 5ml Trypsin. Carcass tissue was cut into small pieces using scalpels, transferred from individual fetuses to a 15ml centrifuge tube and incubated in 5% CO_2_ at 37C for 20 min. 10ml feeder medium was added to neutralize the Trypsin and the solution was pipetted up and down with a 25ml stripper pipette. The sample was centrifuged at 1000 RPM for 5 min, the supernatant was aspirated and the sample was resuspended in 10ml fresh feeder medium – repeated until solution was devoid of blood. Cells were plated at one fetus per T175 gelatin coated flasks with 30ml feeder medium and incubated in 5% CO_2_ at 37C. Cells were subsequently passaged every 2-6 days (when flasks neared 90% confluency) for 5-7 passages, irradiated with 3000 rad gamma waves and then frozen down in DMSO.

**Retroviral Production**

293FT cells were cultured in T175 flasks to ~90% confluence on the day of transfection. For each 293FT T175 flask, two premixes were prepared; (1) 10ug VSVG, 15ug delta8.9, 10ug retroviral vectors carrying Oct3/4, Sox2, Klf4 and c-Myc in 10ml Opti-MEM; and (2) 120ul Lipofectamine in 5ml Opti-MEM. The two premix solutions were incubated for 5 min at room temperature and then mixed gently by hand inversion. The resulting mix was then allowed to sit for 20 min at room temperature. The 293FT cells were treated with the resulting 15 ml mix and incubated in 5% CO_2_ at 37C for 6 hours, after which the transfection mixture as replaced with 18ml of 10% FBS in DMEM + Glutamax and incubated in 5% CO_2_ at 37C for 72 hours. Harvest the supernatant in 50ml conical tubes and spin down at 2000 rpm for 5 minutes. Filter the supernatant through Millex-HV 0.45 filter unit and store briefly for concentrating. To concentrate the virus, 30ml fresh viral supernatant was concentrated 100X by centrifugation at 17,100 rpm for 2:20 hours at 20C in a Beckman Coulter Optima L-80XP Ultracentrifuge and re-suspended in 300ul of 10% FBS/DMEM. 100X viral stock stored at -80C.

**Retroviral Infection**

Target fibroblasts were prepared at 1 x 10^5^ cells per well of a 6-well plate. The four prepared viral supernatants were mixed to the appropriate concentrations with fresh MEF medium and supplemented with 8ng/mL polyprene and cultured with the cells overnight. The next day, cells were washed once with medium and incubated overnight with MEF medium to allow recovery from the infection. The retroviral infection was repeated for a total of two rounds.

**Reverse Transcription, Pre-Amplification and RT-PCR**

RNA was purified using QIAGEN Quick Prep-Mini kit or cell sorting directly into the pre-amplificaiton reaction mix. Samples were then reverse transcribed and pre-amplified with 5ul CellsDirect 2x Reaction Mix, 10ul Superscript III TR/Platinum Taq Mix (Invitrogen, CellsDirect One-Step qRT-PCR kit), 2.5ul of 0.5x pooled primers and probes, 1.5ul TE Buffer (QIAGEN) and 0.1ul SUPERaseIn (Applied Biosystems). Rt-PCR was performed in 20ul volumes with 10ul ABI 2x Reaction Mix (Applied Biosystems), 1ul FAM probe, 1ul VIC probe, 0.5-2ul of pre-amplified sample, fill remaining volume with water. Pre-amp thermocycle: 95C for 10 min, 18 cycles of 95C for 15 seconds, 60C for 4 min, and then hold at 4C. Expression was normalized to the geometric mean of four housekeeping genes: GAPDH, CTNNB1, EEF1A1, CENTB3 (Centrin). Genes were then grouped into catagories representing developmental stages and cell types, with a fold change induction, normalized to the undifferentiated state, computed over the sample time points.

**Bisulfite Sequencing**

To determine methylation regions, bisulfite sequencing was performed on genomic DNA isolated from hESCs and iPSCs, grown on feeder-free media, with Methyl Easy Xceed Rapid DNA Bisulfite Modification Kit (Human Genetic Signatures, Sydney, New South Wales, Australia) per manufacturers directions. The promoter regions of Oct3/4 and Nanog were amplified by PCR, as described by Deb-Rinker et al.^1^. The PCR products were subcloned into pCR2.1 TOPO (Invitrogen), and twelve clones from each sample were analyzed by sequencing with M13 universal primer.

**Spectral Karyotyping**

To prepare metaphase spread, 10μl/mL colcemid was added to the cell culture and incubate for up to 2 hours. The growth medium was removed and collected while the cells were rinsed with HBSS. Cells were treated with 2 mL trypsin and reincubated at 37ºC for 5-7 min. The collected colcemid medium from the earlier step was re-applied to the cells to stop neutralize the trypsin and resuspend the cells. Resulting solution was centrifuged at 1000 RPM for 6 min and the supernatant was partially aspirated and resuspended in the native solution by flicking the tube. 5 drops of pre-warmed hypotonic solution were slowly added against the side, while flicking with a finger, until 1ml had been added. Volume was then brought to 2 mL with hypotonic solution. The sample was incubate at 37ºC for 7min and then centrifuged at 1000 RMP for 6 min. Medium was incompletely to resuspend the cells. To fix the cells, 5 drops of fixative were slowly added against the side of the tube and the volume was brought to 2 mL with fixative. “Reverse bubbled” to fully mix the cells and then the cells were left to fix for 30min at room temperature. After fixing, the sample was centrifuged, aspirated, and resuspended with finger as before. Any clumps were removed by vacuum from the side of tube, and 2ml of fixative were added to the tube. Sample was then “Reverse bubbled”, let stand for 20 min at room temperature, and then centrifuged, aspirated, and resuspended with finger, as before. Sample was resuspended in 2ml of fixative and then transferred onto pre-cleaned slides in ~100ul drops, left to dry overnight, and then analyzed on a SKY microscope. (Spectral-Imaging, Vista, CA)

**SNCA Triplication Confirmation**

SNCA gene copy number analysis: Genomic DNA was isolated from frozen cell pellets using a DNeasy Blood & Tissue Kit (Qiagen 69504). A FAM-MGB Taqman Copy Number Assay against the 3^rd^ intron of SNCA (Hs04791950_cn) and a VIC-TAMRA Taqman Copy Reference Assay against RNase P (#4403326) were used with Taqman Genotyping MasterMix (#4371353, all from Applied Biosystems) to co-amplify 20ng of genomic DNA in quintuplicate from each sample on a Stratagene Mx3000P RT-PCR System. The absolute copy number of SNCA for each sample is reported by calculating the relative copy number (SNCA/RNase P) for each reaction (2^-∆CT^) and then adjusting by a factor of 2 which reflects the normal allelic copy number of RNase P in the genome. 2 alleles of SNCA are normally present, a duplication of SNCA yields 3 alleles, and a triplication of SNCA yields 4 alleles.

**Exogenous and Endogenous Expression Analysis**

Primer sequences used for determining exogenous and endogenous expression of the Oct4, Sox2, Klf4 and c-Myc. Exogenous: pMXs-AS3200: ttatcgtcgaccactgtgctgctg (Used as Reverse primer for all) Oct4- Forward: ccccagggccccattttggtacc. Sox2- Forward: ggcacccctggcatggctcttggctc. Klf4- Forward: acgatcgtggccccggaaaaggacc. cMyc-Forward: caacaaccgaaaatgcaccagccccag. Endogenous: Oct4-Reverse: cctagctcctcccctccccctgtc. (Use the above primers as Forward) Sox2-Reverse: cctcttttgcacccctcccatttccc. Klf4-Reverse: tgattgtagtgctttctggctgggctcc. cMyc-Reverse: ttgaggggcatcgtcgcgggaggctg.

**HPLC Dopamine Release:**

At 60DIV, media was removed and 1ml of N2 supplemented with 56mM KCl was added per well (6 well dish) and incubated at 37oC for 15 minutes. This was collected and immediately frozen in liquid nitrogen and stored at -80oC until assay. Subsequently, protein lysates of each well were made in ice cold 25mM Tris supplemented with a Complete Mini protease inhibitor cocktail tablet (Roche). Lysates sonicated and cleared by maximum speed centrifugation in a tabletop microcentrifuge. Soluble protein concentrations were measured by a standard Bradford assay. KCl samples were thawed, stabilized at a final concentration of 0.4N perchloric acid, and centrifuged at 15,000 rpm at 4oC for 12 minutes to clear debries. The supernatant was then collected and dopamine was assayed by HPLC with electrochemical detection (Coularray detector, ESA, Chelmsford, MA) using a reverse phase C18 column (Perkin Elmer Instruments, Shelton, CT). The mobile phase consisted of a mixture of 90 mM sodium acetate, 35 mM citric acid, 130 uM ethylenediaminetetraaceticacid (EDTA), 230 uM 1-octanesulfonic acid and 10% (v/v) methanol, with a flow rate of 1mL/min. DA concentration was quantified by comparison of AUC to known standard dilutions. Well to well variation was adjusted by protein determinations of cleared protein lysates.

**Probes Used in Microfluidic Fluidigm qPCR Assay:**

| **Category** |  | **Gene** | **Catalog Number** | **>= 30% Valid CT Counts** |
| --- | --- | --- | --- | --- |
| ***Reference Primers*** | |  |  |  |
|  |  | CENTRIN | Hs00189076_m1 | x |
|  |  | CTNNB1 | Hs00170025_m1 | x |
|  |  | EEF1A1 | Hs00742749_s1 | x |
|  |  | GAPDH | [Hs99999905_m1](https://products.appliedbiosystems.com/ab/en/US/direct/ab?cmd=ABAssayDetailDisplay&assayID=Hs99999905_m1&Fs=y&adv_phrase3=EXACT&adv_phrase2=EXACT&adv_phrase1=EXACT&assayType=ge&catID=601267&SearchRequest.Common.SortSpec=&searchValue=null&MFCSpeciesType=null&searchBy=null&adv_kw_filter3=all&srchType=keyword&adv_kw_filter2=all&group1=noValue_limit_sets&SearchRequest.Common.QueryText=gapdh&adv_kw_filter1=all&inventoried=1&adv_query_text3=&species=Homo+sapiens&adv_query_text2=&adv_query_text1=&paraTreeViewNode=null&adv_boolean3=AND&displayAdvSearchResults=&SearchRequest.Common.ResultsPerPage=25&adv_boolean2=AND&adv_boolean1=AND&chkBatchQueryText=false&kwfilter=all&SearchRequest.Common.PageNumber=1&formatType=default&isSL=N&ASSAY_ATTRIBUTE=40_Inventoried&) | x |
|  |  | RPLPO | 4333761F | x |
|  |  |  |  |  |
| ***ES Cell Primers*** |  |  |  |  |
|  |  | C-MYC | Hs00153408_m1 | x |
|  |  | DNMT3B | Hs00171876_m1 | x |
|  |  | FOXD3 | Hs00255287_s1 | x |
|  |  | KLF4 | Hs00358836_m1 | x |
|  |  | LIN28 | Hs00702808_s1 | x |
|  |  | NANOG | Hs02387400_g1 | x |
|  |  | OCT4 | [Hs03005111_g1](https://products.appliedbiosystems.com/ab/en/US/adirect/ab?cmd=ABAssayDetailDisplay&assayID=Hs03005111_g1&Fs=y&adv_phrase3=EXACT&adv_phrase2=EXACT&adv_phrase1=EXACT&assayType=ge&catID=601267&SearchRequest.Common.SortSpec=&searchValue=null&MFCSpeciesType=null&searchBy=null&adv_kw_filter3=all&srchType=keyword&adv_kw_filter2=all&group1=noValue_limit_sets&SearchRequest.Common.QueryText=oct4&adv_kw_filter1=all&inventoried=1&adv_query_text3=&species=Homo+sapiens&adv_query_text2=&adv_query_text1=&paraTreeViewNode=null&adv_boolean3=AND&displayAdvSearchResults=&SearchRequest.Common.ResultsPerPage=25&adv_boolean2=AND&adv_boolean1=AND&chkBatchQueryText=false&kwfilter=all&SearchRequest.Common.PageNumber=1&formatType=default&isSL=N&ASSAY_ATTRIBUTE=40_Inventoried&) | x |
|  |  | TERT | Hs00162669_m1 | x |
|  |  |  |  |  |
| ***Endodermal Markers*** | |  |  |  |
|  |  | GATA6 | [Hs00232018_m1](https://products.appliedbiosystems.com/ab/en/US/direct/ab?cmd=ABAssayDetailDisplay&assayID=Hs00232018_m1&Fs=y&adv_phrase3=EXACT&adv_phrase2=EXACT&adv_phrase1=EXACT&assayType=ge&catID=601267&SearchRequest.Common.SortSpec=&searchValue=null&MFCSpeciesType=null&searchBy=null&adv_kw_filter3=all&srchType=keyword&adv_kw_filter2=all&group1=noValue_limit_sets&SearchRequest.Common.QueryText=gata6&adv_kw_filter1=all&inventoried=1&adv_query_text3=&species=Homo+sapiens&adv_query_text2=&adv_query_text1=&paraTreeViewNode=null&adv_boolean3=AND&displayAdvSearchResults=&SearchRequest.Common.ResultsPerPage=25&adv_boolean2=AND&adv_boolean1=AND&chkBatchQueryText=false&kwfilter=all&SearchRequest.Common.PageNumber=1&formatType=default&isSL=N&ASSAY_ATTRIBUTE=40_Inventoried&) | x |
|  |  | PDX1 | [Hs00426216_m1](https://products.appliedbiosystems.com/ab/en/US/direct/ab?cmd=ABAssayDetailDisplay&assayID=Hs00426216_m1&Fs=y&adv_phrase3=EXACT&adv_phrase2=EXACT&adv_phrase1=EXACT&assayType=ge&catID=601267&SearchRequest.Common.SortSpec=&searchValue=null&MFCSpeciesType=null&searchBy=null&adv_kw_filter3=all&srchType=keyword&adv_kw_filter2=all&group1=noValue_limit_sets&SearchRequest.Common.QueryText=pdx-1&adv_kw_filter1=all&inventoried=1&adv_query_text3=&species=Homo+sapiens&adv_query_text2=&adv_query_text1=&paraTreeViewNode=null&adv_boolean3=AND&displayAdvSearchResults=&SearchRequest.Common.ResultsPerPage=25&adv_boolean2=AND&adv_boolean1=AND&chkBatchQueryText=false&kwfilter=all&SearchRequest.Common.PageNumber=1&formatType=default&isSL=N&ASSAY_ATTRIBUTE=40_Inventoried&) |  |
|  |  | SOX17 | [Hs00751752_s1](https://products.appliedbiosystems.com/ab/en/US/direct/ab?cmd=ABAssayDetailDisplay&assayID=Hs00751752_s1&Fs=y&adv_phrase3=EXACT&adv_phrase2=EXACT&adv_phrase1=EXACT&assayType=ge&catID=601267&SearchRequest.Common.SortSpec=&searchValue=null&MFCSpeciesType=null&searchBy=null&adv_kw_filter3=all&srchType=keyword&adv_kw_filter2=all&group1=noValue_limit_sets&SearchRequest.Common.QueryText=sox17&adv_kw_filter1=all&inventoried=1&adv_query_text3=&species=Homo+sapiens&adv_query_text2=&adv_query_text1=&paraTreeViewNode=null&adv_boolean3=AND&displayAdvSearchResults=&SearchRequest.Common.ResultsPerPage=25&adv_boolean2=AND&adv_boolean1=AND&chkBatchQueryText=false&kwfilter=all&SearchRequest.Common.PageNumber=1&formatType=default&isSL=N&ASSAY_ATTRIBUTE=40_Inventoried&) | x |
|  |  |  |  |  |
| ***Mesodermal Markers*** | |  |  |  |
|  |  | BRACHURY | [Hs00610080_m1](https://products.appliedbiosystems.com/ab/en/US/direct/ab?cmd=ABAssayDetailDisplay&assayID=Hs00610080_m1&Fs=y&adv_phrase3=EXACT&adv_phrase2=EXACT&adv_phrase1=EXACT&assayType=ge&catID=601267&SearchRequest.Common.SortSpec=&searchValue=null&MFCSpeciesType=null&searchBy=null&adv_kw_filter3=all&srchType=keyword&adv_kw_filter2=all&group1=noValue_limit_sets&SearchRequest.Common.QueryText=brachyury&adv_kw_filter1=all&inventoried=1&adv_query_text3=&species=Homo+sapiens&adv_query_text2=&adv_query_text1=&paraTreeViewNode=null&adv_boolean3=AND&displayAdvSearchResults=&SearchRequest.Common.ResultsPerPage=25&adv_boolean2=AND&adv_boolean1=AND&chkBatchQueryText=false&kwfilter=all&SearchRequest.Common.PageNumber=1&formatType=default&isSL=N&ASSAY_ATTRIBUTE=40_Inventoried&) |  |
|  |  | GATA1 | [Hs00231112_m1](https://products.appliedbiosystems.com/ab/en/US/direct/ab?cmd=ABAssayDetailDisplay&assayID=Hs00231112_m1&Fs=y&adv_phrase3=EXACT&adv_phrase2=EXACT&adv_phrase1=EXACT&assayType=ge&catID=601267&SearchRequest.Common.SortSpec=&searchValue=null&MFCSpeciesType=null&searchBy=null&adv_kw_filter3=all&srchType=keyword&adv_kw_filter2=all&group1=noValue_limit_sets&SearchRequest.Common.QueryText=gata1&adv_kw_filter1=all&inventoried=1&adv_query_text3=&species=Homo+sapiens&adv_query_text2=&adv_query_text1=&paraTreeViewNode=null&adv_boolean3=AND&displayAdvSearchResults=&SearchRequest.Common.ResultsPerPage=25&adv_boolean2=AND&adv_boolean1=AND&chkBatchQueryText=false&kwfilter=all&SearchRequest.Common.PageNumber=1&formatType=default&isSL=N&ASSAY_ATTRIBUTE=40_Inventoried&) |  |
|  |  |  |  |  |
| ***Neural Crest Markers*** | |  |  |  |
|  |  | HNK1 | Hs00218629_m1 |  |
|  |  | NCAM | Hs00941821_m1 | x |
|  |  | PRPH | Hs00196608_m1 | x |
|  |  |  |  |  |
| ***Neural Progenitor Primers*** | |  |  |  |
|  |  | NES | [Hs00707120_s1](https://products.appliedbiosystems.com/ab/en/US/adirect/ab?cmd=ABAssayDetailDisplay&assayID=Hs00707120_s1&Fs=y&adv_phrase3=EXACT&adv_phrase2=EXACT&adv_phrase1=EXACT&assayType=ge&catID=601267&SearchRequest.Common.SortSpec=&searchValue=null&MFCSpeciesType=null&searchBy=null&adv_kw_filter3=all&srchType=keyword&adv_kw_filter2=all&group1=noValue&SearchRequest.Common.QueryText=nestin&adv_kw_filter1=all&inventoried=*&adv_query_text3=&species=Homo+sapiens&adv_query_text2=&adv_query_text1=&paraTreeViewNode=null&adv_boolean3=AND&displayAdvSearchResults=&SearchRequest.Common.ResultsPerPage=25&adv_boolean2=AND&adv_boolean1=AND&chkBatchQueryText=false&kwfilter=all&SearchRequest.Common.PageNumber=1&formatType=default&isSL=N&) | x |
|  |  | SOX1 | [Hs01057642_s1](https://products.appliedbiosystems.com/ab/en/US/adirect/ab?cmd=ABAssayDetailDisplay&assayID=Hs01057642_s1&Fs=y&catID=601267&SearchRequest.Common.PageNumber=1&assayType=&chkBatchQueryText=false&srchType=keyword&searchValue=Hs01057642_s1&searchBy=all&) | x |
|  |  | SOX2 | [Hs01053049_s1](https://products.appliedbiosystems.com/ab/en/US/direct/ab?cmd=ABAssayDetailDisplay&assayID=Hs01053049_s1&Fs=y&adv_phrase3=EXACT&adv_phrase2=EXACT&adv_phrase1=EXACT&assayType=ge&catID=601267&SearchRequest.Common.SortSpec=&searchValue=null&MFCSpeciesType=null&searchBy=null&adv_kw_filter3=all&srchType=keyword&adv_kw_filter2=all&group1=noValue_limit_sets&SearchRequest.Common.QueryText=sox2&adv_kw_filter1=all&inventoried=1&adv_query_text3=&species=Homo+sapiens&adv_query_text2=&adv_query_text1=&paraTreeViewNode=null&adv_boolean3=AND&displayAdvSearchResults=&SearchRequest.Common.ResultsPerPage=25&adv_boolean2=AND&adv_boolean1=AND&chkBatchQueryText=false&kwfilter=all&SearchRequest.Common.PageNumber=1&formatType=default&isSL=N&ASSAY_ATTRIBUTE=40_Inventoried&) | x |
|  |  |  |  |  |
| ***Dopamine Neuron Related Primers*** | |  |  |  |
|  |  | ALDH1A1 | [Hs00167445_m1](https://products.appliedbiosystems.com/ab/en/US/adirect/ab?cmd=ABAssayDetailDisplay&assayID=Hs00167445_m1&Fs=y&adv_phrase3=EXACT&adv_phrase2=EXACT&adv_phrase1=EXACT&assayType=ge&catID=601267&SearchRequest.Common.SortSpec=&searchValue=null&MFCSpeciesType=null&searchBy=null&adv_kw_filter3=all&srchType=keyword&adv_kw_filter2=all&group1=noValue&SearchRequest.Common.QueryText=raldh1&adv_kw_filter1=all&inventoried=*&adv_query_text3=&species=Homo+sapiens&adv_query_text2=&adv_query_text1=&paraTreeViewNode=null&adv_boolean3=AND&displayAdvSearchResults=&SearchRequest.Common.ResultsPerPage=25&adv_boolean2=AND&adv_boolean1=AND&chkBatchQueryText=false&kwfilter=all&SearchRequest.Common.PageNumber=1&formatType=default&isSL=N&) | x |
|  |  | BDNF | [Hs00380947_m1](https://products.appliedbiosystems.com/ab/en/US/adirect/ab?cmd=ABAssayDetailDisplay&assayID=Hs00380947_m1&Fs=y&adv_phrase3=EXACT&adv_phrase2=EXACT&adv_phrase1=EXACT&assayType=ge&catID=601267&SearchRequest.Common.SortSpec=&searchValue=null&MFCSpeciesType=null&searchBy=null&adv_kw_filter3=all&srchType=keyword&adv_kw_filter2=all&group1=noValue&SearchRequest.Common.QueryText=bdnf&adv_kw_filter1=all&inventoried=*&adv_query_text3=&species=Homo+sapiens&adv_query_text2=&adv_query_text1=&paraTreeViewNode=null&adv_boolean3=AND&displayAdvSearchResults=&SearchRequest.Common.ResultsPerPage=25&adv_boolean2=AND&adv_boolean1=AND&chkBatchQueryText=false&kwfilter=all&SearchRequest.Common.PageNumber=1&formatType=default&isSL=N&) | x |
|  |  | CALB1 | [Hs00191821_m1](https://products.appliedbiosystems.com/ab/en/US/adirect/ab?cmd=ABAssayDetailDisplay&assayID=Hs00191821_m1&Fs=y&adv_phrase3=EXACT&adv_phrase2=EXACT&adv_phrase1=EXACT&assayType=ge&catID=601267&SearchRequest.Common.SortSpec=&searchValue=null&MFCSpeciesType=null&searchBy=null&adv_kw_filter3=all&srchType=keyword&adv_kw_filter2=all&group1=noValue&SearchRequest.Common.QueryText=calbindin&adv_kw_filter1=all&inventoried=*&adv_query_text3=&species=Homo+sapiens&adv_query_text2=&adv_query_text1=&paraTreeViewNode=null&adv_boolean3=AND&displayAdvSearchResults=&SearchRequest.Common.ResultsPerPage=25&adv_boolean2=AND&adv_boolean1=AND&chkBatchQueryText=false&kwfilter=all&SearchRequest.Common.PageNumber=1&formatType=default&isSL=N&) | x |
|  |  | DAT | [Hs00168988_m1](https://products.appliedbiosystems.com/ab/en/US/adirect/ab?cmd=ABAssayDetailDisplay&assayID=Hs00168988_m1&Fs=y&adv_phrase3=EXACT&adv_phrase2=EXACT&adv_phrase1=EXACT&assayType=ge&catID=601267&MFCSpeciesType=null&adv_kw_filter3=all&srchType=keyword&adv_kw_filter2=all&group1=noValue&SearchRequest.Common.QueryText=dopamine&adv_kw_filter1=all&species=Homo+sapiens&adv_query_text3=&adv_query_text2=&adv_query_text1=&adv_boolean3=AND&adv_boolean2=AND&batchSpecies=&adv_boolean1=AND&chkBatchQueryText=false&kwfilter=all&SearchRequest.Common.PageNumber=1&formatType=default&) | x |
|  |  | DDC | [Hs00168031_m1](https://products.appliedbiosystems.com/ab/en/US/adirect/ab?cmd=ABAssayDetailDisplay&assayID=Hs00168031_m1&Fs=y&adv_phrase3=EXACT&adv_phrase2=EXACT&adv_phrase1=EXACT&assayType=ge&catID=601267&SearchRequest.Common.SortSpec=&searchValue=null&MFCSpeciesType=null&searchBy=null&adv_kw_filter3=all&srchType=keyword&adv_kw_filter2=all&group1=noValue&SearchRequest.Common.QueryText=aadc&adv_kw_filter1=all&inventoried=*&adv_query_text3=&species=Homo+sapiens&adv_query_text2=&adv_query_text1=&paraTreeViewNode=null&adv_boolean3=AND&displayAdvSearchResults=&SearchRequest.Common.ResultsPerPage=25&adv_boolean2=AND&adv_boolean1=AND&chkBatchQueryText=false&kwfilter=all&SearchRequest.Common.PageNumber=1&formatType=default&isSL=N&) | x |
|  |  | DRD2 | [Hs00241436_m1](https://products.appliedbiosystems.com/ab/en/US/direct/ab?cmd=ABAssayDetailDisplay&assayID=Hs00241436_m1&Fs=y&adv_phrase3=EXACT&adv_phrase2=EXACT&adv_phrase1=EXACT&assayType=ge&catID=601267&SearchRequest.Common.SortSpec=&searchValue=null&MFCSpeciesType=null&searchBy=null&adv_kw_filter3=all&srchType=keyword&adv_kw_filter2=all&group1=noValue_limit_sets&SearchRequest.Common.QueryText=dopamine+receptor&adv_kw_filter1=all&inventoried=1&adv_query_text3=&species=Homo+sapiens&adv_query_text2=&adv_query_text1=&paraTreeViewNode=null&adv_boolean3=AND&displayAdvSearchResults=&SearchRequest.Common.ResultsPerPage=25&adv_boolean2=AND&adv_boolean1=AND&chkBatchQueryText=false&kwfilter=all&SearchRequest.Common.PageNumber=1&formatType=default&isSL=N&ASSAY_ATTRIBUTE=40_Inventoried&) | x |
|  |  | EN1 | [Hs00154977_m1](https://products.appliedbiosystems.com/ab/en/US/adirect/ab?cmd=ABAssayDetailDisplay&assayID=Hs00154977_m1&Fs=y&SearchRequest.Common.PageNumber=1&assayType=ge&chkBatchQueryText=false&SearchRequest.Common.QueryText=engrailed&srchType=keyword&catID=601267&kwfilter=GENE_NAME&) | x |
|  |  | EN2 | [Hs00171321_m1](https://products.appliedbiosystems.com/ab/en/US/adirect/ab?cmd=ABAssayDetailDisplay&assayID=Hs00171321_m1&Fs=y&SearchRequest.Common.PageNumber=1&assayType=ge&chkBatchQueryText=false&SearchRequest.Common.QueryText=engrailed&srchType=keyword&catID=601267&kwfilter=GENE_NAME&) | x |
|  |  | FOXA1 | [Hs00270129_m1](https://products.appliedbiosystems.com/ab/en/US/adirect/ab?cmd=ABAssayDetailDisplay&assayID=Hs00270129_m1&Fs=y&adv_phrase3=EXACT&adv_phrase2=EXACT&adv_phrase1=EXACT&assayType=ge&catID=601267&SearchRequest.Common.SortSpec=&searchValue=null&MFCSpeciesType=null&searchBy=null&adv_kw_filter3=all&srchType=keyword&adv_kw_filter2=all&group1=noValue&SearchRequest.Common.QueryText=foxa1&adv_kw_filter1=all&adv_query_text3=&species=Homo+sapiens&adv_query_text2=&adv_query_text1=&paraTreeViewNode=null&adv_boolean3=AND&displayAdvSearchResults=&adv_boolean2=AND&adv_boolean1=AND&chkBatchQueryText=false&kwfilter=all&SearchRequest.Common.PageNumber=1&formatType=default&isSL=N&) | x |
|  |  | FOXA2 | [Hs00232764_m1](https://products.appliedbiosystems.com/ab/en/US/adirect/ab?cmd=ABAssayDetailDisplay&assayID=Hs00232764_m1&Fs=y&adv_phrase3=EXACT&adv_phrase2=EXACT&adv_phrase1=EXACT&assayType=ge&catID=601267&SearchRequest.Common.SortSpec=&searchValue=null&MFCSpeciesType=null&searchBy=null&adv_kw_filter3=all&srchType=keyword&adv_kw_filter2=all&group1=noValue&SearchRequest.Common.QueryText=foxa2&adv_kw_filter1=all&adv_query_text3=&species=Homo+sapiens&adv_query_text2=&adv_query_text1=&paraTreeViewNode=null&adv_boolean3=AND&displayAdvSearchResults=&adv_boolean2=AND&adv_boolean1=AND&chkBatchQueryText=false&kwfilter=all&SearchRequest.Common.PageNumber=1&formatType=default&isSL=N&) | x |
|  |  | GDNF | [Hs00181185_m1](https://products.appliedbiosystems.com/ab/en/US/adirect/ab?cmd=ABAssayDetailDisplay&assayID=Hs00181185_m1&Fs=y&adv_phrase3=EXACT&adv_phrase2=EXACT&adv_phrase1=EXACT&assayType=ge&catID=601267&SearchRequest.Common.SortSpec=&searchValue=null&MFCSpeciesType=null&searchBy=null&adv_kw_filter3=all&srchType=keyword&adv_kw_filter2=all&group1=noValue&SearchRequest.Common.QueryText=gdnf&adv_kw_filter1=all&inventoried=*&adv_query_text3=&species=Homo+sapiens&adv_query_text2=&adv_query_text1=&paraTreeViewNode=null&adv_boolean3=AND&displayAdvSearchResults=&SearchRequest.Common.ResultsPerPage=25&adv_boolean2=AND&adv_boolean1=AND&chkBatchQueryText=false&kwfilter=all&SearchRequest.Common.PageNumber=1&formatType=default&isSL=N&) | x |
|  |  | GIRK2 | [Hs00158423_m1](https://products.appliedbiosystems.com/ab/en/US/adirect/ab?cmd=ABAssayDetailDisplay&assayID=Hs00158423_m1&Fs=y&adv_phrase3=EXACT&adv_phrase2=EXACT&adv_phrase1=EXACT&assayType=ge&catID=601267&SearchRequest.Common.SortSpec=&searchValue=null&MFCSpeciesType=null&searchBy=null&adv_kw_filter3=all&srchType=keyword&adv_kw_filter2=all&group1=noValue&SearchRequest.Common.QueryText=girk2&adv_kw_filter1=all&inventoried=*&adv_query_text3=&species=Homo+sapiens&adv_query_text2=&adv_query_text1=&paraTreeViewNode=null&adv_boolean3=AND&displayAdvSearchResults=&SearchRequest.Common.ResultsPerPage=25&adv_boolean2=AND&adv_boolean1=AND&chkBatchQueryText=false&kwfilter=all&SearchRequest.Common.PageNumber=1&formatType=default&isSL=N&) | x |
|  |  | LMX1A | [Hs00602600_m1](https://products.appliedbiosystems.com/ab/en/US/adirect/ab?cmd=ABAssayDetailDisplay&assayID=Hs00602600_m1&Fs=y&adv_phrase3=EXACT&adv_phrase2=EXACT&adv_phrase1=EXACT&assayType=ge&catID=601267&SearchRequest.Common.SortSpec=&searchValue=null&MFCSpeciesType=null&searchBy=null&adv_kw_filter3=all&srchType=keyword&adv_kw_filter2=all&group1=noValue&SearchRequest.Common.QueryText=lmx1a&adv_kw_filter1=all&adv_query_text3=&species=Homo+sapiens&adv_query_text2=&adv_query_text1=&paraTreeViewNode=null&adv_boolean3=AND&displayAdvSearchResults=&adv_boolean2=AND&adv_boolean1=AND&chkBatchQueryText=false&kwfilter=all&SearchRequest.Common.PageNumber=1&formatType=default&isSL=N&) | x |
|  |  | LMX1B | [Hs00158750_m1](https://products.appliedbiosystems.com/ab/en/US/adirect/ab?cmd=ABAssayDetailDisplay&assayID=Hs00158750_m1&Fs=y&adv_phrase3=EXACT&adv_phrase2=EXACT&adv_phrase1=EXACT&assayType=ge&catID=601267&SearchRequest.Common.SortSpec=&searchValue=null&MFCSpeciesType=null&searchBy=null&adv_kw_filter3=all&srchType=keyword&adv_kw_filter2=all&group1=noValue&SearchRequest.Common.QueryText=lmx1b&adv_kw_filter1=all&inventoried=*&adv_query_text3=&species=Homo+sapiens&adv_query_text2=&adv_query_text1=&paraTreeViewNode=null&adv_boolean3=AND&displayAdvSearchResults=&SearchRequest.Common.ResultsPerPage=25&adv_boolean2=AND&adv_boolean1=AND&chkBatchQueryText=false&kwfilter=all&SearchRequest.Common.PageNumber=1&formatType=default&isSL=N&) |  |
|  |  | MASH1 | [Hs00269932_m1](https://products.appliedbiosystems.com/ab/en/US/direct/ab?cmd=ABAssayDetailDisplay&assayID=Hs00269932_m1&Fs=y&adv_phrase3=EXACT&adv_phrase2=EXACT&adv_phrase1=EXACT&assayType=ge&catID=601267&SearchRequest.Common.SortSpec=&searchValue=null&MFCSpeciesType=null&searchBy=null&adv_kw_filter3=all&srchType=keyword&adv_kw_filter2=all&group1=noValue_limit_sets&SearchRequest.Common.QueryText=mash1&adv_kw_filter1=all&inventoried=1&adv_query_text3=&species=Homo+sapiens&adv_query_text2=&adv_query_text1=&paraTreeViewNode=null&adv_boolean3=AND&displayAdvSearchResults=&SearchRequest.Common.ResultsPerPage=25&adv_boolean2=AND&adv_boolean1=AND&chkBatchQueryText=false&kwfilter=all&SearchRequest.Common.PageNumber=1&formatType=default&isSL=N&ASSAY_ATTRIBUTE=40_Inventoried&) |  |
|  |  | MSX1 | [Hs00427183_m1](https://products.appliedbiosystems.com/ab/en/US/direct/ab?cmd=ABAssayDetailDisplay&assayID=Hs00427183_m1&Fs=y&adv_phrase3=EXACT&adv_phrase2=EXACT&adv_phrase1=EXACT&assayType=ge&catID=601267&MFCSpeciesType=null&adv_kw_filter3=all&srchType=keyword&adv_kw_filter2=all&group1=noValue_limit_sets&SearchRequest.Common.QueryText=msx1&adv_kw_filter1=all&species=Homo+sapiens&adv_query_text3=&adv_query_text2=&adv_query_text1=&adv_boolean3=AND&adv_boolean2=AND&batchSpecies=Homo+sapiens&adv_boolean1=AND&chkBatchQueryText=false&kwfilter=all&SearchRequest.Common.PageNumber=1&formatType=default&ASSAY_ATTRIBUTE=40_Inventoried&) | x |
|  |  | MSX2 | [Hs00741177_m1](https://products.appliedbiosystems.com/ab/en/US/direct/ab?cmd=ABAssayDetailDisplay&assayID=Hs00741177_m1&Fs=y&adv_phrase3=EXACT&adv_phrase2=EXACT&adv_phrase1=EXACT&assayType=ge&catID=601267&SearchRequest.Common.SortSpec=&searchValue=null&MFCSpeciesType=null&searchBy=null&adv_kw_filter3=all&srchType=keyword&adv_kw_filter2=all&group1=noValue_limit_sets&SearchRequest.Common.QueryText=msx2&adv_kw_filter1=all&inventoried=1&adv_query_text3=&species=Homo+sapiens&adv_query_text2=&adv_query_text1=&paraTreeViewNode=null&adv_boolean3=AND&displayAdvSearchResults=&SearchRequest.Common.ResultsPerPage=25&adv_boolean2=AND&adv_boolean1=AND&chkBatchQueryText=false&kwfilter=all&SearchRequest.Common.PageNumber=1&formatType=default&isSL=N&ASSAY_ATTRIBUTE=40_Inventoried&) | x |
|  |  | NGN2 | [Hs00702774_s1](https://products.appliedbiosystems.com/ab/en/US/direct/ab?cmd=ABAssayDetailDisplay&assayID=Hs00702774_s1&Fs=y&adv_phrase3=EXACT&adv_phrase2=EXACT&adv_phrase1=EXACT&assayType=ge&catID=601267&SearchRequest.Common.SortSpec=&searchValue=null&MFCSpeciesType=null&searchBy=null&adv_kw_filter3=all&srchType=keyword&adv_kw_filter2=all&group1=noValue_limit_sets&SearchRequest.Common.QueryText=ngn2&adv_kw_filter1=all&inventoried=1&adv_query_text3=&species=Homo+sapiens&adv_query_text2=&adv_query_text1=&paraTreeViewNode=null&adv_boolean3=AND&displayAdvSearchResults=&SearchRequest.Common.ResultsPerPage=25&adv_boolean2=AND&adv_boolean1=AND&chkBatchQueryText=false&kwfilter=all&SearchRequest.Common.PageNumber=1&formatType=default&isSL=N&ASSAY_ATTRIBUTE=40_Inventoried&) | x |
|  |  | NTN1 | [Hs00180355_m1](https://products.appliedbiosystems.com/ab/en/US/adirect/ab?cmd=ABAssayDetailDisplay&assayID=Hs00180355_m1&Fs=y&adv_phrase3=EXACT&adv_phrase2=EXACT&adv_phrase1=EXACT&assayType=ge&catID=601267&SearchRequest.Common.SortSpec=&searchValue=null&MFCSpeciesType=null&searchBy=null&adv_kw_filter3=all&srchType=keyword&adv_kw_filter2=all&group1=noValue&SearchRequest.Common.QueryText=ntn1&adv_kw_filter1=all&adv_query_text3=&species=Homo+sapiens&adv_query_text2=&adv_query_text1=&paraTreeViewNode=null&adv_boolean3=AND&displayAdvSearchResults=&adv_boolean2=AND&adv_boolean1=AND&chkBatchQueryText=false&kwfilter=all&SearchRequest.Common.PageNumber=1&formatType=default&isSL=N&) | x |
|  |  | NTSR1 | [Hs00901551_m1](https://products.appliedbiosystems.com/ab/en/US/adirect/ab?cmd=ABAssayDetailDisplay&assayID=Hs00901551_m1&Fs=y&adv_phrase3=EXACT&adv_phrase2=EXACT&adv_phrase1=EXACT&assayType=ge&catID=601267&SearchRequest.Common.SortSpec=&searchValue=null&MFCSpeciesType=null&searchBy=null&adv_kw_filter3=all&srchType=keyword&adv_kw_filter2=all&group1=noValue_limit_sets&SearchRequest.Common.QueryText=neurotensin&adv_kw_filter1=all&inventoried=1&adv_query_text3=&species=Homo+sapiens&adv_query_text2=&adv_query_text1=&paraTreeViewNode=null&adv_boolean3=AND&displayAdvSearchResults=&SearchRequest.Common.ResultsPerPage=25&adv_boolean2=AND&adv_boolean1=AND&chkBatchQueryText=false&kwfilter=all&SearchRequest.Common.PageNumber=1&formatType=default&isSL=N&ASSAY_ATTRIBUTE=40_Inventoried&) |  |
|  |  | NURR1 | [Hs01118813_m1](https://products.appliedbiosystems.com/ab/en/US/adirect/ab?cmd=ABAssayDetailDisplay&assayID=Hs01118813_m1&Fs=y&SearchRequest.Common.PageNumber=1&assayType=ge&chkBatchQueryText=false&SearchRequest.Common.QueryText=nurr1&srchType=keyword&catID=601267&kwfilter=all&) | x |
|  |  | OTX2 | [Hs00222238_m1](https://products.appliedbiosystems.com/ab/en/US/adirect/ab?cmd=ABAssayDetailDisplay&assayID=Hs00222238_m1&Fs=y&adv_phrase3=EXACT&adv_phrase2=EXACT&adv_phrase1=EXACT&assayType=ge&catID=601267&SearchRequest.Common.SortSpec=&searchValue=null&MFCSpeciesType=null&searchBy=null&adv_kw_filter3=all&srchType=keyword&adv_kw_filter2=all&group1=noValue&SearchRequest.Common.QueryText=otx2&adv_kw_filter1=all&adv_query_text3=&species=Homo+sapiens&adv_query_text2=&adv_query_text1=&paraTreeViewNode=null&adv_boolean3=AND&displayAdvSearchResults=&adv_boolean2=AND&adv_boolean1=AND&chkBatchQueryText=false&kwfilter=all&SearchRequest.Common.PageNumber=1&formatType=default&isSL=N&) | x |
|  |  | PAX2 | [Hs01057416_m1](https://products.appliedbiosystems.com/ab/en/US/direct/ab?cmd=ABAssayDetailDisplay&assayID=Hs01057416_m1&Fs=y&adv_phrase3=EXACT&adv_phrase2=EXACT&adv_phrase1=EXACT&assayType=ge&catID=601267&SearchRequest.Common.SortSpec=&searchValue=null&MFCSpeciesType=null&searchBy=null&adv_kw_filter3=all&srchType=keyword&adv_kw_filter2=all&group1=noValue_limit_sets&SearchRequest.Common.QueryText=pax2&adv_kw_filter1=all&inventoried=1&adv_query_text3=&species=Homo+sapiens&adv_query_text2=&adv_query_text1=&paraTreeViewNode=null&adv_boolean3=AND&displayAdvSearchResults=&SearchRequest.Common.ResultsPerPage=25&adv_boolean2=AND&adv_boolean1=AND&chkBatchQueryText=false&kwfilter=all&SearchRequest.Common.PageNumber=1&formatType=default&isSL=N&ASSAY_ATTRIBUTE=40_Inventoried&) | x |
|  |  | PAX6 | [Hs00240871_m1](https://products.appliedbiosystems.com/ab/en/US/direct/ab?cmd=ABAssayDetailDisplay&assayID=Hs00240871_m1&Fs=y&adv_phrase3=EXACT&adv_phrase2=EXACT&adv_phrase1=EXACT&assayType=ge&catID=601267&SearchRequest.Common.SortSpec=&searchValue=null&MFCSpeciesType=null&searchBy=null&adv_kw_filter3=all&srchType=keyword&adv_kw_filter2=all&group1=noValue_limit_sets&SearchRequest.Common.QueryText=pax6&adv_kw_filter1=all&inventoried=1&adv_query_text3=&species=Homo+sapiens&adv_query_text2=&adv_query_text1=&paraTreeViewNode=null&adv_boolean3=AND&displayAdvSearchResults=&SearchRequest.Common.ResultsPerPage=25&adv_boolean2=AND&adv_boolean1=AND&chkBatchQueryText=false&kwfilter=all&SearchRequest.Common.PageNumber=1&formatType=default&isSL=N&ASSAY_ATTRIBUTE=40_Inventoried&) | x |
|  |  | PITX3 | [Hs00374504_m1](https://products.appliedbiosystems.com/ab/en/US/adirect/ab?cmd=ABAssayDetailDisplay&assayID=Hs00374504_m1&Fs=y&adv_phrase3=EXACT&adv_phrase2=EXACT&adv_phrase1=EXACT&assayType=ge&catID=601267&SearchRequest.Common.SortSpec=&searchValue=null&MFCSpeciesType=null&searchBy=null&adv_kw_filter3=all&srchType=keyword&adv_kw_filter2=all&group1=noValue&SearchRequest.Common.QueryText=pitx3&adv_kw_filter1=all&inventoried=*&adv_query_text3=&species=Homo+sapiens&adv_query_text2=&adv_query_text1=&paraTreeViewNode=null&adv_boolean3=AND&displayAdvSearchResults=&SearchRequest.Common.ResultsPerPage=25&adv_boolean2=AND&adv_boolean1=AND&chkBatchQueryText=false&kwfilter=all&SearchRequest.Common.PageNumber=1&formatType=default&isSL=N&) | x |
|  |  | RET | [Hs00240887_m1](https://products.appliedbiosystems.com/ab/en/US/adirect/ab?cmd=ABAssayDetailDisplay&assayID=Hs00240887_m1&Fs=y&adv_phrase3=EXACT&adv_phrase2=EXACT&adv_phrase1=EXACT&assayType=ge&catID=601267&SearchRequest.Common.SortSpec=&searchValue=null&MFCSpeciesType=null&searchBy=null&adv_kw_filter3=all&srchType=keyword&adv_kw_filter2=all&group1=noValue_limit_sets&SearchRequest.Common.QueryText=ret&adv_kw_filter1=all&adv_query_text3=&species=Homo+sapiens&adv_query_text2=&adv_query_text1=&paraTreeViewNode=null&adv_boolean3=AND&displayAdvSearchResults=&adv_boolean2=AND&adv_boolean1=AND&chkBatchQueryText=false&kwfilter=all&SearchRequest.Common.PageNumber=1&formatType=default&isSL=N&ASSAY_ATTRIBUTE=40_Inventoried&) | x |
|  |  | SHH | [Hs00179843_m1](https://products.appliedbiosystems.com/ab/en/US/adirect/ab?cmd=ABAssayDetailDisplay&assayID=Hs00179843_m1&Fs=y&adv_phrase3=EXACT&adv_phrase2=EXACT&adv_phrase1=EXACT&assayType=ge&catID=601267&SearchRequest.Common.SortSpec=&searchValue=null&MFCSpeciesType=null&searchBy=null&adv_kw_filter3=all&srchType=keyword&adv_kw_filter2=all&group1=noValue&SearchRequest.Common.QueryText=shh&adv_kw_filter1=all&adv_query_text3=&species=Homo+sapiens&adv_query_text2=&adv_query_text1=&paraTreeViewNode=null&adv_boolean3=AND&displayAdvSearchResults=&adv_boolean2=AND&adv_boolean1=AND&chkBatchQueryText=false&kwfilter=all&SearchRequest.Common.PageNumber=1&formatType=default&isSL=N&) | x |
|  |  | SNCG | [Hs00268306_m1](https://products.appliedbiosystems.com/ab/en/US/adirect/ab?cmd=ABAssayDetailDisplay&assayID=Hs00268306_m1&Fs=y&adv_phrase3=EXACT&adv_phrase2=EXACT&adv_phrase1=EXACT&assayType=ge&catID=601267&SearchRequest.Common.SortSpec=&searchValue=null&MFCSpeciesType=null&searchBy=null&adv_kw_filter3=all&srchType=keyword&adv_kw_filter2=all&group1=noValue_limit_sets&SearchRequest.Common.QueryText=sncg&adv_kw_filter1=all&inventoried=1&adv_query_text3=&species=Homo+sapiens&adv_query_text2=&adv_query_text1=&paraTreeViewNode=null&adv_boolean3=AND&displayAdvSearchResults=&SearchRequest.Common.ResultsPerPage=25&adv_boolean2=AND&adv_boolean1=AND&chkBatchQueryText=false&kwfilter=all&SearchRequest.Common.PageNumber=1&formatType=default&isSL=N&ASSAY_ATTRIBUTE=40_Inventoried&) | x |
|  |  | TH | [Hs00165941_m1](https://products.appliedbiosystems.com/ab/en/US/adirect/ab?cmd=ABAssayDetailDisplay&assayID=Hs00165941_m1&Fs=y&catID=601267&SearchRequest.Common.PageNumber=1&assayType=&chkBatchQueryText=false&srchType=keyword&searchValue=tyrosine+hydroxylase&searchBy=all) | x |
|  |  | VMAT2 | [Hs00996835_m1](https://products.appliedbiosystems.com/ab/en/US/adirect/ab?cmd=ABAssayDetailDisplay&assayID=Hs00996835_m1&Fs=y&adv_phrase3=EXACT&adv_phrase2=EXACT&adv_phrase1=EXACT&assayType=ge&catID=601267&SearchRequest.Common.SortSpec=&searchValue=null&MFCSpeciesType=null&searchBy=null&adv_kw_filter3=all&srchType=keyword&adv_kw_filter2=all&group1=noValue_limit_sets&SearchRequest.Common.QueryText=vmat2&adv_kw_filter1=all&inventoried=1&adv_query_text3=&species=Homo+sapiens&adv_query_text2=&adv_query_text1=&paraTreeViewNode=null&adv_boolean3=AND&displayAdvSearchResults=&SearchRequest.Common.ResultsPerPage=25&adv_boolean2=AND&adv_boolean1=AND&chkBatchQueryText=false&kwfilter=all&SearchRequest.Common.PageNumber=1&formatType=default&isSL=N&ASSAY_ATTRIBUTE=40_Inventoried&) | x |
|  |  |  |  |  |
| ***Floorplate Primers*** | |  |  |  |
|  |  | FOXA2 | already listed | x |
|  |  | SPON1 | [Hs00391824_m1](https://products.appliedbiosystems.com/ab/en/US/adirect/ab?cmd=ABAssayDetailDisplay&assayID=Hs00391824_m1&Fs=y&adv_phrase3=EXACT&adv_phrase2=EXACT&adv_phrase1=EXACT&assayType=ge&catID=601267&SearchRequest.Common.SortSpec=&searchValue=null&MFCSpeciesType=null&searchBy=null&adv_kw_filter3=all&srchType=keyword&adv_kw_filter2=all&group1=noValue_limit_sets&SearchRequest.Common.QueryText=f-spondin&adv_kw_filter1=all&adv_query_text3=&species=Homo+sapiens&adv_query_text2=&adv_query_text1=&paraTreeViewNode=null&adv_boolean3=AND&displayAdvSearchResults=&adv_boolean2=AND&adv_boolean1=AND&chkBatchQueryText=false&kwfilter=all&SearchRequest.Common.PageNumber=1&formatType=default&isSL=N&ASSAY_ATTRIBUTE=40_Inventoried&) | x |
|  |  |  |  |  |
| ***Other Neuronal Markers*** | |  |  |  |
|  | *Generic Neuron Primers* | |  |  |
|  |  | B3GAT1 | Hs00218629_m1 | x |
|  |  | MAP2 | [Hs00258900_m1](https://products.appliedbiosystems.com/ab/en/US/adirect/ab?cmd=ABAssayDetailDisplay&assayID=Hs00258900_m1&Fs=y&adv_phrase3=EXACT&adv_phrase2=EXACT&adv_phrase1=EXACT&assayType=ge&catID=601267&SearchRequest.Common.SortSpec=&searchValue=null&MFCSpeciesType=null&searchBy=null&adv_kw_filter3=all&srchType=keyword&adv_kw_filter2=all&group1=noValue_limit_sets&SearchRequest.Common.QueryText=map2&adv_kw_filter1=all&inventoried=1&adv_query_text3=&species=Homo+sapiens&searchType=keyword&adv_query_text2=&adv_query_text1=&paraTreeViewNode=null&adv_boolean3=AND&displayAdvSearchResults=&SearchRequest.Common.ResultsPerPage=25&adv_boolean2=AND&adv_boolean1=AND&chkBatchQueryText=false&kwfilter=all&SearchRequest.Common.PageNumber=1&formatType=default&isSL=N&ASSAY_ATTRIBUTE=40_Inventoried&) | x |
|  |  | NEFL | [Hs00196245_m1](https://products.appliedbiosystems.com/ab/en/US/adirect/ab?cmd=ABAssayDetailDisplay&assayID=Hs00196245_m1&Fs=y&adv_phrase3=EXACT&adv_phrase2=EXACT&adv_phrase1=EXACT&assayType=ge&catID=601267&SearchRequest.Common.SortSpec=&searchValue=null&MFCSpeciesType=null&searchBy=null&adv_kw_filter3=all&srchType=keyword&adv_kw_filter2=all&group1=noValue_limit_sets&SearchRequest.Common.QueryText=neurofilament&adv_kw_filter1=all&adv_query_text3=&species=Homo+sapiens&searchType=keyword&adv_query_text2=&adv_query_text1=&paraTreeViewNode=null&adv_boolean3=AND&displayAdvSearchResults=&adv_boolean2=AND&adv_boolean1=AND&chkBatchQueryText=false&kwfilter=all&SearchRequest.Common.PageNumber=1&formatType=default&isSL=N&ASSAY_ATTRIBUTE=40_Inventoried&) | x |
|  |  |  |  |  |
|  | Hypothalamic DA neurons | |  |  |
|  |  | ISL1 | [Hs00158126_m1](https://products.appliedbiosystems.com/ab/en/US/adirect/ab?cmd=ABAssayDetailDisplay&assayID=Hs00158126_m1&Fs=y&adv_phrase3=EXACT&adv_phrase2=EXACT&adv_phrase1=EXACT&assayType=ge&catID=601267&SearchRequest.Common.SortSpec=&searchValue=null&MFCSpeciesType=null&searchBy=null&adv_kw_filter3=all&srchType=keyword&adv_kw_filter2=all&group1=noValue_limit_sets&SearchRequest.Common.QueryText=isl1&adv_kw_filter1=all&inventoried=1&adv_query_text3=&species=Homo+sapiens&searchType=keyword&adv_query_text2=&adv_query_text1=&paraTreeViewNode=null&adv_boolean3=AND&displayAdvSearchResults=&SearchRequest.Common.ResultsPerPage=25&adv_boolean2=AND&adv_boolean1=AND&chkBatchQueryText=false&kwfilter=all&SearchRequest.Common.PageNumber=1&formatType=default&isSL=N&ASSAY_ATTRIBUTE=40_Inventoried&) | x |
|  |  | LIM1 | [Hs00232144_m1](https://products.appliedbiosystems.com/ab/en/US/adirect/ab?cmd=ABAssayDetailDisplay&assayID=Hs00232144_m1&Fs=y&adv_phrase3=EXACT&adv_phrase2=EXACT&adv_phrase1=EXACT&assayType=ge&catID=601267&SearchRequest.Common.SortSpec=&searchValue=null&MFCSpeciesType=null&searchBy=null&adv_kw_filter3=all&srchType=keyword&adv_kw_filter2=all&group1=noValue_limit_sets&SearchRequest.Common.QueryText=lim1&adv_kw_filter1=all&inventoried=1&adv_query_text3=&species=Homo+sapiens&searchType=keyword&adv_query_text2=&adv_query_text1=&paraTreeViewNode=null&adv_boolean3=AND&displayAdvSearchResults=&SearchRequest.Common.ResultsPerPage=25&adv_boolean2=AND&adv_boolean1=AND&chkBatchQueryText=false&kwfilter=all&SearchRequest.Common.PageNumber=1&formatType=default&isSL=N&ASSAY_ATTRIBUTE=40_Inventoried&) |  |
|  |  | NKX2.1 | [Hs00163037_m1](https://products.appliedbiosystems.com/ab/en/US/adirect/ab?cmd=ABAssayDetailDisplay&assayID=Hs00163037_m1&Fs=y&adv_phrase3=EXACT&adv_phrase2=EXACT&adv_phrase1=EXACT&assayType=ge&catID=601267&SearchRequest.Common.SortSpec=&searchValue=null&MFCSpeciesType=null&searchBy=null&adv_kw_filter3=all&srchType=keyword&adv_kw_filter2=all&group1=noValue_limit_sets&SearchRequest.Common.QueryText=%27nkx2.1%27&adv_kw_filter1=all&inventoried=1&adv_query_text3=&species=Homo+sapiens&searchType=keyword&adv_query_text2=&adv_query_text1=&paraTreeViewNode=null&adv_boolean3=AND&displayAdvSearchResults=&SearchRequest.Common.ResultsPerPage=25&adv_boolean2=AND&adv_boolean1=AND&chkBatchQueryText=false&kwfilter=all&SearchRequest.Common.PageNumber=1&formatType=default&isSL=N&ASSAY_ATTRIBUTE=40_Inventoried&) |  |
|  |  | PROX1 | [Hs00160463_m1](https://products.appliedbiosystems.com/ab/en/US/adirect/ab?cmd=ABAssayDetailDisplay&assayID=Hs00160463_m1&Fs=y&adv_phrase3=EXACT&adv_phrase2=EXACT&adv_phrase1=EXACT&assayType=ge&catID=601267&SearchRequest.Common.SortSpec=&searchValue=null&MFCSpeciesType=null&searchBy=null&adv_kw_filter3=all&srchType=keyword&adv_kw_filter2=all&group1=noValue_limit_sets&SearchRequest.Common.QueryText=prox1&adv_kw_filter1=all&adv_query_text3=&species=Homo+sapiens&searchType=keyword&adv_query_text2=&adv_query_text1=&paraTreeViewNode=null&adv_boolean3=AND&displayAdvSearchResults=&adv_boolean2=AND&adv_boolean1=AND&chkBatchQueryText=false&kwfilter=all&SearchRequest.Common.PageNumber=1&formatType=default&isSL=N&ASSAY_ATTRIBUTE=40_Inventoried&) | x |
|  |  |  |  |  |
|  | GABAergic Neurons | |  |  |
|  |  | GAD1 | [Hs01065893_m1](https://products.appliedbiosystems.com/ab/en/US/direct/ab?cmd=ABAssayDetailDisplay&assayID=Hs01065893_m1&Fs=y&adv_phrase3=EXACT&adv_phrase2=EXACT&adv_phrase1=EXACT&assayType=ge&catID=601267&SearchRequest.Common.SortSpec=&searchValue=null&MFCSpeciesType=null&searchBy=null&adv_kw_filter3=all&srchType=keyword&adv_kw_filter2=all&group1=noValue_limit_sets&SearchRequest.Common.QueryText=gad&adv_kw_filter1=all&adv_query_text3=&species=Homo+sapiens&adv_query_text2=&adv_query_text1=&paraTreeViewNode=null&adv_boolean3=AND&displayAdvSearchResults=&adv_boolean2=AND&adv_boolean1=AND&chkBatchQueryText=false&kwfilter=all&SearchRequest.Common.PageNumber=1&formatType=default&isSL=N&ASSAY_ATTRIBUTE=40_Inventoried&) | x |
|  |  | GAD2 | [Hs00609534_m1](https://products.appliedbiosystems.com/ab/en/US/direct/ab?cmd=ABAssayDetailDisplay&assayID=Hs00609534_m1&Fs=y&adv_phrase3=EXACT&adv_phrase2=EXACT&adv_phrase1=EXACT&assayType=ge&catID=601267&SearchRequest.Common.SortSpec=&searchValue=null&MFCSpeciesType=null&searchBy=null&adv_kw_filter3=all&srchType=keyword&adv_kw_filter2=all&group1=noValue_limit_sets&SearchRequest.Common.QueryText=gad65&adv_kw_filter1=all&adv_query_text3=&species=Homo+sapiens&adv_query_text2=&adv_query_text1=&paraTreeViewNode=null&adv_boolean3=AND&displayAdvSearchResults=&adv_boolean2=AND&adv_boolean1=AND&chkBatchQueryText=false&kwfilter=all&SearchRequest.Common.PageNumber=1&formatType=default&isSL=N&ASSAY_ATTRIBUTE=40_Inventoried&) | x |
|  |  |  |  |  |
|  | Epi/NE Neurons |  |  |  |
|  |  | DBH | [Hs01089840_m1](https://products.appliedbiosystems.com/ab/en/US/adirect/ab?cmd=ABAssayDetailDisplay&assayID=Hs01089840_m1&Fs=y&adv_phrase3=EXACT&adv_phrase2=EXACT&adv_phrase1=EXACT&assayType=ge&catID=601267&MFCSpeciesType=null&adv_kw_filter3=all&srchType=keyword&adv_kw_filter2=all&group1=noValue&SearchRequest.Common.QueryText=dopamine&adv_kw_filter1=all&species=Homo+sapiens&adv_query_text3=&adv_query_text2=&adv_query_text1=&adv_boolean3=AND&adv_boolean2=AND&batchSpecies=&adv_boolean1=AND&chkBatchQueryText=false&kwfilter=all&SearchRequest.Common.PageNumber=1&formatType=default&) | x |
|  |  | PNMT | [Hs00160228_m1](https://products.appliedbiosystems.com/ab/en/US/adirect/ab?cmd=ABAssayDetailDisplay&assayID=Hs00160228_m1&Fs=y&catID=601267&SearchRequest.Common.PageNumber=1&assayType=&chkBatchQueryText=false&srchType=keyword&searchValue=Phenylethanolamine+N-methyltransferase&searchBy=all&) | x |
|  |  |  |  |  |
|  | Serotonergic Neurons | |  |  |
|  |  | TPH2 | [Hs00542783_m1](https://products.appliedbiosystems.com/ab/en/US/direct/ab?cmd=ABAssayDetailDisplay&assayID=Hs00542783_m1&Fs=y&adv_phrase3=EXACT&adv_phrase2=EXACT&adv_phrase1=EXACT&assayType=ge&catID=601267&SearchRequest.Common.SortSpec=&searchValue=null&MFCSpeciesType=null&searchBy=null&adv_kw_filter3=all&srchType=keyword&adv_kw_filter2=all&group1=noValue_limit_sets&SearchRequest.Common.QueryText=tryptophan+hydroxylase&adv_kw_filter1=all&adv_query_text3=&species=Homo+sapiens&adv_query_text2=&adv_query_text1=&paraTreeViewNode=null&adv_boolean3=AND&displayAdvSearchResults=&adv_boolean2=AND&adv_boolean1=AND&chkBatchQueryText=false&kwfilter=all&SearchRequest.Common.PageNumber=1&formatType=default&isSL=N&ASSAY_ATTRIBUTE=40_Inventoried&) | x |
|  |  |  |  |  |
|  | Motor Neuron Markers | |  |  |
|  |  | CHAT | [Hs00252848_m1](https://products.appliedbiosystems.com/ab/en/US/adirect/ab?cmd=ABAssayDetailDisplay&assayID=Hs00252848_m1&Fs=y&adv_phrase3=EXACT&adv_phrase2=EXACT&adv_phrase1=EXACT&assayType=ge&catID=601267&SearchRequest.Common.SortSpec=&searchValue=null&MFCSpeciesType=null&searchBy=null&adv_kw_filter3=all&srchType=keyword&adv_kw_filter2=all&group1=noValue_limit_sets&SearchRequest.Common.QueryText=chat&adv_kw_filter1=all&inventoried=1&adv_query_text3=&species=Homo+sapiens&adv_query_text2=&adv_query_text1=&paraTreeViewNode=null&adv_boolean3=AND&displayAdvSearchResults=&SearchRequest.Common.ResultsPerPage=25&adv_boolean2=AND&adv_boolean1=AND&chkBatchQueryText=false&kwfilter=all&SearchRequest.Common.PageNumber=1&formatType=default&isSL=N&ASSAY_ATTRIBUTE=40_Inventoried&) | x |
|  |  | HB9 | [Hs00232128_m1](https://products.appliedbiosystems.com/ab/en/US/adirect/ab?cmd=ABAssayDetailDisplay&assayID=Hs00232128_m1&Fs=y&adv_phrase3=EXACT&adv_phrase2=EXACT&adv_phrase1=EXACT&assayType=ge&catID=601267&MFCSpeciesType=null&adv_kw_filter3=all&srchType=keyword&adv_kw_filter2=all&group1=noValue_limit_sets&SearchRequest.Common.QueryText=Hb9&adv_kw_filter1=all&species=Homo+sapiens&adv_query_text3=&adv_query_text2=&adv_query_text1=&adv_boolean3=AND&adv_boolean2=AND&batchSpecies=&adv_boolean1=AND&chkBatchQueryText=false&kwfilter=all&SearchRequest.Common.PageNumber=1&formatType=default&ASSAY_ATTRIBUTE=40_Inventoried&) | x |
|  |  | NKX6.1 | [Hs00232355_m1](https://products.appliedbiosystems.com/ab/en/US/adirect/ab?cmd=ABAssayDetailDisplay&assayID=Hs00232355_m1&Fs=y&adv_phrase3=EXACT&adv_phrase2=EXACT&adv_phrase1=EXACT&assayType=ge&catID=601267&MFCSpeciesType=null&adv_kw_filter3=all&srchType=keyword&adv_kw_filter2=all&group1=noValue_limit_sets&SearchRequest.Common.QueryText=nkx6&adv_kw_filter1=all&species=Homo+sapiens&adv_query_text3=&searchType=keyword&adv_query_text2=&adv_query_text1=&adv_boolean3=AND&adv_boolean2=AND&batchSpecies=Homo+sapiens&adv_boolean1=AND&chkBatchQueryText=false&kwfilter=all&SearchRequest.Common.PageNumber=1&formatType=default&ASSAY_ATTRIBUTE=40_Inventoried&) | x |
|  |  |  |  |  |
|  | Astrocytes/Oligodendrocytes | |  |  |
|  |  | GFAP | [Hs00157674_m1](https://products.appliedbiosystems.com/ab/en/US/adirect/ab?cmd=ABAssayDetailDisplay&assayID=Hs00157674_m1&Fs=y&adv_phrase3=EXACT&adv_phrase2=EXACT&adv_phrase1=EXACT&assayType=ge&catID=601267&SearchRequest.Common.SortSpec=&searchValue=null&MFCSpeciesType=null&searchBy=null&adv_kw_filter3=all&srchType=keyword&adv_kw_filter2=all&group1=noValue_limit_sets&SearchRequest.Common.QueryText=gfap&adv_kw_filter1=all&inventoried=1&adv_query_text3=&species=Homo+sapiens&adv_query_text2=&adv_query_text1=&paraTreeViewNode=null&adv_boolean3=AND&displayAdvSearchResults=&SearchRequest.Common.ResultsPerPage=25&adv_boolean2=AND&adv_boolean1=AND&chkBatchQueryText=false&kwfilter=all&SearchRequest.Common.PageNumber=1&formatType=default&isSL=N&ASSAY_ATTRIBUTE=40_Inventoried&) | x |
|  |  | OLIG2 | [Hs00377820_m1](https://products.appliedbiosystems.com/ab/en/US/adirect/ab?cmd=ABAssayDetailDisplay&assayID=Hs00377820_m1&Fs=y&adv_phrase3=EXACT&adv_phrase2=EXACT&adv_phrase1=EXACT&assayType=ge&catID=601267&SearchRequest.Common.SortSpec=&searchValue=null&MFCSpeciesType=null&searchBy=null&adv_kw_filter3=all&srchType=keyword&adv_kw_filter2=all&group1=noValue_limit_sets&SearchRequest.Common.QueryText=olig2&adv_kw_filter1=all&inventoried=1&adv_query_text3=&species=Homo+sapiens&adv_query_text2=&adv_query_text1=&paraTreeViewNode=null&adv_boolean3=AND&displayAdvSearchResults=&SearchRequest.Common.ResultsPerPage=25&adv_boolean2=AND&adv_boolean1=AND&chkBatchQueryText=false&kwfilter=all&SearchRequest.Common.PageNumber=1&formatType=default&isSL=N&ASSAY_ATTRIBUTE=40_Inventoried&) | x |
|  |  |  |  |  |
|  | *Axon Guidance Related Primers* | |  |  |
|  |  | NEO1 | [Hs00170143_m1](https://products.appliedbiosystems.com/ab/en/US/direct/ab?cmd=ABAssayDetailDisplay&assayID=Hs00170143_m1&Fs=y&adv_phrase3=EXACT&adv_phrase2=EXACT&adv_phrase1=EXACT&assayType=ge&catID=601267&SearchRequest.Common.SortSpec=&searchValue=null&MFCSpeciesType=null&searchBy=null&adv_kw_filter3=all&srchType=keyword&adv_kw_filter2=all&group1=noValue_limit_sets&SearchRequest.Common.QueryText=neogenin&adv_kw_filter1=all&inventoried=1&adv_query_text3=&species=Homo+sapiens&adv_query_text2=&adv_query_text1=&paraTreeViewNode=null&adv_boolean3=AND&displayAdvSearchResults=&SearchRequest.Common.ResultsPerPage=25&adv_boolean2=AND&adv_boolean1=AND&chkBatchQueryText=false&kwfilter=all&SearchRequest.Common.PageNumber=1&formatType=default&isSL=N&ASSAY_ATTRIBUTE=40_Inventoried&) |  |
|  |  | NTN1 | [Hs00180355_m1](https://products.appliedbiosystems.com/ab/en/US/adirect/ab?cmd=ABAssayDetailDisplay&assayID=Hs00180355_m1&Fs=y&adv_phrase3=EXACT&adv_phrase2=EXACT&adv_phrase1=EXACT&assayType=ge&catID=601267&SearchRequest.Common.SortSpec=&searchValue=null&MFCSpeciesType=null&searchBy=null&adv_kw_filter3=all&srchType=keyword&adv_kw_filter2=all&group1=noValue&SearchRequest.Common.QueryText=ntn1&adv_kw_filter1=all&adv_query_text3=&species=Homo+sapiens&adv_query_text2=&adv_query_text1=&paraTreeViewNode=null&adv_boolean3=AND&displayAdvSearchResults=&adv_boolean2=AND&adv_boolean1=AND&chkBatchQueryText=false&kwfilter=all&SearchRequest.Common.PageNumber=1&formatType=default&isSL=N&) | x |
|  |  | NTN1 | [Hs00924151_m1](https://products.appliedbiosystems.com/ab/en/US/adirect/ab?cmd=ABAssayDetailDisplay&assayID=Hs00924151_m1&Fs=y&adv_phrase3=EXACT&adv_phrase2=EXACT&adv_phrase1=EXACT&assayType=ge&catID=601267&MFCSpeciesType=null&adv_kw_filter3=all&srchType=keyword&adv_kw_filter2=all&group1=noValue_limit_sets&SearchRequest.Common.QueryText=ntn1&adv_kw_filter1=all&species=Homo+sapiens&adv_query_text3=&adv_query_text2=&adv_query_text1=&adv_boolean3=AND&adv_boolean2=AND&batchSpecies=Homo+sapiens&adv_boolean1=AND&chkBatchQueryText=false&kwfilter=all&SearchRequest.Common.PageNumber=1&formatType=default&ASSAY_ATTRIBUTE=40_Inventoried&) | x |
|  |  | DCC | [Hs00180437_m1](https://products.appliedbiosystems.com/ab/en/US/adirect/ab?cmd=ABAssayDetailDisplay&assayID=Hs00180437_m1&Fs=y&adv_phrase3=EXACT&adv_phrase2=EXACT&adv_phrase1=EXACT&assayType=ge&catID=601267&SearchRequest.Common.SortSpec=&searchValue=null&MFCSpeciesType=null&searchBy=null&adv_kw_filter3=all&srchType=keyword&adv_kw_filter2=all&group1=noValue&SearchRequest.Common.QueryText=dcc&adv_kw_filter1=all&adv_query_text3=&species=Homo+sapiens&adv_query_text2=&adv_query_text1=&paraTreeViewNode=null&adv_boolean3=AND&displayAdvSearchResults=&adv_boolean2=AND&adv_boolean1=AND&chkBatchQueryText=false&kwfilter=all&SearchRequest.Common.PageNumber=2&formatType=default&isSL=N&) | x |
|  |  | ROBO1 | [Hs00268049_m1](https://products.appliedbiosystems.com/ab/en/US/direct/ab?cmd=ABAssayDetailDisplay&assayID=Hs00268049_m1&Fs=y&adv_phrase3=EXACT&adv_phrase2=EXACT&adv_phrase1=EXACT&assayType=ge&catID=601267&SearchRequest.Common.SortSpec=&searchValue=null&MFCSpeciesType=null&searchBy=null&adv_kw_filter3=all&srchType=keyword&adv_kw_filter2=all&group1=noValue_limit_sets&SearchRequest.Common.QueryText=robo1&adv_kw_filter1=all&inventoried=1&adv_query_text3=&species=Homo+sapiens&adv_query_text2=&adv_query_text1=&paraTreeViewNode=null&adv_boolean3=AND&displayAdvSearchResults=&SearchRequest.Common.ResultsPerPage=25&adv_boolean2=AND&adv_boolean1=AND&chkBatchQueryText=false&kwfilter=all&SearchRequest.Common.PageNumber=1&formatType=default&isSL=N&ASSAY_ATTRIBUTE=40_Inventoried&) | x |
|  |  | ROBO2 | [Hs00326067_m1](https://products.appliedbiosystems.com/ab/en/US/direct/ab?cmd=ABAssayDetailDisplay&assayID=Hs00326067_m1&Fs=y&adv_phrase3=EXACT&adv_phrase2=EXACT&adv_phrase1=EXACT&assayType=ge&catID=601267&SearchRequest.Common.SortSpec=&searchValue=null&MFCSpeciesType=null&searchBy=null&adv_kw_filter3=all&srchType=keyword&adv_kw_filter2=all&group1=noValue_limit_sets&SearchRequest.Common.QueryText=robo2&adv_kw_filter1=all&inventoried=1&adv_query_text3=&species=Homo+sapiens&adv_query_text2=&adv_query_text1=&paraTreeViewNode=null&adv_boolean3=AND&displayAdvSearchResults=&SearchRequest.Common.ResultsPerPage=25&adv_boolean2=AND&adv_boolean1=AND&chkBatchQueryText=false&kwfilter=all&SearchRequest.Common.PageNumber=1&formatType=default&isSL=N&ASSAY_ATTRIBUTE=40_Inventoried&) | x |
|  |  | ROBO3 | [Hs00223636_m1](https://products.appliedbiosystems.com/ab/en/US/direct/ab?cmd=ABAssayDetailDisplay&assayID=Hs00223636_m1&Fs=y&adv_phrase3=EXACT&adv_phrase2=EXACT&adv_phrase1=EXACT&assayType=ge&catID=601267&SearchRequest.Common.SortSpec=&searchValue=null&MFCSpeciesType=null&searchBy=null&adv_kw_filter3=all&srchType=keyword&adv_kw_filter2=all&group1=noValue_limit_sets&SearchRequest.Common.QueryText=robo3&adv_kw_filter1=all&inventoried=1&adv_query_text3=&species=Homo+sapiens&adv_query_text2=&adv_query_text1=&paraTreeViewNode=null&adv_boolean3=AND&displayAdvSearchResults=&SearchRequest.Common.ResultsPerPage=25&adv_boolean2=AND&adv_boolean1=AND&chkBatchQueryText=false&kwfilter=all&SearchRequest.Common.PageNumber=1&formatType=default&isSL=N&ASSAY_ATTRIBUTE=40_Inventoried&) | x |
|  |  | ROBO4 | [Hs00219408_m1](https://products.appliedbiosystems.com/ab/en/US/direct/ab?cmd=ABAssayDetailDisplay&assayID=Hs00219408_m1&Fs=y&adv_phrase3=EXACT&adv_phrase2=EXACT&adv_phrase1=EXACT&assayType=ge&catID=601267&SearchRequest.Common.SortSpec=&searchValue=null&MFCSpeciesType=null&searchBy=null&adv_kw_filter3=all&srchType=keyword&adv_kw_filter2=all&group1=noValue_limit_sets&SearchRequest.Common.QueryText=robo4&adv_kw_filter1=all&inventoried=1&adv_query_text3=&species=Homo+sapiens&adv_query_text2=&adv_query_text1=&paraTreeViewNode=null&adv_boolean3=AND&displayAdvSearchResults=&SearchRequest.Common.ResultsPerPage=25&adv_boolean2=AND&adv_boolean1=AND&chkBatchQueryText=false&kwfilter=all&SearchRequest.Common.PageNumber=1&formatType=default&isSL=N&ASSAY_ATTRIBUTE=40_Inventoried&) | x |
|  |  |  |  |  |
| ***Pathological Protein Primers*** | |  |  |  |
|  | park1 | SNCA | [Hs01103383_m1](https://products.appliedbiosystems.com/ab/en/US/adirect/ab?cmd=ABAssayDetailDisplay&assayID=Hs01103383_m1&Fs=y&adv_phrase3=EXACT&adv_phrase2=EXACT&adv_phrase1=EXACT&assayType=ge&catID=601267&SearchRequest.Common.SortSpec=&searchValue=null&MFCSpeciesType=null&searchBy=null&adv_kw_filter3=all&srchType=keyword&adv_kw_filter2=all&group1=noValue&SearchRequest.Common.QueryText=snca&adv_kw_filter1=all&inventoried=*&adv_query_text3=&species=Homo+sapiens&adv_query_text2=&adv_query_text1=&paraTreeViewNode=null&adv_boolean3=AND&displayAdvSearchResults=&SearchRequest.Common.ResultsPerPage=25&adv_boolean2=AND&adv_boolean1=AND&chkBatchQueryText=false&kwfilter=all&SearchRequest.Common.PageNumber=1&formatType=default&isSL=N&) | x |
|  | park2 | PARKIN | [Hs00247755_m1](https://products.appliedbiosystems.com/ab/en/US/direct/ab?cmd=ABAssayDetailDisplay&assayID=Hs00247755_m1&Fs=y&adv_phrase3=EXACT&adv_phrase2=EXACT&adv_phrase1=EXACT&assayType=ge&catID=601267&MFCSpeciesType=null&adv_kw_filter3=all&srchType=keyword&adv_kw_filter2=all&group1=noValue_limit_sets&SearchRequest.Common.QueryText=park2&adv_kw_filter1=all&species=Homo+sapiens&adv_query_text3=&adv_query_text2=&adv_query_text1=&adv_boolean3=AND&adv_boolean2=AND&batchSpecies=&adv_boolean1=AND&chkBatchQueryText=false&kwfilter=all&SearchRequest.Common.PageNumber=1&formatType=default&ASSAY_ATTRIBUTE=40_Inventoried&) | x |
|  | park3 | unknown | N/A |  |
|  | park4 | SNCA | already listed | x |
|  | park5 | UCHL1 | [Hs00188233_m1](https://products.appliedbiosystems.com/ab/en/US/direct/ab?cmd=ABAssayDetailDisplay&assayID=Hs00188233_m1&Fs=y&adv_phrase3=EXACT&adv_phrase2=EXACT&adv_phrase1=EXACT&assayType=ge&catID=601267&SearchRequest.Common.SortSpec=&searchValue=null&MFCSpeciesType=null&searchBy=null&adv_kw_filter3=all&srchType=keyword&adv_kw_filter2=all&group1=noValue_limit_sets&SearchRequest.Common.QueryText=park5&adv_kw_filter1=all&adv_query_text3=&species=Homo+sapiens&adv_query_text2=&adv_query_text1=&paraTreeViewNode=null&adv_boolean3=AND&displayAdvSearchResults=&adv_boolean2=AND&adv_boolean1=AND&chkBatchQueryText=false&kwfilter=all&SearchRequest.Common.PageNumber=1&formatType=default&isSL=N&ASSAY_ATTRIBUTE=40_Inventoried&) | x |
|  | park6 | PINK1 | [Hs00260868_m1](https://products.appliedbiosystems.com/ab/en/US/direct/ab?cmd=ABAssayDetailDisplay&assayID=Hs00260868_m1&Fs=y&adv_phrase3=EXACT&adv_phrase2=EXACT&adv_phrase1=EXACT&assayType=ge&catID=601267&SearchRequest.Common.SortSpec=&searchValue=null&MFCSpeciesType=null&searchBy=null&adv_kw_filter3=all&srchType=keyword&adv_kw_filter2=all&group1=noValue_limit_sets&SearchRequest.Common.QueryText=park6&adv_kw_filter1=all&adv_query_text3=&species=Homo+sapiens&adv_query_text2=&adv_query_text1=&paraTreeViewNode=null&adv_boolean3=AND&displayAdvSearchResults=&adv_boolean2=AND&adv_boolean1=AND&chkBatchQueryText=false&kwfilter=all&SearchRequest.Common.PageNumber=1&formatType=default&isSL=N&ASSAY_ATTRIBUTE=40_Inventoried&) | x |
|  | park7 | PARK7 | [Hs00697109_m1](https://products.appliedbiosystems.com/ab/en/US/direct/ab?cmd=ABAssayDetailDisplay&assayID=Hs00697109_m1&Fs=y&adv_phrase3=EXACT&adv_phrase2=EXACT&adv_phrase1=EXACT&assayType=ge&catID=601267&SearchRequest.Common.SortSpec=&searchValue=null&MFCSpeciesType=null&searchBy=null&adv_kw_filter3=all&srchType=keyword&adv_kw_filter2=all&group1=noValue_limit_sets&SearchRequest.Common.QueryText=park7&adv_kw_filter1=all&adv_query_text3=&species=Homo+sapiens&adv_query_text2=&adv_query_text1=&paraTreeViewNode=null&adv_boolean3=AND&displayAdvSearchResults=&adv_boolean2=AND&adv_boolean1=AND&chkBatchQueryText=false&kwfilter=all&SearchRequest.Common.PageNumber=1&formatType=default&isSL=N&ASSAY_ATTRIBUTE=40_Inventoried&) | x |
|  | park8 | LRRK2 | [Hs00411197_m1](https://products.appliedbiosystems.com/ab/en/US/direct/ab?cmd=ABAssayDetailDisplay&assayID=Hs00411197_m1&Fs=y&adv_phrase3=EXACT&adv_phrase2=EXACT&adv_phrase1=EXACT&assayType=ge&catID=601267&SearchRequest.Common.SortSpec=&searchValue=null&MFCSpeciesType=null&searchBy=null&adv_kw_filter3=all&srchType=keyword&adv_kw_filter2=all&group1=noValue_limit_sets&SearchRequest.Common.QueryText=park8&adv_kw_filter1=all&adv_query_text3=&species=Homo+sapiens&adv_query_text2=&adv_query_text1=&paraTreeViewNode=null&adv_boolean3=AND&displayAdvSearchResults=&adv_boolean2=AND&adv_boolean1=AND&chkBatchQueryText=false&kwfilter=all&SearchRequest.Common.PageNumber=1&formatType=default&isSL=N&ASSAY_ATTRIBUTE=40_Inventoried&) | x |
|  | park9 | ATP13A2 | [Hs00223032_m1](https://products.appliedbiosystems.com/ab/en/US/direct/ab?cmd=ABAssayDetailDisplay&assayID=Hs00223032_m1&Fs=y&adv_phrase3=EXACT&adv_phrase2=EXACT&adv_phrase1=EXACT&assayType=ge&catID=601267&SearchRequest.Common.SortSpec=&searchValue=null&MFCSpeciesType=null&searchBy=null&adv_kw_filter3=all&srchType=keyword&adv_kw_filter2=all&group1=noValue_limit_sets&SearchRequest.Common.QueryText=park9&adv_kw_filter1=all&adv_query_text3=&species=Homo+sapiens&adv_query_text2=&adv_query_text1=&paraTreeViewNode=null&adv_boolean3=AND&displayAdvSearchResults=&adv_boolean2=AND&adv_boolean1=AND&chkBatchQueryText=false&kwfilter=all&SearchRequest.Common.PageNumber=1&formatType=default&isSL=N&ASSAY_ATTRIBUTE=40_Inventoried&) | x |
|  | park10 | unknown | N/A |  |
|  | park11 | unknown | N/A |  |
|  | park12 | unknown | N/A |  |
|  | park13 | HTRA2 | [Hs00234883_m1](https://products.appliedbiosystems.com/ab/en/US/direct/ab?cmd=ABAssayDetailDisplay&assayID=Hs00234883_m1&Fs=y&adv_phrase3=EXACT&adv_phrase2=EXACT&adv_phrase1=EXACT&assayType=ge&catID=601267&SearchRequest.Common.SortSpec=&searchValue=null&MFCSpeciesType=null&searchBy=null&adv_kw_filter3=all&srchType=keyword&adv_kw_filter2=all&group1=noValue_limit_sets&SearchRequest.Common.QueryText=park13&adv_kw_filter1=all&adv_query_text3=&species=Homo+sapiens&adv_query_text2=&adv_query_text1=&paraTreeViewNode=null&adv_boolean3=AND&displayAdvSearchResults=&adv_boolean2=AND&adv_boolean1=AND&chkBatchQueryText=false&kwfilter=all&SearchRequest.Common.PageNumber=1&formatType=default&isSL=N&ASSAY_ATTRIBUTE=40_Inventoried&) | x |
|  |  |  |  |  |
| ***Oxidative Stress / Metabolic Primers*** | | |  |  |
|  |  | CASP9 | [Hs00154260_m1](https://products.appliedbiosystems.com/ab/en/US/adirect/ab?cmd=ABAssayDetailDisplay&assayID=Hs00154260_m1&Fs=y&adv_phrase3=EXACT&adv_phrase2=EXACT&adv_phrase1=EXACT&assayType=ge&catID=601267&SearchRequest.Common.SortSpec=&searchValue=null&MFCSpeciesType=null&searchBy=null&adv_kw_filter3=all&srchType=keyword&adv_kw_filter2=all&group1=noValue_limit_sets&SearchRequest.Common.QueryText=caspase+9&adv_kw_filter1=all&adv_query_text3=&species=Homo+sapiens&adv_query_text2=&adv_query_text1=&paraTreeViewNode=null&adv_boolean3=AND&displayAdvSearchResults=&adv_boolean2=AND&adv_boolean1=AND&chkBatchQueryText=false&kwfilter=all&SearchRequest.Common.PageNumber=1&formatType=default&isSL=N&ASSAY_ATTRIBUTE=40_Inventoried&) |  |
|  |  | DNAJA1 | [Hs00266011_m1](https://products.appliedbiosystems.com/ab/en/US/adirect/ab?cmd=ABAssayDetailDisplay&assayID=Hs00266011_m1&Fs=y&adv_phrase3=EXACT&adv_phrase2=EXACT&adv_phrase1=EXACT&assayType=ge&catID=601267&MFCSpeciesType=null&adv_kw_filter3=all&srchType=keyword&adv_kw_filter2=all&group1=noValue_limit_sets&SearchRequest.Common.QueryText=dnaja1&adv_kw_filter1=all&species=Homo+sapiens&adv_query_text3=&adv_query_text2=&adv_query_text1=&adv_boolean3=AND&adv_boolean2=AND&batchSpecies=&adv_boolean1=AND&chkBatchQueryText=false&kwfilter=all&SearchRequest.Common.PageNumber=1&formatType=default&ASSAY_ATTRIBUTE=40_Inventoried&) | x |
|  |  | GPX1 | [Hs00829989_gH](https://products.appliedbiosystems.com/ab/en/US/adirect/ab?cmd=ABAssayDetailDisplay&assayID=Hs00829989_gH&Fs=y&adv_phrase3=EXACT&adv_phrase2=EXACT&adv_phrase1=EXACT&assayType=ge&catID=601267&SearchRequest.Common.SortSpec=&searchValue=null&MFCSpeciesType=null&searchBy=null&adv_kw_filter3=all&srchType=keyword&adv_kw_filter2=all&group1=noValue_limit_sets&SearchRequest.Common.QueryText=gpx1&adv_kw_filter1=all&adv_query_text3=&species=Homo+sapiens&adv_query_text2=&adv_query_text1=&paraTreeViewNode=null&adv_boolean3=AND&displayAdvSearchResults=&adv_boolean2=AND&adv_boolean1=AND&chkBatchQueryText=false&kwfilter=all&SearchRequest.Common.PageNumber=1&formatType=default&isSL=N&ASSAY_ATTRIBUTE=40_Inventoried&) | x |
|  |  | GSTP1 | [Hs00168310_m1](https://products.appliedbiosystems.com/ab/en/US/adirect/ab?cmd=ABAssayDetailDisplay&assayID=Hs00168310_m1&Fs=y&adv_phrase3=EXACT&adv_phrase2=EXACT&adv_phrase1=EXACT&assayType=ge&catID=601267&SearchRequest.Common.SortSpec=&searchValue=null&MFCSpeciesType=null&searchBy=null&adv_kw_filter3=all&srchType=keyword&adv_kw_filter2=all&group1=noValue_limit_sets&SearchRequest.Common.QueryText=gstp1&adv_kw_filter1=all&adv_query_text3=&species=Homo+sapiens&adv_query_text2=&adv_query_text1=&paraTreeViewNode=null&adv_boolean3=AND&displayAdvSearchResults=&adv_boolean2=AND&adv_boolean1=AND&chkBatchQueryText=false&kwfilter=all&SearchRequest.Common.PageNumber=1&formatType=default&isSL=N&ASSAY_ATTRIBUTE=40_Inventoried&) | x |
|  |  | HMOX1 | [Hs00157965_m1](https://products.appliedbiosystems.com/ab/en/US/adirect/ab?cmd=ABAssayDetailDisplay&assayID=Hs00157965_m1&Fs=y&adv_phrase3=EXACT&adv_phrase2=EXACT&adv_phrase1=EXACT&assayType=ge&catID=601267&SearchRequest.Common.SortSpec=&searchValue=null&MFCSpeciesType=null&searchBy=null&adv_kw_filter3=all&srchType=keyword&adv_kw_filter2=all&group1=noValue&SearchRequest.Common.QueryText=heme+oxygenase&adv_kw_filter1=all&inventoried=*&adv_query_text3=&species=Homo+sapiens&adv_query_text2=&adv_query_text1=&paraTreeViewNode=null&adv_boolean3=AND&displayAdvSearchResults=&SearchRequest.Common.ResultsPerPage=25&adv_boolean2=AND&adv_boolean1=AND&chkBatchQueryText=false&kwfilter=all&SearchRequest.Common.PageNumber=1&formatType=default&isSL=N&) | x |
|  |  | HMOX2 | [Hs00157969_m1](https://products.appliedbiosystems.com/ab/en/US/adirect/ab?cmd=ABAssayDetailDisplay&assayID=Hs00157969_m1&Fs=y&adv_phrase3=EXACT&adv_phrase2=EXACT&adv_phrase1=EXACT&assayType=ge&catID=601267&SearchRequest.Common.SortSpec=&searchValue=null&MFCSpeciesType=null&searchBy=null&adv_kw_filter3=all&srchType=keyword&adv_kw_filter2=all&group1=noValue&SearchRequest.Common.QueryText=hmox2&adv_kw_filter1=all&inventoried=*&adv_query_text3=&species=Homo+sapiens&adv_query_text2=&adv_query_text1=&paraTreeViewNode=null&adv_boolean3=AND&displayAdvSearchResults=&SearchRequest.Common.ResultsPerPage=25&adv_boolean2=AND&adv_boolean1=AND&chkBatchQueryText=false&kwfilter=all&SearchRequest.Common.PageNumber=1&formatType=default&isSL=N&) | x |
|  |  | HSPA1A | [Hs00359147_s1](https://products.appliedbiosystems.com/ab/en/US/adirect/ab?cmd=ABAssayDetailDisplay&assayID=Hs00359147_s1&Fs=y&adv_phrase3=EXACT&adv_phrase2=EXACT&adv_phrase1=EXACT&assayType=ge&catID=601267&SearchRequest.Common.SortSpec=&searchValue=null&MFCSpeciesType=null&searchBy=null&adv_kw_filter3=all&srchType=keyword&adv_kw_filter2=all&group1=noValue_limit_sets&SearchRequest.Common.QueryText=Hsp70&adv_kw_filter1=all&adv_query_text3=&species=Homo+sapiens&adv_query_text2=&adv_query_text1=&paraTreeViewNode=null&adv_boolean3=AND&displayAdvSearchResults=&adv_boolean2=AND&adv_boolean1=AND&chkBatchQueryText=false&kwfilter=all&SearchRequest.Common.PageNumber=1&formatType=default&isSL=N&ASSAY_ATTRIBUTE=40_Inventoried&) | x |
|  |  | HSPB1 | [Hs03044127_g1](https://products.appliedbiosystems.com/ab/en/US/adirect/ab?cmd=ABAssayDetailDisplay&assayID=Hs03044127_g1&Fs=y&adv_phrase3=EXACT&adv_phrase2=EXACT&adv_phrase1=EXACT&assayType=ge&catID=601267&MFCSpeciesType=null&adv_kw_filter3=all&srchType=keyword&adv_kw_filter2=all&group1=noValue_limit_sets&SearchRequest.Common.QueryText=hsp25&adv_kw_filter1=all&species=Homo+sapiens&adv_query_text3=&adv_query_text2=&adv_query_text1=&adv_boolean3=AND&adv_boolean2=AND&batchSpecies=&adv_boolean1=AND&chkBatchQueryText=false&kwfilter=all&SearchRequest.Common.PageNumber=1&formatType=default&ASSAY_ATTRIBUTE=40_Inventoried&) | x |
|  |  | MAOA | [Hs00165140_m1](https://products.appliedbiosystems.com/ab/en/US/adirect/ab?cmd=ABAssayDetailDisplay&assayID=Hs00165140_m1&Fs=y&adv_phrase3=EXACT&adv_phrase2=EXACT&adv_phrase1=EXACT&assayType=ge&catID=601267&MFCSpeciesType=null&adv_kw_filter3=all&srchType=keyword&adv_kw_filter2=all&group1=noValue&SearchRequest.Common.QueryText=monoamine+oxidase&adv_kw_filter1=all&species=Homo+sapiens&adv_query_text3=&adv_query_text2=&adv_query_text1=&adv_boolean3=AND&adv_boolean2=AND&batchSpecies=&adv_boolean1=AND&chkBatchQueryText=false&kwfilter=all&SearchRequest.Common.PageNumber=1&formatType=default&) | x |
|  |  | MAOB | [Hs00168533_m1](https://products.appliedbiosystems.com/ab/en/US/adirect/ab?cmd=ABAssayDetailDisplay&assayID=Hs00168533_m1&Fs=y&adv_phrase3=EXACT&adv_phrase2=EXACT&adv_phrase1=EXACT&assayType=ge&catID=601267&MFCSpeciesType=null&adv_kw_filter3=all&srchType=keyword&adv_kw_filter2=all&group1=noValue&SearchRequest.Common.QueryText=monoamine+oxidase&adv_kw_filter1=all&species=Homo+sapiens&adv_query_text3=&adv_query_text2=&adv_query_text1=&adv_boolean3=AND&adv_boolean2=AND&batchSpecies=&adv_boolean1=AND&chkBatchQueryText=false&kwfilter=all&SearchRequest.Common.PageNumber=1&formatType=default&) | x |
|  |  | MT1A | [Hs00831826_s1](https://products.appliedbiosystems.com/ab/en/US/adirect/ab?cmd=ABAssayDetailDisplay&assayID=Hs00831826_s1&Fs=y&adv_phrase3=EXACT&adv_phrase2=EXACT&adv_phrase1=EXACT&assayType=ge&catID=601267&SearchRequest.Common.SortSpec=&searchValue=null&MFCSpeciesType=null&searchBy=null&adv_kw_filter3=all&srchType=keyword&adv_kw_filter2=all&group1=noValue_limit_sets&SearchRequest.Common.QueryText=mt1&adv_kw_filter1=all&adv_query_text3=&species=Homo+sapiens&adv_query_text2=&adv_query_text1=&paraTreeViewNode=null&adv_boolean3=AND&displayAdvSearchResults=&adv_boolean2=AND&adv_boolean1=AND&chkBatchQueryText=false&kwfilter=all&SearchRequest.Common.PageNumber=1&formatType=default&isSL=N&ASSAY_ATTRIBUTE=40_Inventoried&) | x |
|  |  | MT2A | [Hs02379661_g1](https://products.appliedbiosystems.com/ab/en/US/adirect/ab?cmd=ABAssayDetailDisplay&assayID=Hs02379661_g1&Fs=y&adv_phrase3=EXACT&adv_phrase2=EXACT&adv_phrase1=EXACT&assayType=ge&catID=601267&SearchRequest.Common.SortSpec=&searchValue=null&MFCSpeciesType=null&searchBy=null&adv_kw_filter3=all&srchType=keyword&adv_kw_filter2=all&group1=noValue_limit_sets&SearchRequest.Common.QueryText=mt2&adv_kw_filter1=all&adv_query_text3=&species=Homo+sapiens&adv_query_text2=&adv_query_text1=&paraTreeViewNode=null&adv_boolean3=AND&displayAdvSearchResults=&adv_boolean2=AND&adv_boolean1=AND&chkBatchQueryText=false&kwfilter=all&SearchRequest.Common.PageNumber=1&formatType=default&isSL=N&ASSAY_ATTRIBUTE=40_Inventoried&) | x |
|  |  | NOX1 | [Hs00246589_m1](https://products.appliedbiosystems.com/ab/en/US/adirect/ab?cmd=ABAssayDetailDisplay&assayID=Hs00246589_m1&Fs=y&adv_phrase3=EXACT&adv_phrase2=EXACT&adv_phrase1=EXACT&assayType=ge&catID=601267&SearchRequest.Common.SortSpec=&searchValue=null&MFCSpeciesType=null&searchBy=null&adv_kw_filter3=all&srchType=keyword&adv_kw_filter2=all&group1=noValue_limit_sets&SearchRequest.Common.QueryText=nadph+oxidase&adv_kw_filter1=all&adv_query_text3=&species=Homo+sapiens&adv_query_text2=&adv_query_text1=&paraTreeViewNode=null&adv_boolean3=AND&displayAdvSearchResults=&adv_boolean2=AND&adv_boolean1=AND&chkBatchQueryText=false&kwfilter=all&SearchRequest.Common.PageNumber=1&formatType=default&isSL=N&ASSAY_ATTRIBUTE=40_Inventoried&) | x |
|  |  | SOD2 | [Hs00167309_m1](https://products.appliedbiosystems.com/ab/en/US/adirect/ab?cmd=ABAssayDetailDisplay&assayID=Hs00167309_m1&Fs=y&adv_phrase3=EXACT&adv_phrase2=EXACT&adv_phrase1=EXACT&assayType=ge&catID=601267&MFCSpeciesType=null&adv_kw_filter3=all&srchType=keyword&adv_kw_filter2=all&group1=noValue&SearchRequest.Common.QueryText=superoxide+dismutase&adv_kw_filter1=all&species=Homo+sapiens&adv_query_text3=&adv_query_text2=&adv_query_text1=&adv_boolean3=AND&adv_boolean2=AND&batchSpecies=&adv_boolean1=AND&chkBatchQueryText=false&kwfilter=all&SearchRequest.Common.PageNumber=1&formatType=default&) | x |

**Primers for Imprinting**

H19 F6005 (1) aggtgttttagttttatggatgatgg ^2^

H19 R6326 (1) (2) tcctataaatatcctattcccaaataacc [AF087017](http://hmg.oxfordjournals.org/cgi/external_ref?access_num=AF087017&link_type=GEN)

H19 F6115 (2) tgtatagtatatgggtatttttggaggttt 6005–6326

MEST/PEG1 F609 (1)(2) tygttgttggttagttttgtayggtt ^2^

MEST/PEG1 R898 (1) aaaaataacaccccctcctcaaat [Y10620](http://hmg.oxfordjournals.org/cgi/external_ref?access_num=Y10620&link_type=GEN)

MEST/PEG1 R827 (2) cccaaaaacaaccccaactc 609–898

SNRPN SNIF (1) TTAGGTTATTTYGGTGAGGGAGGG ^3^; 15489–15515

SNRPN SNIIR (1)(2) ACCACCRACACT*_x_*AG*_y_*TAACCTTACC [U41384](http://hmg.oxfordjournals.org/cgi/external_ref?access_num=U41384&link_type=GEN); 15280–15304

SNRPN SNIIF (2) AGGGAA*_x_*TTC*_y_*GGATTTTTGTATTG 15473–15495

KCNQ1 Bi-F GGGGTTGGTAGTAGTGGTTG ^4^; 40 cycles

KCNQ1 Bi-R CRCCCTCCRCCAACTCCAAC [AJ006345](http://www.ncbi.nlm.nih.gov/entrez/query.fcgi?db=Nucleotide&cmd=Search&term=AJ006345); 1.5 mM MgCl

KNCQ1OT1 LITBisF (1) GGGGGTTTTTTAGTATGGTTTTTTTT ^5^; 67934–7959

KNCQ1OT1 LITRV (2)(2) CACTACCCAAACCAAACTACACTAC U90095;8202–68225

KNCQ1OT1 LITFI (2) GGTTTTTTTTATTTTTTTGGGAGGGTT TG 68070–68098

x,y = mismatches. Y = C or T. R = G or A. N = C, T, G, or A.

**References**

1. Deb-Rinker, P., Ly, D., Jezierski, A., Sikorska, M. & Walker, P.R. Sequential DNA methylation of the Nanog and Oct-4 upstream regions in human NT2 cells during neuronal differentiation. *J Biol Chem* **280**, 6257-6260 (2005).

2. Kerjean, A. et al. Establishment of the paternal methylation imprint of the human H19 and MEST/PEG1 genes during spermatogenesis. *Human molecular genetics* **9**, 2183-2187 (2000).

3. Geuns, E., De Rycke, M., Van Steirteghem, A. & Liebaers, I. Methylation imprints of the imprint control region of the SNRPN-gene in human gametes and preimplantation embryos. *Human molecular genetics* **12**, 2873-2879 (2003).

4. Monk, D. et al. Limited evolutionary conservation of imprinting in the human placenta. *Proceedings of the National Academy of Sciences of the United States of America* **103**, 6623-6628 (2006).

5. Geuns, E., Hilven, P., Van Steirteghem, A., Liebaers, I. & De Rycke, M. Methylation analysis of KvDMR1 in human oocytes. *Journal of medical genetics* **44**, 144-147 (2007).
